# A simple and versatile design concept for fluorophore derivatives with intramolecular photostabilization

**DOI:** 10.1038/ncomms10144

**Published:** 2016-01-11

**Authors:** Jasper H. M. van der Velde, Jens Oelerich, Jingyi Huang, Jochem H. Smit, Atieh Aminian Jazi, Silvia Galiani, Kirill Kolmakov, Giorgos Gouridis, Christian Eggeling, Andreas Herrmann, Gerard Roelfes, Thorben Cordes

**Affiliations:** 1 Molecular Microscopy Research Group, Zernike Institute for Advanced Materials, University of Groningen, Nijenborgh 4, 9747 AG Groningen, The Netherlands; 2 Stratingh Institute for Chemistry, University of Groningen, Nijenborgh 4, 9747 AG Groningen, The Netherlands; 3 Department of Polymer Chemistry, Zernike Institute for Advanced Materials, University of Groningen, Nijenborgh 4, 9747 AG Groningen, The Netherlands; 4 MRC Human Immunology Unit, Weatherall Institute of Molecular Medicine, University of Oxford, Headley Way, Oxford OX3 9DS, UK; 5 Department NanoBiophotonics, Max-Planck-Institute of Molecular Medicine, Am Fassberg 1, 37077 Goettingen, Germany

## Abstract

Intramolecular photostabilization via triple-state quenching was recently revived as a tool to impart synthetic organic fluorophores with ‘self-healing’ properties. To date, utilization of such fluorophore derivatives is rare due to their elaborate multi-step synthesis. Here we present a general strategy to covalently link a synthetic organic fluorophore simultaneously to a photostabilizer and biomolecular target via unnatural amino acids. The modular approach uses commercially available starting materials and simple chemical transformations. The resulting photostabilizer–dye conjugates are based on rhodamines, carbopyronines and cyanines with excellent photophysical properties, that is, high photostability and minimal signal fluctuations. Their versatile use is demonstrated by single-step labelling of DNA, antibodies and proteins, as well as applications in single-molecule and super-resolution fluorescence microscopy. We are convinced that the presented scaffolding strategy and the improved characteristics of the conjugates in applications will trigger the broader use of intramolecular photostabilization and help to emerge this approach as a new gold standard.

## Abstract (web summary)

Synthetic organic fluorophores are powerful tools for bioimaging, but frequently display shortened observation times and signal fluctuations. Here, the authors report a general method to covalently label a biomolecule with a fluorophore and photostabilizer, reducing unwanted photophysical effects by intramolecular quenching of reactive fluorophore states.

## Graphical abstract

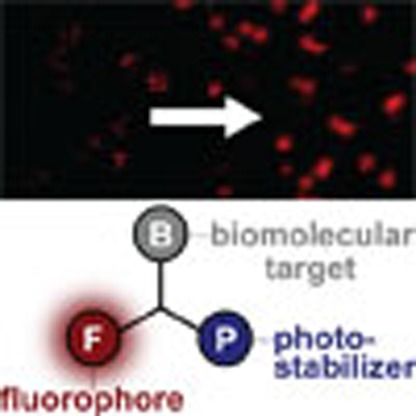

Organic fluorophores are a major driving force for the recent success of fluorescence-based methods, but they intrinsically suffer from transient excursions to dark states (blinking) and irreversible destruction (photobleaching)^1^,^2^. Both processes fundamentally limit their applicability and have, for a long time, hampered the development of advanced microscopy techniques with single-molecule sensitivity^2^ or optical super-resolution <250 nm (refs 2, 3, 4). Lüttke and colleagues^5^ introduced covalent binding of triplet-state quenchers and singlet-oxygen scavengers^6^ to organic fluorophores as a strategy to reduce the above mentioned effects. Such photostabilizer–dye conjugates with intramolecular triplet-state quenching have ‘self-healing’^7^ or ‘self-protecting’^8^ properties, preventing photodamage without the use of solution additives. This non-invasive strategy has clear advantages compared with commonly used approaches, where micro- to millimolar concentrations of organic compounds are added to the buffer system^9^,^10^,^11^,^12^,^13^,^14^,^15^.

Intramolecular photostabilization was recently revived independently by two groups^16^,^17^,^18^,^19^, to reduce photobleaching and blinking even in demanding applications such as single-molecule fluorescence microscopy^16^,^17^,^18^,^19^,^20^ or *in vivo* imaging^16^. It was also shown that efficient intramolecular photostabilization can be achieved without additives in the buffer system^18^, minimizing potential influences on the biological system of interest. Applications of intramolecular photostabilization in biophysical or microscopy research are, however, still limited to proof-of-principle studies^5^,^6^,^16^,^17^,^18^,^19^. This is mainly due to the synthetic effort of photostabilizer–dye conjugates, which thus far requires a multi-step synthesis route^16^,^17^. These synthetic challenges represent a fundamental hurdle for researchers with limited organic chemistry experience to use this concept. Moreover, only a small number of bifunctional cyanine derivatives are currently available to synthesize photostabilizer–dye conjugates on specific biomolecular targets (DNA, RNA, proteins and antibodies)^16^,^17^, strongly restricting the choice of fluorophore type (chemical structure, redox potential, water solubility and so on) and photophysical properties (colour, brightness, fluorescence lifetime and so on). Especially the available cyanine fluorophores suffer from limited brightness and signal-to-noise ratio (SNR) due to *cis*/*trans* isomerization^21^, a fact that emphasizes the urgent need for a synthetic strategy to study and use other classes of organic fluorophores via intramolecular photostabilization^18^,^19^,^20^,^21^,^22^.

Here we introduce a versatile and simple design concept to synthesize photostabilizer–dye conjugates on a specific biomolecular target using unnatural amino acids (UAAs)^23^. UAAs were chosen as a scaffold that links multiple chemical units, that is, the fluorophore and photostabilizer to a specific target. The presented conjugation strategy is based on well-known chemical reactions with (commercially available derivatives of) synthetic organic fluorophores, photostabilizers and UAAs, which can be bound as a single moiety to a biomolecular target. Depending on the UAA scaffold, the chemical nature of the functional groups can be *N*-hydroxysuccinimid esters (NHS), alkynes, azides or other bio-orthogonal reactive functionalities. Two different UAAs were used to bind different rhodamine, carbopyronine and cyanine fluorophores (Alexa555, RhodamineB, KK114, ATTO647N and Cy5) covalently to a photostabilizer on distinct biomolecular targets. The synthesized fluorophore derivatives comprise of either a reducing or oxidizing photostabilizer in the form of the antioxidant Trolox (TX) or a nitrophenyl group^10^,^12^. We characterized their photophysical properties with single-molecule fluorescence microscopy on the biomolecular target DNA and observed significant increases in photostability for all compounds including suppression of triplet-based blinking. Secondly, photostabilizer–dye conjugates were synthesized, which allow labelling of biomolecules (DNA, antibodies and proteins) in a single step as with commercially available fluorophores. Finally, we demonstrate state-of-the-art applications of photostabilizer–dye conjugates in single-molecule Förster resonance energy transfer (smFRET) and super-resolution stimulated emission depletion (STED) microscopy, with significantly increased sensitivity and photostability of the conjugates compared to their non-stabilized parent fluorophores.

## Results

### Amino acids for intramolecular photostabilization

**Figure 1:**
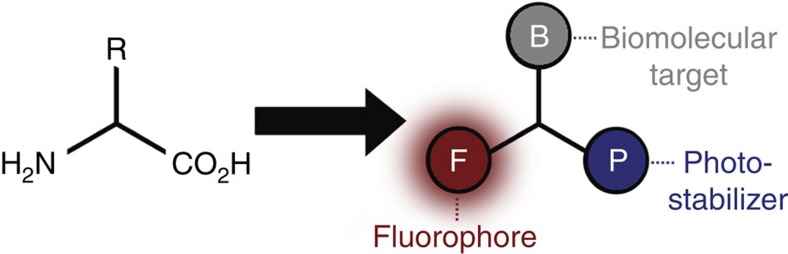
Design concept for photostabilizer–dye conjugates. UAAs are used to combine an organic fluorophore covalently with a photostabilizer on a biomolecular target or linker structure.

Figure 1 shows the central idea to use UAAs as a scaffold that links multiple chemical units, that is, fluorophore and photostabilizer onto a specific biomolecular target. The conjugation strategy is based on established chemical reactions (amide bond formation or click chemistry) using commercially available derivatives of synthetic organic fluorophores (F), photostabilizers (P) and UAAs.

**Figure 2:**
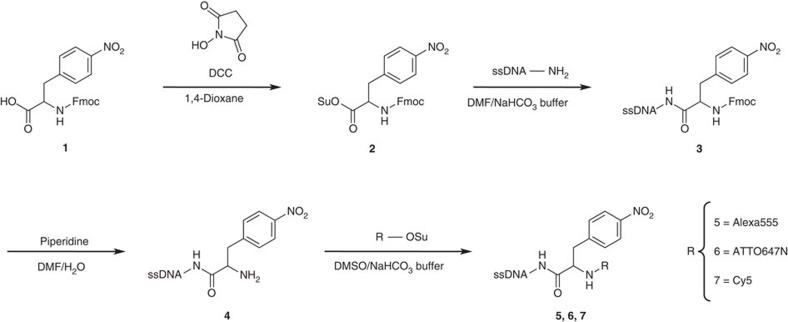
Synthesis route towards photostabilizer–dye conjugates on nucleic acids. NPA-based fluorophores of Alexa555, ATTO647N and Cy5 on ssDNA. Fmoc, fluorenylmethyloxycarbonyl; DCC, *N*-*N*’-dicyclohexylcarbodiimide; Su, succinimide; DMF, dimethylformamide; NaHCO_3_ buffer, 200 mM sodium hydrogen carbonate buffer pH 8.35; TBTA, tris[(1-benzyl-1H-1,2,3-triazol-4-yl)methyl]amine; TEAA buffer, 50 mM triethylammonium acetate buffer, pH 7.0.

As a proof-of-concept, we synthesized photostabilizer–dye conjugates of fluorophores that could thus far not be tested for intramolecular photostabilization due to limited scaffolding options. We focused on rhodamines and carbopyronines, that is, fluorophores from the ATTO and Alexa series, which are extremely popular for (life science) applications. *(S)*-Nitrophenylalanine, NPA (Fig. 2), was used as a scaffold for the first generation of compounds, which consists of single-stranded DNA (ssDNA) as the biomolecular target, a commercially available organic fluorophore (Alexa555, ATTO647N and Cy5) and the *p*-nitrophenyl group of the known photostabilizer NPA^16^,^17^,^19^ (Fig. 2, compounds **5**, **6** and **7**). The cyanine fluorophore Cy5 served as a ‘positive control’ experiment that permits to benchmark the effects of intramolecular photostabilization for different fluorophore classes with respect to published studies^16^,^17^,^18^,^19^,^20^. The used DNA and its specific base sequence was selected because of the detailed photophysical characterization of various fluorophores on this target^11^,^18^,^19^,^24^,^25^,^26^,^27^.

The synthesis shown in Fig. 2 was conducted starting from commercially available fluorenylmethyloxycarbonyl (Fmoc)-protected NPA (**1**), which was converted into the corresponding NHS-ester derivative (**2**) to react with a 5′-aminoalkyl functionalized ssDNA, yielding **3**. Subsequent Fmoc deprotection of **3** was followed by a reaction of the resulting primary amine (**4**) with the commercially available NHS-ester derivative of Alexa555, ATTO647N or Cy5, to yield **5**, **6** and **7**, respectively. All compounds comprise an organic fluorophore and a *p*-nitrophenyl group for intramolecular photostabilization linked to the biomolecular ssDNA target (Figs 1 and 2) and are abbreviated ‘NPA fluorophore’ throughout the text. The non-stabilized control compounds were obtained via a direct reaction of the NHS-ester-activated fluorophores with the respective amino-modified ssDNA-NH_2_. The DNA–photostabilizer–dye conjugates were isolated by high-performance liquid chromatography (HPLC; see Supplementary Fig. 1) and characterized by ultraviolet–visible absorption spectroscopy and mass spectrometry (Supplementary Figs 1 and 2). After purification, all compounds elute as single peaks in the HPLC chromatogram (Supplementary Fig. 1b,c) and display ultraviolet–visible absorption maxima characteristic for DNA (∼260 nm) and the respective chromophore (∼550/∼650 nm) (Supplementary Fig. 2a). In addition, matrix-assisted laser desorption/ionization time-of-flight (MALDI-TOF) mass spectrometry reveals a significant mass increase of the DNA–dye conjugates compared with the non-modified DNA (Supplementary Fig. 2b). See the Methods section and the Supplementary Information for further details on chemical synthesis and characterization of functionalized oligonucleotides and reactive precursor molecules.

Next, single-molecule fluorescence microscopy was used to benchmark the potential of the scaffolding approach with respect to photophysical parameters. Confocal scanning microscopy^11^,^12^,^18^,^24^ was used to investigate signal fluctuations and fluorescence lifetime, whereas total-internal-reflection fluorescence (TIRF) microscopy^11^,^18^ was employed to obtain quantitative photophysical values such as bleaching lifetime, fluorescence count rate, total number of photons and SNR ratio. The different fluorophore derivatives were immobilized according to published procedures on a streptavidin-coated microscope coverslip by hybridization to form double-stranded DNA (dsDNA) comprising a 3′-terminal biotin unit (Supplementary Figs 3a–11a)^18^,^19^. All experiments described in this section were performed in the absence of oxygen, to minimize the convolution of triplet-state quenching by molecular oxygen and intramolecular photostabilization by NPA.

**Figure 3:**
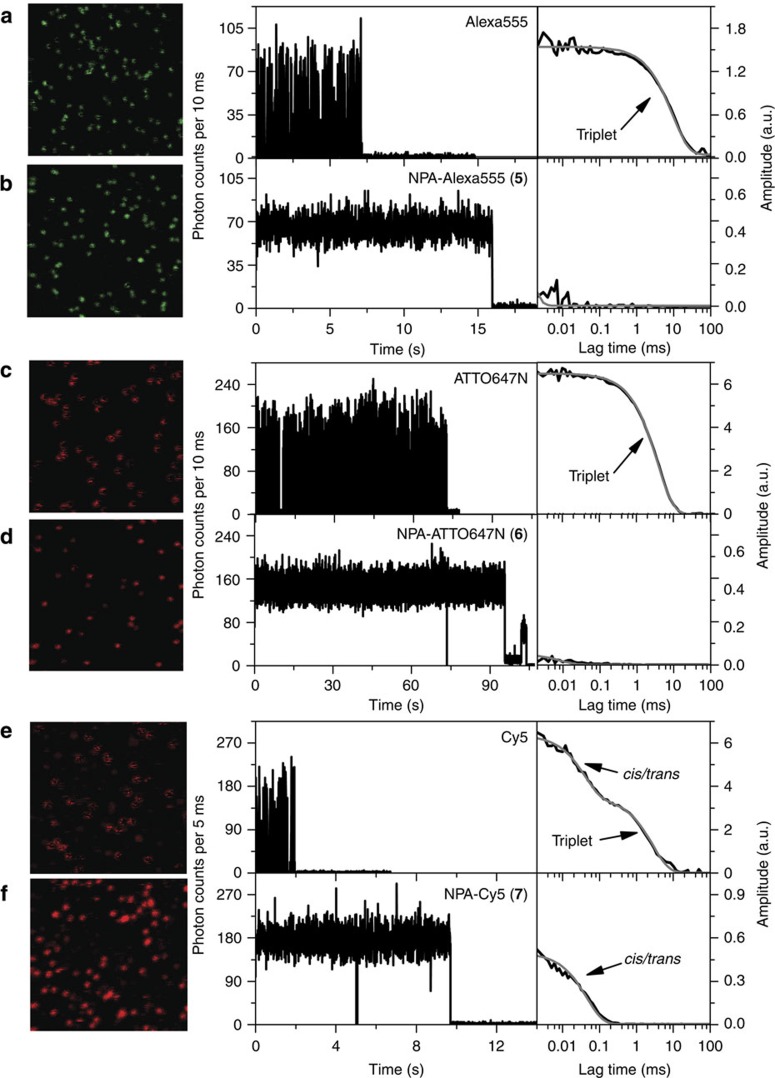
Photophysical characterization of photostabilizer–dye conjugates with confocal microscopy. All data was recorded with a home-built confocal microscope with a sample in aqueous PBS buffer at pH 7.4 in the absence of oxygen. Each panel shows a representative overview image (10 × 10 μm, 50 nm pixel size, 2 ms per pixel) with spots from individual immobilized fluorophores (left), a fluorescence time trace (middle) and the corresponding autocorrelation decay in black, with according fits in grey (right). (**a**,**b**) Alexa555 and NPA–Alexa555 (image intensity scale from 3 to 60 counts, excitation intensity of ≈0.3 kW cm^−2^ at 532 nm). (**c**,**d**) ATTO647N and NPA–ATTO647N (image intensity scale from 5 to 100 counts, excitation intensity of ≈0.66 kW cm^−2^ at 640 nm). (**e**,**f**) Cy5 and NPA–Cy5 image intensity scale from 10 to 300 counts, excitation intensity of 4 kW cm^−2^ at 640 nm. Further experimental details and data for each fluorophore can be found in the Methods section and Supplementary Figs 3–9.

Using confocal microscopy, single immobilized fluorophores on DNA were identified as spots in the images and fluorescence time traces were recorded for each of the spots using single-photon counting detectors. The length of the fluorescence time traces gave an estimate of the photostability (the longer, the more stable), whereas fluctuations in the time traces indicated blinking due to transient population of dark states (such as the triplet state). The calculation of the autocorrelation function of the individual fluorescence time traces revealed the underlying time constants of the blinking kinetics (Fig. 3) and time-correlated single-photon counting was used to determine the fluorescence lifetime. The non-stabilized fluorophores, showed fast photobleaching and pronounced blinking on the corresponding dsDNA in deoxygenated PBS buffer (Fig. 3a,c,e and Supplementary Figs 3d, 5d and 7d), whereas the NPA derivatives showed a stable fluorescence signal over extended periods of time (Fig. 3b,d,f and Supplementary Figs 4d, 6d and 7d).

Fluorescence time traces of Alexa555, a rhodamine-based fluorophore, were characterized by short observation times and pronounced blinking on the timescale of 22±6 ms in deoxygenated PBS buffer (Fig. 3a and Supplementary Fig. 3). This is a typical behaviour for rhodamines and is caused by population of the triplet state^4^. The fluorescence lifetime of the sample was found to be 1.2±0.3 ns (Supplementary Fig. 3). On conjugation of Alexa555 to the NPA scaffold (NPA–Alexa555, compound **5**), the triplet-induced blinking diminished (negligible autocorrelation) and the overall fluorophore photostability and count rate increased to several seconds and 5–10 kHz, respectively, at ∼0.3 kW cm^−2^ irradiance (Fig. 3b and Supplementary Fig. 4). Despite the overall increase in count rate (mainly due to abolishment of dark state transitions), the fluorescence lifetime of NPA–Alexa555 decreased to 0.9±0.1 ns compared with the parent fluorophore, indicating the presence of singlet quenching by the NPA scaffold (Supplementary Fig. 4).

The photophysical behaviour of ATTO647N in deoxygenated PBS buffer was characterized by a mixture of short and long photobleaching times ranging from seconds to minutes with pronounced blinking on the millisecond timescale (Fig. 3c and Supplementary Fig. 5). The off-state lifetime associated with the blinking events was found to be 29±5 ms and is attributed to the triplet state (Fig. 3c and Supplementary Fig. 5e)^4^. This and the observed fluorescence lifetime of 4.2±0.4 ns are in agreement with literature^11^,^28^. Covalent binding of NPA to ATTO647N resulted in a homogeneous, bright, non-blinking and prolonged fluorescence emission (Fig. 3d). The fluorescence lifetime was reduced to 3.4±0.2 ns for the majority of traces, again indicating dynamic singlet quenching by the NPA scaffold. Notably, >50% of the observed NPA–ATTO647N molecules did not photobleach within the 2 min observation period at excitation intensities of ≈0.66 kW cm^−2^ (see Fig. 3d and Supplementary Fig. 6). In addition, a small fraction (<30%) of NPA–ATTO647N molecules showed a reduced lifetime of around 2.6 ns in combination with a lower count rate pointing to stronger singlet quenching (Supplementary Fig. 6). Direct switching of a single NPA–ATTO647N molecule between the two states was observed and will be a topic of future research^11^.

Results for Cy5 (Fig. 3e,f) were obtained at higher excitation intensities (4 kW cm^−2^), to allow for a direct comparison with previously published results using different approaches towards intramolecular photostabilization^29^. In agreement with earlier findings, the autocorrelation analysis of Cy5 fluorescent time traces^18^ revealed the presence of two different photophysical processes that were attributed to triplet blinking (11±4 ms) and *cis/trans* isomerization (54±12 μs)^4^,^18^,^26^,^27^. The fluorescence lifetime of Cy5 was found to be 1.65±0.15 ns (compare also Supplementary Fig. 7)^18^,^30^. NPA–Cy5 (**7**) under identical conditions revealed bright and prolonged fluorescence emission with typical observation times of several seconds and, as revealed by the autocorrelation analysis, with negligible triplet-state blinking for ∼75% of the observed emitters (Fig. 3f). The remaining autocorrelation decay revealed the on–off transition due to *cis*/*trans* isomerization, with a lifetime in the order of ∼50–70 μs (see Supplementary Figs 7 and 8), which therefore still restricts the overall achievable count rate for the cyanine fluorophores. In addition, a mono-exponential fit does not fully describe the experimental autocorrelation decay (see the deviations between grey fit and black data in Fig. 3f), suggesting a remaining small triplet population with a lifetime <100 μs. A smaller population of molecules (<25%) showed intensity fluctuations on completely different timescales (Supplementary Fig. 8), which is consistent with earlier reports^18^,^19^, and its origin remains to be explained mechanistically. The fluorescence lifetime of NPA–Cy5 was found to be 1.60±0.15 ns and is similar to the parent fluorophore, indicating that any reduction in the signal brightness of NPA–Cy5 is due to static quenching, that is, transient formation of non-fluorescent complexes between photostabilizer and fluorophore rather than dynamic singlet quenching. These findings are consistent with a subtle blue shift in the Cy5-absorption spectrum when bound to NPA (Supplementary Fig. 2a).

**Figure 4:**
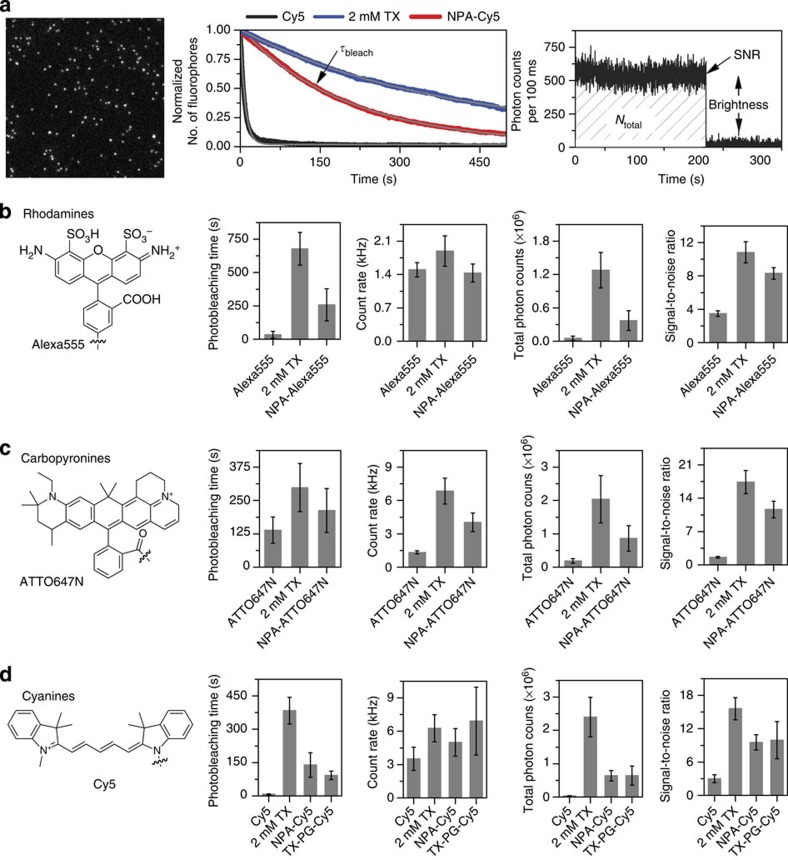
Photophysical characterization of photostabilizer–dye conjugates and their parent fluorophores with single-molecule TIRF microscopy. Data was recorded in aqueous PBS buffer at pH 7.4 in the absence of oxygen under continuous 640 nm excitation with ≈50 W cm^−2^ or 532 nm excitation with ≈20 W cm^−2^. (**a**) Representative image frame (left panel) showing single fluorescent molecules (10 × 10 μm, exemplarily for Cy5). Subsequent images recorded over a period of 500 s showed an exponential decrease in the number of fluorescing molecules with a photobleaching lifetime *τ*_bleach_, as shown for Cy5 (black), Cy5 with 2 mM TX (blue) and NPA–Cy5 (red) (middle panel). The curves shown were obtained by averaging over >5 TIRF movies. (**b**,**c**,**d**) Right panel: chemical structures (left panels) and respective photophysical parameters obtained from background-corrected fluorescence traces for Alexa555 (**b**, rhodamines), ATTO647N (**c**, carbopyronines) and Cy5 (**d**, cyanines). Values and error bars (s.d.) in bar graphs obtained from *N*>500 molecules. For further details of the experimental techniques, data acquisition and analysis, see the Methods section.

To further quantify the improved performance of the photostabilizer–dye conjugates, we used single-molecule TIRF microscopy to determine various parameters with high statistics. Movies with 100 ms integration time were recorded at laser excitation intensities of ≈50–100 W cm^−2^, that is, a significantly lower excitation intensity than in the confocal scanning microscopy experiments, yielding different count rates and SNR ratios. Figure 4a shows a typical example of a camera frame with single Cy5 fluorophores. The number of fluorescent molecules per video frame was determined and the decay in number of molecules over subsequent image frames was fitted with an exponential decay (*y*(*t*)=*C*+*A* × exp(−*t*/*τ*_bleach_)), to obtain the photobleaching lifetime *τ*_bleach_ (Fig. 4a)^18^. Background-corrected single-molecule time traces were extracted and used to determine the fluorescence count rate in kHz (Fig. 4a, brightness), the SNR ratio (Fig. 4a) and the total number of detected photons before photobleaching (Fig. 4a, *N*_total_=brightness × *τ*_bleach_). The mean and s.d. of all values was derived from multiple (*n*≥3) independent experiments. We benchmarked the performance of the photostabilizer–dye conjugates against the antioxidant TX as a solution additive in the deoxygenated imaging buffer (2 mM after 20 min ultraviolet treatment), which is a common standard for photostabilization in single-molecule experiments^10^,^12^.

The data of the non-stabilized rhodamine Alexa555 revealed a brightness of 1.5±0.2 kHz, *N*_total_=5.1±0.4 × 10^4^ and an SNR=3.5±0.3, whereas the photobleaching could be described by a mono-exponential decay with a time constant *τ*_bleach_=34±26 s (Fig. 4b). An improvement for most of the photophysical parameters was found for both adding 2 mM TX or Alexa555-NPA with up to a 20-fold increase in photostability, 25-fold increase in *N*_total_ and a 3-fold increase in SNR, whereas the brightness was found to be comparable.

Similar results were obtained for both the carbopyronine ATTO647N and the cyanine dye Cy5 (Fig. 4c and Supplementary Movie 1). The photobleaching time increased from *τ*_bleach_=138±93 s (ATTO647N) to 212±52 s (NPA–ATTO647N) and 298±45 s (ATTO647N+2 mM TX), and from *τ*_bleach_=7.0±1.5 s (Cy5) to 139±55 s (NPA–Cy5) and 384±60 s (Cy5+2 mM TX). This results in reduced blinking and an increase in the total number of detected photons (*N*_total_) and SNR from *N*_total_=1.9±0.7 × 10^5^ and SNR=1.6±0.2 (ATTO647N) to *N*_total_=8.6±0.4 × 10^5^ and SNR=11.6±1.7 (NPA–ATTO647N), and *N*_total_=2.0±0.7 × 10^6^ and SNR=17.4±2.4 (ATTO647N+2 mM TX), as well as *N*_total_=3.1±0.9 × 10^4^ and SNR=3.0±0.7 (Cy5) to *N*_total_=6.4±1.6 × 10^5^ and SNR=11.6±1.7 (NPA–Cy5), and *N*_total_=2.4±0.6 × 10^6^ and SNR=9.5±1.2 (ATTO647N+2 mM TX), that is, 20- to 50-fold increases.

The investigations in Figs 3 and 4 regarding the photophysical properties of rhodamines, carbopyronines and cyanines with intramolecular photostabilization revealed the following: (i) photobleaching and blinking could be efficiently removed using UAA scaffolding and (ii) all photophysical parameters were improved substantially compared with the parent fluorophore. (iii) Despite showing no decrease in brightness, rhodamines and carbopyronines showed singlet quenching, whereas cyanines show no reduction of the excited state lifetime. (iv) Solution-based healing using 2 mM TX remained more efficient than intramolecular photostabilization.

Despite the slightly lower photostability of compounds with intramolecular photostabilization compared with solution additives such as TX, this approach has unique advantages that compensates for these shortcomings. The UAA approach is thus far the only possible method for photostabilization when organic fluorophores are used under live-cell conditions, in experiments where the addition of a diffusion-based photostabilizer is not tolerated due to its toxicity or when the photostabilizer has an unwanted influence on properties of the system of interest^31^. It is also the only viable option when diffusion-based photostabilization remains ineffective which could be caused by lack of collisions between the photostabilizer and the fluorophore^32^,^33^.

### UAAs as a general scaffold for photostabilizer–dye conjugates

**Figure 5:**
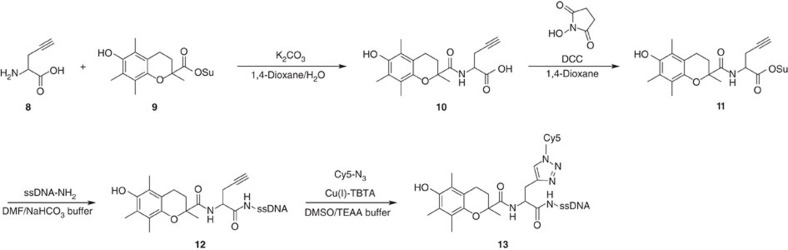
Synthesis route to obtain TX–PG–Cy5 (13) on ssDNA. DCC, *N*-*N*’-dicyclohexylcarbodiimide; Su, succinimide; DMF, dimethylformamide; NaHCO_3_ buffer, 200 mM sodium hydrogen carbonate buffer pH 8.35; TBTA, tris[(1-benzyl-1H-1,2,3-triazol-4-yl)methyl]amine; TEAA buffer, 50 mM triethylammonium acetate buffer, pH 7.0.

The scaffolding strategy presented in Fig. 2 is restricted by the availability of commercial UAAs with different (photostabilizing) residues and does not allow to use custom-made photostabilizers^19^. We hence set out to generalize the approach and to allow linkage of three arbitrary moieties. The amino acid propagylglycine (PG (**8**); Fig. 5) represents a more versatile scaffold that can link three chemical groups via NHS and click chemistry (here, a [3+2]-Huysgens cycloaddition between alkyne and azide derivatives). Hence, PG (**8**) provides the flexibility needed to combine any photostabilizer and fluorophore on a (bio)molecular target, assuming their availability as NHS- or click-reactive derivatives.

In the first synthetic step, racemic **8** was reacted with the NHS-ester derivative of TX (**9**). The resulting adduct **10** was converted into the corresponding NHS-ester derivative **11** and reacted with the 5′-aminoalkyl functionalized oligonucleotide to yield **12**. Finally, the fluorophore Cy5 was bound using a copper-catalysed click reaction^34^ yielding **13**, called TX–PG–Cy5 throughout the manuscript.

TX–PG–Cy5 (**13**) showed a very similar photophysical behaviour compared with NPA–Cy5, that is, removal of blinking and an increased photobleaching lifetime (Fig. 4d) with significant improvement factors (up to 11-fold). However, a higher heterogeneity was observed with ∼60% of the molecules showing fluorescent traces with little intensity fluctuations (shorter bleaching lifetime; Supplementary Fig. 9). The remaining ∼40% exhibited longer observation times with an increased amount of blinking events (Supplementary Fig. 9). A unifying fluorescence lifetime of 1.65±0.15 ns was observed, which is equal to the parent Cy5. Several TX–PG–Cy5 molecules showed a typical behaviour for fluorophores with intramolecular photostabilization, where ‘bleaching’ or irreversible destruction of the stabilizer occurred before photobleaching of the fluorophore. Here the fluorophore drastically changed its emission pattern from stable to a blinking emission pattern that closely resembles the behaviour in the absence of photostabilizer (Supplementary Fig. 9d, bottom row, left panel: stable: 0–8.5 s; blinking: 8.5–10 s).

The PG-based fluorophore system showed to be a versatile scaffold where the photostabilizer and fluorophore can be varied independently of each other. This has the advantage that the fluorophore and photostabilizer can be matched, to obtain maximum photostabilization as was shown above for the fluorophore Cy5 and the photostabilizer TX.

### Direct labelling of biomolecules

The synthesis and photophysical characterization of the photostabilizer–dye conjugates in the previous sections demonstrated the principle improvements in fluorescence emission properties by the UAAs scaffolding approach. Yet, our overall goal is to show the potential of the new fluorophores in biological research. For this, the most important step is labelling biomolecules such as proteins, nucleic acids or antibodies. As shown in ‘UAAs as a general scaffold for photostabilizer–dye conjugates’, this usually requires a complex (multi-step) synthesis process, demanding larger amounts of substances, that is, both fluorophore and biomolecular target. However, the amount of biomolecules is often limited. We hence reduced the chemical steps needed for labelling to a minimum. For this we altered the synthesis strategy of the photostabilizer–dye conjugates, introducing a functionalized group such as a NHS ester or a maleimide, which allow using the fluorophore derivative straightforward for labelling of a biomolecular target via primary amines or cysteine residues (as is done conventionally). NPA was again used as a scaffold for this second generation of photostabilizer–dye conjugates.

**Figure 6 f6:**
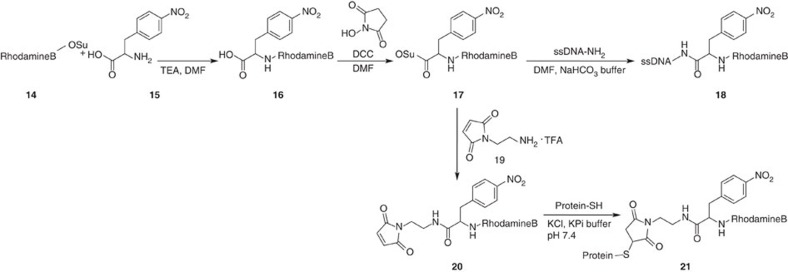
Synthesis of reactive photostabilizer–dye conjugates of RhodamineB for direct labelling of primary amines and thiol residues. The strategy can be extended to other biochemical targets by a varying the linker of molecule 19.

Owing to its ease and cost-effective accessibility, we first used the NHS-ester derivative of the dye RhodamineB (**14**), which was subjected to NPA (**15**) under basic conditions to yield **16** (Fig. 6). Subsequent activation with NHS/*N*-*N*’-dicyclohexylcarbodiimide in dimethylformamide (DMF) gave the NHS-ester derivative of the NPA–RhodamineB conjugate (**17**). In a first step, we (as for the previous fluorophores) targeted this conjugate to ssDNA (**18)**, which was done straightforward from **17** without further purification. The photophysical characteristics of the resulting compound were characterized when immobilized on glass in deoxygenated buffer.

Similar to the fluorophores studied before (see Fig. 3), RhodamineB showed strong blinking in deoxygenated PBS buffer with typical observation times until photobleaching on the timescale of 10–20 s (Supplementary Fig. 10). In contrast, RhodamineB displayed strongly heterogeneous blinking characteristics with very short or longer off times within one trace (Supplementary Fig. 10). Consequently, the autocorrelation function of this fluctuating signal could only be described by a bi-exponential decay with average off times peaking at 7±4 ms and a significant fraction of values >20 ms, deviating from a normal distribution (Supplementary Fig. 10). This indicates the presence of multiple dark states or heterogeneous dye environments. Still the blinking off times were in the same range as determined before for the triplet state of the structurally related rhodamine fluorophores, for example, Alexa555 (22±6 ms, Supplementary Fig. 3) or ATTO565 (6±2 ms)^4^ and are hence considered to be triplet related. Yet, the fluorescence lifetime of RhodamineB was found to be rather homogeneously distributed with an average of 3.1±0.4 ns (Supplementary Fig. 10). Strikingly, NPA–RhodamineB on ssDNA (**18**) showed strongly reduced blinking, an increased photostability resulting in a stable and non-blinking emission pattern with observation times of up to minutes (Supplementary Fig. 11) and an increased brightness (Supplementary Fig. 12).

**Figure 7 f7:**
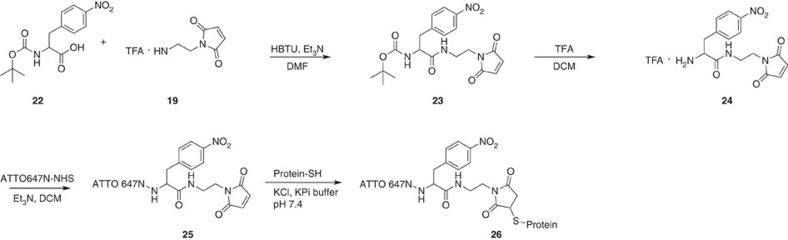
Simplified synthesis of reactive photostabilizer–dye conjugates where only small quantities of fluorophore are available. The resulting NPA–ATTO647N conjugate can be used for direct labelling of thiol residues, for example, in proteins (compound **25**). The strategy can be extended to other biochemical targets by a variation of the linker molecule 19.

Next, we intended to use our NPA-based photostabilizer–dye conjugates for direct labelling of proteins. For this purpose, two different synthesis strategies were developed, accounting for the available quantity of the fluorophore. In the most straightforward case, that is, larger amounts of amine-reactive photostabilizer–dye conjugate are available (for example, >50 mg of compound **17** was available), the NHS ester of the fluorophore (**17**) can be coupled directly with 2-maleimidoethylamine (**19**), to yield a maleimide derivative of, for example, NPA–RhodamineB (**20**) in Fig. 6. The second strategy is also feasible for small quantities of reactive fluorophore species (<10 mg) that could be due to high prices of commercially available precursors or complicated synthesis. ATTO647N is a good example for a fluorophore that is often used in demanding fluorescence applications but is not readily available in large amounts. For these cases we optimized the synthesis route as shown in Fig. 7, to yield a thiol-reactive derivative of ATTO647N containing the photostabilizer NPA (Fig. 7, compound **25**). As shown in ‘Biomolecular FRET study with photostabilizer–dye conjugates’, both maleimide derivatives can covalently bind to recombinant proteins via solvent-exposed cysteine residues (Fig. 6 compound **21** and Fig. 7 compound **26**).

### Biomolecular FRET study with photostabilizer–dye conjugates

**Figure 8:**
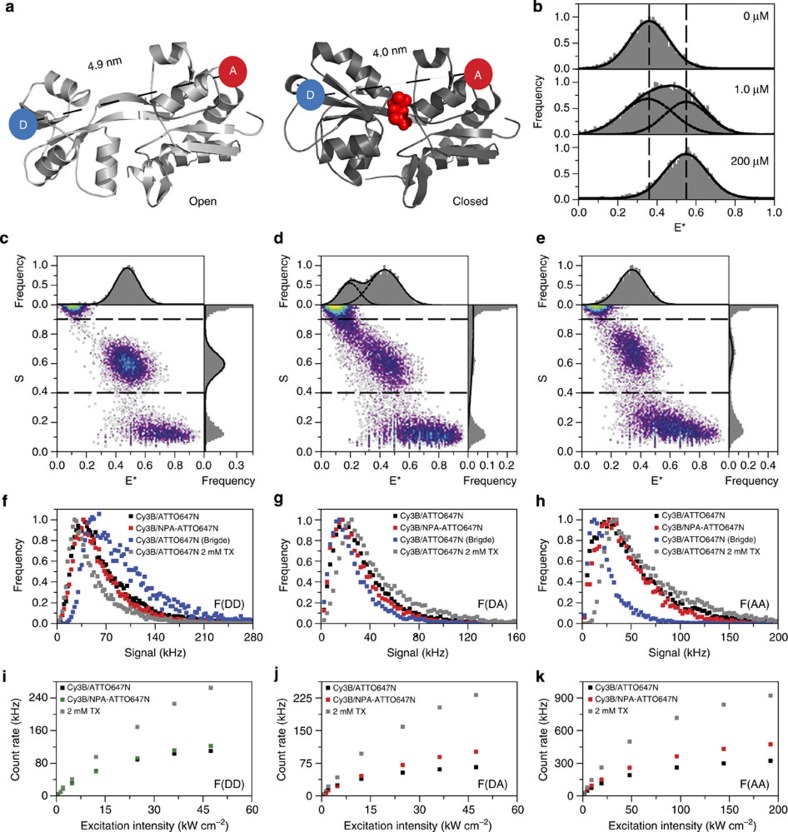
Improving smFRET–ALEX measurements by using NPA-based fluorophore as FRET acceptor on the protein GlnPQ–SBD2. (**a**) Crystal structures of the SBD2 (T369C/S451C) open (left panel, PDB: 4KR5) and closed state (right panel, PDB:4KQP, after binding of the ligand glutamine shown in red) with label positions of donor (D) and acceptor (A). (**b**) Corresponding one-dimensional histograms of E*-values for increasing amounts of ligand glutamine where an increased E*=0.55±0.1 (dashed line, closed conformation) becomes visible (as opposed to the open conformation, E*=0.36±0.1, dashed line). Excitation intensities of 30 kW cm^−2^ at 532 nm and 20 kW cm^−2^ at 640 nm; data evaluated with dual colour burst search and displayed with additional per-bin thresholds of *F*(DD)+*F*(DA)+*F*(AA)>75 and minimal number of counts per bin in ALEX histogram of 3. (**c**,**d**,**e**) 2D histograms of joint pair values of *S* (labelling stoichiometry) and E* (FRET efficiency, that is, interprobe distance) of Cy3B/ATTO647N in the presence (**c**) and absence (**d**) of 2 mM TX and Cy3B/NPA–ATTO647N (**e**) without ligand glutamine. Excitation intensities of 30 kW cm^−2^ at 532 nm and 20 kW cm^−2^ at 640 nm; data evaluated with all photon burst search and displayed with additional per-bin thresholds of *F*(DD)+*F*(DA)+*F*(AA)>100 and minimal number of counts per bin in ALEX histogram of 3. (**f**,**g**,**h**) Histogram of fluorophore brightness values as determined from photon-counting histograms (PCHs) on single-molecule transits of labelled SBD2 diffusing through the observation volume, comparison of donor brightness *F*(DD) (**f**), acceptor brightness when excited via FRET, *F*(DA) (**g**) and acceptor brightness via direct red excitation *F*(AA) (**h**). Excitation intensities of 30 kW cm^−2^ at 532 nm and 20 kW cm^−2^ at 640 nm. (**i**,**j**,**k**) Dependence of the mean count rate of *F*(DD), *F*(DA) and *F*(AA) of the different samples for increasing excitation intensity, respectively.

To show the benefits of intramolecular photostabilization in fluorescence applications with proteins, we studied the substrate-binding domain 2 (SBD2) of the Lactococcus lactis ABC transporter GlnPQ^35^. Using smFRET and alternating laser excitation (ALEX) spectroscopy, the conformational states of the protein were monitored (Fig. 8a, open unliganded and closed liganded). The structural rearrangements of SBD2 on ligand binding causes a change of ∼0.9 nm regarding the distance between two selected amino acids in the protein^35^. As the FRET donor and acceptor are attached via maleimide chemistry at these positions in the protein (mutant of SBD2: T369C/S451C), the transfer efficiency E* reports on the conformational state of the protein. As described previously^35^, SBD2 was labelled stochastically using appropriate mixtures of donor and acceptor fluorophores (details see Methods). To understand the effects of intramolecular photostabilization in FRET-based assays, we used different fluorophore combinations: Cy3B or RhodamineB as donor fluorophores and ATTO647N as the acceptor. In experiments described below, either the donor (RhodamineB) or the acceptor (ATTO647N) was stabilized via covalent linkage to NPA (synthesis see Figs 6 and 7).

We used smFRET and ALEX spectroscopy, as described in refs 33, 35, 36, to investigate the photophysical properties of the fluorophores when bound covalently to the protein and the biomolecular function of SBD2. ALEX is a valuable tool for both purposes, as it allows to distinguish the desired protein molecules containing both fluorophores, thus monitoring protein conformation (donor–acceptor species; Fig. 8b, 0.9<*S*<0.4), from those labelled with only one species not providing distance information (donor only, *S*>0.9; acceptor only, *S*<0.4). Although *S* relates to the relative fluorophore brightness and labelling stoichiometry, E* indicates the FRET efficiency and thus distance between the donor and acceptor (with larger values indicating small donor–acceptor distances), which is the final read out of the protein conformation. In addition, ALEX reveals photophysical artefacts such as blinking of donor or acceptor in the form of bridges between the three different subpopulations^37^. For such measurements, the fluorescence emission of the donor *F*(DD) under green excitation, that of the acceptor *F*(DA) when excited via FRET from the donor and that of the acceptor via direct red excitation light *F*(AA) was determined (see Methods and refs 33, 35, 36). In our experiments, individual biomolecules were studied for short time periods of a few milliseconds, while diffusing through the excitation volume of a confocal microscope. The challenge of such an experiment is to acquire intense fluorescent bursts during the short observation time under the high excitation intensities of >10 kW cm^−2^.

The combination of Cy3B and ATTO647N is known for excellent photophysical performance resulting in fast data acquisition and superior histogram quality in smFRET with little bleaching artefacts and narrow distributions (Fig. 8c). For apo-SBD2 we find mean E* and *S* of 0.48±0.08 and 0.59±0.07, respectively, with these labels^35^. These values are in good agreement with our published work and the mean E* correlates with the expected interprobe distance of ∼4.9 nm derived from the crystal structure.

Results of such quality are, however, only available when using 2 mM TX as a photostabilizer in solution, seen from comparison of Fig. 8c,d. Here we show data of apo-SBD2 (labelled with Cy3B/ATTO647N) in the presence and absence of TX. In agreement with Kong *et al*.^37^, the high excitation intensities used in our experiments promote acceptor signal fluctuations, that is, blinking and/or bleaching. Cy3B and ATTO647N can hence be seen as a FRET couple where the acceptor photostability is limiting. This appears as a prominent bridge between the donor-only and donor-acceptor population (Fig. 8d), altering both E*/*S*-values substantially. Closer inspection of the histogram reveals that a significant portion of the molecules show these unwanted photophysical effects. Under these conditions, neither mean E* nor correction factors for accurate FRET determination are directly accessible. Besides the complete loss of information, the overall acquisition time has also increased in the absence of photostabilizer to obtain sufficient statistics from the relevant donor–acceptor species. It should be noted that such photophysical artefacts of the acceptor (Fig. 8d) are extremely problematic for data interpretation, as they suggest the existence of (non-biological) species in between the donor only and the actual FRET species (see 1*D*−E* in Fig. 8d that can only be fitted by the sum of two Gaussians with E*=0.19±0.07 and 0.43±0.1). Furthermore, it remains challenging to identify those when performing smFRET with green excitation in continuous-wave mode.

Strikingly, the bridge population can be removed by sole photostabilization of the acceptor fluorophore via scaffolding of ATTO647N to NPA. The ALEX data of apo-SBD2 with Cy3B/NPA–ATTO647N is shown in Fig. 8e. Here the bridge between the donor–acceptor and donor-only population is fully removed without the addition of stabilizer to the solution (Fig. 8d versus Fig. 8e). The resulting mean E*/*S* values are changed compared to apo-SBD2 with Cy3B/ATTO647N (2 mM TX in solution, Fig. 8b) accounting for decreased acceptor brightness; mean E* is now found at 0.34±0.09 and mean *S* at 0.67±0.08 (Fig. 8e). These differences are, however, expected considering the results from ATTO647N on DNA, where a decrease in the overall brightness is observed on conjugation to NPA (Fig. 4c). It should be mentioned that differences in the donor–acceptor population relative to donor and acceptor only comparing the samples Cy3B/ATTO647N and Cy3B/NPA–ATTO647N are not solely due to photophysics but also due to different labelling ratios of the protein.

Next, we used Cy3B/NPA–ATTO647N to study the biomolecular function of SBD2 (Fig. 8b). On addition of the ligand glutamine, the mean E* changes from 0.36±0.1 (open, interprobe distance of ∼4.9 nm) to 0.55±0.1 (closed, with a decreased interprobe distance of ∼4.0 nm). A concentration of 200 μM saturates ligand binding and therefore results in a 100% population of the closed state (Fig. 8b). A ligand concentration of 1 μM, which is close to the *K*_d_-value of the protein^38^, consequently results in a mix of open and closed states (Fig. 8b).

As fluorophore brightness and the resulting photon budget ultimately determine the quality of the final histograms including the necessary measurement time, we quantitatively analysed Cy3B/ATTO647N and Cy3B/NPA–ATTO647N by means of photon-counting histograms. Figure 8f,g,h shows the three relevant photon streams used to determine E* and *S* of Cy3B/ATTO647N (donor–acceptor: 0.9>*S*>0.4; bridge: 0.9>*S*>0.7) and Cy3B/NPA–ATTO647N (donor–acceptor: 0.9>*S*>0.4). *F*(DD) shows that the strongest donor quenching and hence the most efficient FRET is found for the addition of TX to Cy3B/ATTO647N, whereas molecules in the bridge (Cy3B/ATTO647N, no TX) show inefficient donor quenching due to a non-functional acceptor (Fig. 8f). Cy3B/NPA–ATTO647N and healthy molecules from the Cy3B/ATTO647N population with no TX show a similar performance of the acceptor-based donor quenching. *F*(DA) correlates directly with quenching in *F*(DD) as seen in Fig. 8g. Again, the best performance is observed from Cy3B/ATTO647N in the presence of TX, while molecules in the bridge show the lowest counts. A striking difference between the bridge and all other conditions is seen in *F*(AA), that is, direct excitation of the acceptor (Fig. 8h). The overall comparison suggest that NPA-based acceptor stabilization is sufficient to remove photophysical artefacts and hence make the smFRET useful for biomolecular studies. Further optimization of the data quality could, however, be obtained by additional donor stabilization of, for example, Cy3B in this case. This interpretation is further supported by excitation intensity-dependent count rates of Cy3B/ATTO647N and Cy3B/NPA–ATTO647N (Fig. 8i,j,k). NPA-based acceptor stabilization improves the saturation characteristics in all three channels but the addition of TX to the solution (resulting in stabilization of both donor and acceptor at the same time) remains superior in terms of achievable count rates.

To study the donor dependence in more detail, we repeated the above described experiments using RhodamineB/ATTO647N and NPA–RhodamineB/ATTO647N. Here, the photostability of the donor is the limiting factor for smFRET data quality. For RhodamineB/ATTO647N we find prominent donor blinking in the absence of photostabilizer (Supplementary Fig. 13). The bridge between the donor–acceptor and acceptor-only population can be removed by addition of TX in solution (Supplementary Fig. 13) or conjugation of RhodamineB to NPA (Supplementary Fig. 13). The overall magnitude of the observed effects is lower than for the case of acceptor bleaching/blinking in Fig. 8. Photon-counting histograms and the intensity dependence show a similar trend as before, that is, correlation between *F*(DD) and *F*(DA) with the wish to increase donor photostability as much as possible. Our data makes clear that NPA–RhodamineB can be used at significantly higher excitation intensities than RhodamineB, as triplet-state population was minimized and would allow for faster and better data acquisition (Supplementary Fig. 13).

Our results show that the simple addition of the NPA unit to the acceptor fluorophore is sufficient to gather reliable results from solution-based smFRET (Fig. 8). Although the addition of a stabilizer on the donor fluorophore can increase the overall available photon budget (which should always be maximized), the acceptor strictly requires a solution-based or covalently linked photostabilizer. We consequently suggest to stabilize the acceptor as a minimum and (if possible) also to stabilize the donor to maximize available count rates and hence data quality. We finally note that all smFRET results of SBD2 with the different pairs of fluorophores shown here are in good agreement with biochemical data from isothermal calorimetry and with our previous results, supporting our hypothesis of an induced-fit-type mechanism in GlnPQ^35^. In addition, it confirms successful labelling of the protein with the custom-made NPA derivative, and that the results from the established FRET assay are indeed independent of the fluorophore pair that is used to monitor the protein conformation^35^. It is noteworthy that spectrally uncorrected apparent FRET is reported here accounting for absolute differences in mean E*-values from varying Förster radius *R*_0_ of the fluorophore pairs used or variations in the setup alignment^35^.

### Photostabilizer–dye conjugates in super-resolution microscopy

**Figure 9:**
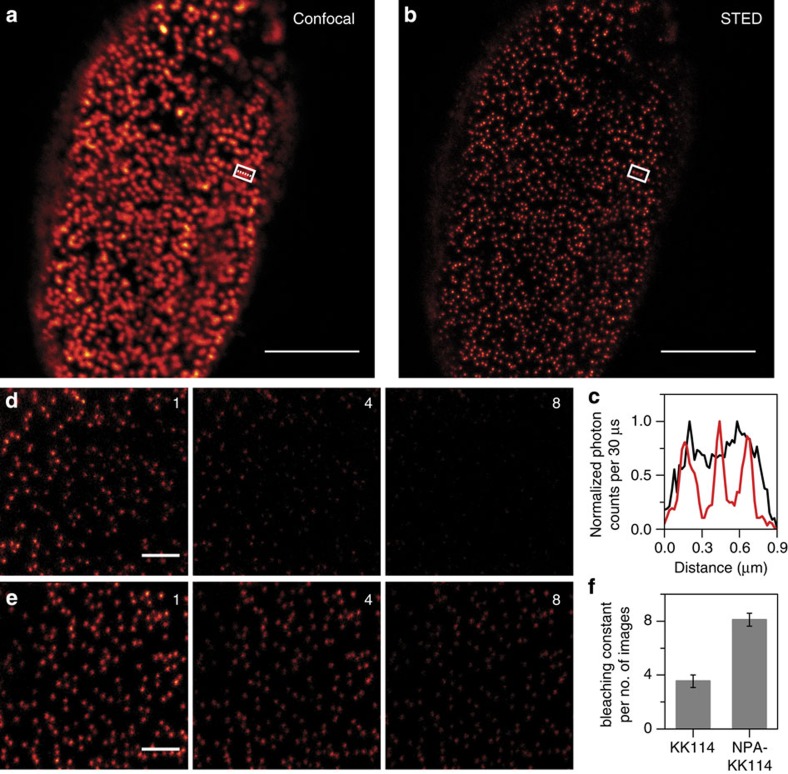
Use of photostabilizer–dye conjugates in confocal and super-resolution STED microscopy of immunolabelled cells. Data from NPCs in fixed mammalian PtK2 cells immunolabelled with KK114 and NPA–KK114 Confocal (**a**) and STED (**b**) images of a representative cell stained with NPA–KK114 (scale bars, 5 μm). (**c**) Normalized intensity profile along the dashed line in the white boxes marked in the images of **a**,**b**, exemplifying how neighbouring NPCs can be much better resolved in the STED (red) compared with the confocal (black) recordings. (**d**–**f**) Repeated scanning of the same area of the cells in the STED mode indicates reduced photobleaching in the case of NPA–KK114 compared with KK114: images 1, 4 and 8 for KK114 (**d**) and NPA–KK114 (**e**) (scale bars, 1 μm), and (**f**) bleaching constant from an exponential fit in terms of number of images for KK114 and NPA–KK114 (total number of subsequent images recorded=30, *n*=4).

A common tool in cellular far-field fluorescence microscopy is the use of immunofluorescence, where the majority of cells are fixed and individual structures or proteins are tagged using specific primary antibodies and fluorophore-labelled secondary antibodies. In recent years, pioneering developments in far-field fluorescence microscopy, so-called super-resolution microscopes, have revolutionized cellular imaging, allowing the visualization of biological structures with nanometre resolution, that is, beyond the physical diffraction limit that thus far prevented to resolve structures with a precision better than ∼250 nm^2^. Given the importance of photostability and brightness in such techniques, we investigated the potential of fluorophores with intramolecular photostabilization for immunofluorescence and specifically super-resolution imaging. For this, we have used the NHS ester of the dye KK114 (ref. 39) and its derivative NPA–KK114 (Supplementary Fig. 14) to directly tag secondary antibodies using standard procedures. These antibodies were used to specifically immunolabel nuclear pore complexes (NPCs) in fixed mammalian PtK2 cells. Figure 9a and Supplementary Fig. 15 depict that we could successfully apply NPA–KK114 to visualize the spatial distribution of these NPCs as shown in the representative confocal scanning images. However, it also becomes obvious that these complexes appear as rather large (>250 nm), blurred spots due to the diffraction limit. To increase resolution, we employed super-resolution STED microscopy^3^,^40^, which in its most common application adds a STED laser to the confocal microscope that features a focal intensity distribution with a central zero, to allow imaging with subdiffraction spatial resolution. Figure 9b and Supplementary Fig. 15 clearly show the succesfull implementation of NPA–KK114 in STED microscopy. The NPCs were much better resolved and appeared as much smaller spots (85–90 nm, which is a reasonable value considering the use of primary and secondary antibodies). When compared with conventional KK114, the NPA–KK114 showed an increased photostability under STED conditions. Repeated scanning of the same area of the cell revealed that fading of fluorescence was reduced for NPA–KK114, while brightness was only subtly increased (Fig. 9d–f, Supplementary Fig. 15 and Supplementary Movie 2). The improvement in photobleaching resistance under STED conditions via an intramolecular mechanism highlights future directions towards improved dynamic STED imaging without the need of adding (potential toxic) chemical compounds^41^.

## Discussion

Our results show that effective intramolecular photostabilization and removal of (triplet induced) dark states can be achieved for NPA- and PG-based fluorophore derivatives with similar or better photostabilization effects as previously introduced photostabilizer–dye conjugates. This shows that the UAA scaffold is a simple and useful tool to covalently link fluorophore, photostabilizer and biomolecular target in a modular manner. The presented approach has many advantages over existing strategies, as it is cost effective, requires low amounts of material, uses only well-known chemical transformations such as amide bond formation or click chemistry, requires standard purification procedures and has the potential to address various important biomolecular targets.

Moreover, our conjugation strategy opens the possibility to apply intramolecular photostabilization to fluorophore classes that could not be studied before. This possibility broadens our understanding of intramolecular photostabilization and might provide a basis for further improvement of the photostabilizer–dye conjugates by studying the effects of fluorophore properties such as charge, redox potential or absorption/emission wavelength. For certain types of fluorophores, for example, oxazines and perylenes, conjugation to NPA is expected to show effects different from those described here considering their interaction with antioxidants^4^,^24^,^25^, DNA bases and amino acids such as tryptophane^42^,^43^,^44^. Intramolecular quenching of Alexa fluorophores observed with natural amino acids^45^ even suggests that labelling of proteins at strategic locations, for example, in the vicinity of aromatic amino acids, could be used for fluorophore stabilization without the use of UAAs, provided that a suitable combination of fluorophore and amino acids can be found.

Intramolecular photostabilization remains, however, less effective compared to the use of diffusion-based photostabilization (see Fig. 4) as reported before^18^. This shortcoming of intramolecular photostabilization is compensated by the fact that it is often the only viable option for applications such as live-cell imaging, for assays where the addition of photostabilizer remains ineffective or is not tolerated by the biological system. We are convinced that future work—also facilitated by the presented synthesis strategy—will allow solving this problem. We hypothesize that the properties of photostabilizer–dye conjugates can be optimized by parameters such as the linking geometry between fluorophores and photostabilizer. This is supported by published data of Cy5 derivatives, where we linked the aromatic nitro group to the fluorophore in a different way^19^, compared with the compounds shown in this study (compound **7**). These architectures show a significantly higher photostability using basically the same photostabilizer moiety. We are in the process of varying the linker length of NPA-based fluorophore derivatives, to optimize the effects of intramolecular photostabilization and to become fully competitive with solution additives as shown in ref. 19.

We finally suggest that UAA scaffolding could be extended beyond photostabilization, to provide a general framework for the manipulation of fluorophore properties. Potentially interesting UAAs feature antioxidants, triplet sensitizers, photoswitchable molecules to induce blinking for localization-based super-resolution microscopy^2^,^3^,^4^,^46^,^47^, alter water solubility^48^ or the affinity to membranes and residues of natural amino acids such as thiols (see Lui *et al*.^23^ for an overview of UAA residues). UAAs with ‘clickable’ functionalities could be used to link the fluorophore to a biomolecular target and an affinity tag (strep-/his-tag) to simplify purification of labelled protein species.

In summary, we introduced a versatile and simple design concept to synthesize photostabilizer–dye conjugates on a specific target using UAAs. The strategy is based on a straightforward and modular synthesis route using amide bond formation and click chemistry with commercially available starting materials. The UAAs NPA and PG were used as a scaffold to link rhodamine, carbopyronine and cyanine fluorophores covalently to a photostabilizer on a biomolecular target, such as DNA, antibodies and proteins in our case study. We are convinced, however, that other targets (RNA, affinity tags and so on) can also be labelled via similar means, while maintaining the positive effects of photostabilization on these targets. All studied compounds show a considerable increase in photostability and a suppression of triplet-based blinking compared to the non-stabilized parent fluorophore. With this, we are the first to test intramolecular photostabilization for various classes of organic fluorophores and show that intramolecular triplet-state quenching is a generally applicable method. The approach allows labelling of biomolecules in a single step with the same effort as for commercially available reactive fluorophore derivatives. Finally, photostabilizer–dye conjugates were used in two state-of-the-art applications, that is, the study of conformational changes in proteins via smFRET and super-resolution imaging using STED microscopy. We are convinced that the presented strategy will stimulate broader use of intramolecular photostabilization and help to emerge this strategy to the new gold standard for photostabilization.

## Methods

### Synthesis of photostabilizer–dye conjugates and precursors

All reagents were purchased from commercial suppliers and used without further purification, unless stated otherwise. Cy5 fluorophores were obtained from Lumiprobe (Germany), *Alexa555-NHS* was obtained from Life Technologies (USA) and ATTO647N-NHS from ATTOTEC (Germany). Synthetic oligomers (NH_2_-C6-5′-TAA TAT TCG ATT CCT TAC ACT TAT ATT GCA TAG CTA TAC G-3′) were received in HPLC-purified quality from IBA or Eurofins (Germany). TX–NHS **9** was synthesized following a published procedure^49^. A Varian 400 (400 and 100 MHz) and Varian 500 MHz were used to record ^1^H-NMR and ^13^C-NMR spectra. Chemical shifts (*δ*) are denoted in p.p.m. using residual solvent peaks as internal standard (*δ*_H_=7.26 and *δ*_C_=77.0 for CDCl_3_; *δ*_H_=3.31, 4.78 and *δ*_C_=49.15 for CD_3_OD). High-resolution mass spectra were recorded on an Orbitrap XL (Thermo Fisher Scientific; ESI pos. or neg. mode). Liquid chromatography–mass spectra were recorded on Waters Acquity H-class UPLC equipped with TUV detector and a Xevo G2 TOF mass detector from Waters Chromatography BV. Flash chromatography was performed using a Grace Reveleris Flash System (40 μm silica column).

*Purification of functionalized oligonucleotides*. The functionalized oligonucleotides were purified on an Illustra NAP 5 column loaded with Sephadex G-25 DNA Grade material obtained from GE Healthcare. Illustra NAP 5 columns were equilibrated with 3 × 5 ml of eluent before use (50 mM triethylammonium acetate (TEAA) buffer, pH 7.0, or water for Fmoc-protected oligonucleotide **3** and **12**). After the reaction, the oligonucleotide sample was diluted with eluent to a final volume of 0.5 ml and added to the column. After the sample was loaded, 1 mL of eluent was added and the purified oligonucleotide was collected in one portion. The oligonucleotide solution was lyophilized (Christ Alpha 2–4 LD plus freeze dyer) directly after collection.

*Isolation and characterization of functionalized oligonucleotides*. Reversed-phase HPLC (rp-HPLC) analysis and preparative purifications (isolation) was performed on a Shimadzu LC-10AD VP machine equipped with Waters Xterra MS C18 column (3.0 × 150 mm, particle size 3.5 μm) and Waters Xterra MS C18 prep column (7.8 × 150 mm, particle size 10 μm) using a gradient of acetonitrile/TEAA buffer (50 mM, pH 7.0). Gradient 1: 05/95, 0–10 min, to 65/35 at 60 min, to 75/25 at 65 min, to 05/95 at 75 min for 15 min. Gradient 2: 05/95, 0–10 min, to 35/65 at 60 min, to 65/35 at 65 min, to 05/95 at 70 min for 20 min. Flow 0.5 ml min^−1^ analytical run or 1.0 ml min^−1^ preparative run. The DNA was isolated by collecting the major peak of interest (see Supplementary Fig. 1). The resulting compounds were characterized by ultraviolet–visible absorption spectroscopy (Supplementary Fig. 2a) and MALDI-TOF mass spectrometry (Supplementary Fig. 2b). Spectra were recorded on an ABI Voyager DE-PRO MALDI-TOF (delayed extraction reflector) Biospectrometry Workstation mass spectrometer.

*Synthesis of ssDNA-NPA-Alexa555/ATTO647N/Cy5*. Step 1: the lyophilized ssDNA NH_2_ was resuspended in MilliQ water and adjusted to 80 μM in 0.2 M NaHCO_3_ buffer, pH 8.35. To 100 μl of this solution, the same volume of a 20 mg ml^−1^ solution of **2** in DMF was added and the mixture was vortexed thoroughly. If necessary, additional DMF was added in 10 μl portions, to obtain a clear solution. After the reaction at room temperature overnight, **3** was purified on Illustra NAP 5 column (*vide supra*, MilliQ water was used as eluent for purification on Illustra NAP 5 columns, to prevent partial deprotection of the Fmoc group), and isolated by preparative rp-HPLC (Supplementary Fig. 2, gradient 1, *vide supra*), to yield ssDNA NPA–Fmoc **3**. Step 2: Fmoc deprotection of **3** was performed as follows. The HPLC-purified and lyophilized oligonucleotide was resuspended in 50 μl of 50 mM TEAA buffer, pH 7.0. Deprotection was achieved by addition of 40 μl DMF and 10 μl piperidine. The mixture was vortexed and allowed to react for 2 h at room temperature. The deprotected oligonucleotide was purified on Illustra NAP 5 column (*vide supra*) and lyophilized to yield **4**. Step 3: coupling with fluorophore NHS was achieved as follows. Cy5–NHS as obtained from Lumiprobe, *Alexa555–NHS* was obtained from Life Technologies and ATTO647N–NHS from ATTOTEC; all samples were evenly distributed into ∼300 nmol portions inside a glove box. Each portion was sealed with Parafilm and kept in the dark at −18 °C. Lyophilized **4** (∼2 nmol) was resuspended into 50 μl of 0.2 M NaHCO_3_ buffer, pH 8.35. To this solution, one portion of fluorophore NHS dissolved in 10 μl of dimethyl sulfoxide (DMSO) was added and the mixture was vortexed thoroughly. After incubation overnight the oligonucleotide was purified on Illustra NAP 5 column (*vide supra*) and isolated by preparative rp-HPLC (gradient 1, *vide supra*) to yield **5**, **6** and **7** (see Fig. 2 and Supplementary Fig. 1).

*Synthesis of ssDNA TX–PG–Cy5 (**13**)*. Step 1: the lyophilized ssDNA was resuspended in MilliQ water and adjusted to 80 μM in 0.2 M NaHCO_3_ buffer, pH 8.35. To 100 μl of this solution, the same volume of a 20 mg ml^−1^ solution of **11** in DMF was added and the mixture was vortexed thoroughly. If necessary, additional DMF was added in 10 μl portions, to obtain a clear solution. After the reaction at room temperature overnight, the oligonucleotide was purified on Illustra NAP 5 column and isolated by preparative rp-HPLC (Supplementary Fig. 1, gradient 2, *vide supra*) to yield **12**. Step 2: coupling with Cy5–N_3_ was achieved following a modified manufacturer protocol (Lumiprobe). After every addition step the mixture was vortexed briefly. The HPLC-purified and -lyophilized oligonucleotide **12** (∼2 nmol) was resuspended in 30 μl of 2 M TEAA buffer, pH 7.0 and added into a 0.5ml tube. To this solution, 8 μl MilliQ water and 7 μl of DMSO were added. A 15-nmol portion of Cy5–N_3_ was dissolved in 40 μl of DMSO and added. To this mixture, 10 μl of 5 mM stock solution of ascorbic acid in MilliQ water was added. The solution was degassed by a stream of N_2_ for 30 s and finally 5 μl of a 10-mM stock solution of Cu(II)-tris[(1-benzyl-1H-1,2,3-triazol-4-yl)methyl]amine in 55% DMSO/MilliQ water was added. The vial was flushed with N_2_, closed and sealed with Parafilm. After incubation overnight the oligonucleotide was purified on Illustra NAP 5 column (gradient 2, *vide supra*) and isolated by preparative rp-HPLC (*vide supra*) to yield **13** (Supplementary Fig. 1).

*Synthesis of ssDNA Alexa555/ATTO647N*. Step 1: *Alexa555–NHS* was obtained from Life Technologies and ATTO647N–NHS from ATTOTEC; all were evenly distributed into ∼300 nmol portions inside a glove box. Each portion was sealed with Parafilm and kept in the dark at −18 °C. ssDNA NH_2_ (∼2 nmol) was resuspended into 50 μl of 0.2 M NaHCO_3_ buffer, pH 8.35. To this solution, one portion of *Alexa555–NHS/ATTO647N–NHS* dissolved in 10 μl of DMSO was added and the mixture was vortexed thoroughly. In case of RhodamineB, an NHS crude mixture in 100 μl of DMF was added to ssDNA NH_2_ and the mixture was vortexed thoroughly. After incubation overnight the oligonucleotide was purified with an Illustra NAP 5 column (*vide supra*) and isolated by preparative rp-HPLC (gradient 1, *vide supra*).

*Synthesis of ssDNA NPA–RhodamineB*. Step 1: the lyophilized ssDNA-NH_2_ was resuspended in MilliQ water and adjusted to 20 μM in 0.2 M NaHCO_3_ buffer, pH 8.35. To this solution, RhoB–NPA–NHS (**20**) in 100 μl of DMF was added and the mixture was vortexed thoroughly. After incubation overnight the oligonucleotide was purified on Illustra NAP 5 column (*vide supra*) and isolated by preparative rp-HPLC (gradient 1, *vide supra*) to yield **18** (see Fig. 6). The yield was found to be 25% for coupling NPA–RhodamineB–NHS to ssDNA.

*Synthesis of ssDNA RhodamineB*. Step 1: the lyophilized ssDNA–NH_2_ was resuspended in MilliQ water and adjusted to 20 μM in 0.2 M NaHCO_3_ buffer, pH 8.35. To this solution, RhodamineB–NHS in 100 μl of DMF was added and the mixture was vortexed thoroughly. After incubation overnight the oligonucleotide was purified on Illustra NAP 5 column (*vide supra*) and isolated by preparative rp-HPLC (gradient 1, *vide supra*) (see Supplementary Fig. 1).

*Labelling of GlnPQ–SBD2*. SBD2 cysteine-containing derivative was obtained as described in ref. 35 and stored at −20 °C in 100-μl aliquots of 20–40 mg ml^−1^ in 50 mM KPi, pH 7.4, 50 mM KCl and 50% glycerol plus 1 mM dithiothreitol (DTT). Stochastic labelling with maleimide derivatives of donor and acceptor fluorophores was carried out on ∼5 nmol of protein; SBD_2_ derivatives were labelled with RhodamineB–/NPA–RhodamineB–maleimde (donor) and KK114–maleimide (acceptor) in a ratio of protein:donor:acceptor of 1:6:8. Briefly, purified proteins were treated with DTT (10 mM, 30 min), to fully reduce oxidized cysteines. After dilution of the protein sample to a DTT concentration of 1 mM, the reduced protein was bound to a Ni^2+^-Sepharose resin (GE Healthcare) and washed with ten column volumes of 50 mM KPi, pH 7.4, 150 mM KCl and 5% glycerol (buffer B). Simultaneously, the applied fluorophore stocks (50 nmol (s)) dissolved in 5 μl of anhydrous DMSO were added at appropriate amounts to buffer B and immediately applied to the protein bound to the Ni^2+^-Sepharose resin (keeping the final DMSO concentration below 1%). The resin was incubated overnight and kept at 4 °C (under mild agitation). After labelling, unreacted dye was removed by sequential washing with ten column volumes of buffer B, and this was followed by 100 column volumes of 50 mM KPi, pH 7.4, 1 M KCl and 50% glycerol. The protein was eluted in 1 ml of 50 mM KPi, pH 7.4, 150 mM KCl, 5% glycerol and 500 mM imidazole, and was analyzed on a Superdex-200 column (GE Healthcare) equilibrated with 50 mM KPi, pH 7.4, and 200 mM KCl.

### Microscopy and sample preparation

*Sample preparation and surface immobilization of oligonucleotides*. Immobilization and investigation of single fluorophores was achieved using a dsDNA scaffold comprising two 40-mer oligonucleotides, that is, ssDNA fluorophore and ssDNA biotin. Sequences of both oligomers were adapted from refs 12, 24, 25. As a non-stabilized control we used ssDNA fluorophore (Cy5-C6-5′-TAA TAT TCG ATT CCT TAC ACT TAT ATT GCA TAG CTA TAC G-3′; as received from Eurofins and IBA). Single immobilized fluorophore molecules were studied in Lab-Tek eight-well-chambered cover slides (Nunc/VWR, The Netherlands) with a volume of 750 μl as described in ref. 11. After cleaning with 0.1% hydrofluoric acid (HF) and washing with PBS buffer (one PBS tablet was dissolved in deionized water yielding a 10mM phosphate buffer with 2.7 mM potassium chloride and 137 mM sodium chloride at pH 7.4; Sigma Aldrich, The Netherlands), each chamber was incubated with a mixture of 5 mg per 800 ml BSA and 1 mg per 800 ml BSA/biotin (Sigma Aldrich) at 4 °C in PBS buffer overnight. After rinsing with PBS buffer, a 0.2-mg ml^−1^ solution of streptavidin was incubated for 10 min and subsequently rinsed with PBS buffer.

The immobilization of dsDNA was achieved via a biotin–streptavidin interaction using pre-annealed dsDNA with the aim to observe single emitters for prolonged time periods and to guaranty free rotation of fluorophores. For this, 5–50 μl of a 1μM solution of ssDNA fluorophore, ssDNA-NPA-fluorophore or ssDNA-TX-PG-Cy5 was mixed with the complementary ssDNA-biotin at the same concentration (Biotin-5′-CGT ATA GCT ATG CAA TAT AAG TGT AAG GAA TCG AAT ATT A-3′, used as received from IBA). The respective mixtures of two oligomers were heated to 98 °C for 4 min and cooled down to 4 °C with a rate of 1 °C min^−1^ in annealing buffer (500 mM sodium chloride, 20 mM TRIS-HCL and 1 mM EDTA at pH 8). The treated LabTek coverslides were incubated with a 50–100 pM solution of pre-annealed dsDNA for 1–2 min, leading to a typical surface coverage of fluorophore-labelled dsDNA as shown in Figs 3 and 4, [Fig f6], [Fig f7] and Supplementary Figs 3–11.

All single-molecule experiments were carried out at room temperature (22±1 °C). Oxygen was removed from the buffer system using an oxygen-scavenging system (PBS, pH 7.4, containing 10% (wt/vol) glucose and 10% (vol/vol) glycerine, 50 μg ml^−1^ glucose oxidase, 100–200 μg ml^−1^ catalase and 0.1 mM Tris(2-carboxyethyl)phosphine hydrochloride (TCEP)). As shown before, such low concentrations of the reducer TCEP has no noticeable effect on the photophysics of organic fluorophores^25^,^50^ and hence do not convolute with effects from intramolecular stabilization. Glucose oxidase catalase^11^,^29^ was used instead of a combination of protocatechuic acid and protocatechuate-3,4-dioxygenase^29^, to avoid convolution of inter- and intramolecular photostabilization with protocatechuic acid^51^. When adding TX to the imaging buffer (PBS with 2 mM TX), the latter was irradiated with ultraviolet light for 30 min before adding 10% (wt/vol) glucose and 10% (vol/vol) glycerine, 50 μg mL^−1^ glucose oxidase, 100–200 μg mL^−1^ catalase and 0.1 mM TCEP.

*Confocal scanning microscopy and data analysis*. A custom-built confocal microscope, described in ref. 18, was used to study fluorescence properties of organic fluorophores on the level of single molecules. Briefly, excitation was achieved with a spectrally filtered laser beam of a pulsed supercontinuum source (SuperK Extreme, NKT Photonics, Denmark) with an acousto-optical tunable filter (AOTFnc-VIS, EQ Photonics, Germany), leading to ≈2 nm broad excitation pulses centred at 640nm or 532 nm. The spatially filtered beam was coupled into an oil-immersion objective (× 60, numerical aperture (NA) 1.35, UPLSAPO 60XO mounted on an IX71 microscope body, both from Olympus, Germany) by a dichroic beam splitter (zt532/642rpc, AHF Analysentechnik, Tuebingen, Germany). Surface scanning was performed using a XYZ-piezo stage with 100 × 100 × 20 μm range (P-517-3CD with E-725.3CDA, Physik Instrumente, Germany). Fluorescence was collected by the same objective, focused onto a 50-μm pinhole and detected by two avalanche photodiodes (*τ*-spad, <50 dark counts per second, Picoquant, Germany) with appropriate spectral filtering (green: HC582/75, red: ET700/75 AHF, both from Analysentechnik). The detector signal was registered using a Hydra Harp 400 ps event timer and a module for time-correlated single-photon counting (both from Picoquant). The data were evaluated using custom-made LabVIEW software^24^,^25^. Blinking kinetics were extracted from fluorescent time traces in the form of ON and OFF times according to established procedures^24^. Fluorescence lifetimes were determined using time-correlated single-photon counting as described before^18^.

*TIRF microscopy including data analysis*. Widefield fluorescence imaging was conducted on an inverted microscope (Olympus IX-71 with UPlanSApo × 100, NA 1.49, Olympus, Germany) in objective type TIRF) configuration as described before^18^. Images were collected with a back-illuminated electron multiplying charge-coupled device camera (512 × 512 pixel, C9100-13 (Hammamatsu, Japan), in combination with either ET585/50 or ET700/75 (AHF Analysentechnik)). Excitation from a diode laser (Sapphire and Cube (Coherent, Germany), filtered either by ET535/70 or ZET640/10 (Chroma, USA)) was at 532nm and 640 nm with ≈50 W cm^−2^ at the sample location. To quantitatively characterize photostability, we imaged areas with the size of ≈25 × 35 μm containing >100 molecules. A movie was typically recorded for 300–600 s with an integration time of 100 ms. Fluorescent time traces were extracted by identifying pixels, which showed at least 2–3 s.d. above background noise (s.d. of all pixels over all frames of the movie) and summing the intensity in a 3 × 3 pixel area. Neighbouring peaks closer than 5 pixels were not taken into account (see typical examples of fluorophore density and fluorescent time traces in Supplementary Figs 3–11). The number of fluorescent spots in each frame image was determined using an absolute threshold criterion. The number of fluorescent emitters per image were than plotted over time and fitted to a mono-exponential decay *y*(*t*)=*C*+*A* × *e*^−*c* × *t*^ (with *c*=1/*τ*_bleach_ and *τ*_bleach_ being the characteristic bleaching time constant). For some samples with a more complicated behaviour, a double exponential decay of similar form was used and *τ*_bleach_ was calculated according to *τ*_bleach_=A_1_ × *τ*_1_+A_2_ × *τ*_2_ with amplitude normalization to 1. Bleaching times and associated s.d. were derived from multiple repeats of the same experiment on different days, where each compound was tested in ≥5 movies. The SNR ratio was determined using fluorescent time traces, by dividing the s.d. of the signal before photobleaching with the average fluorescence intensity during that period. The total number of detected photons before photobleaching was calculated by multiplying the obtained count rate and photobleaching lifetime.

*Single-molecule FRET and ALEX spectroscopy*. For data acquisition we used the same home-built confocal microscope as described above. Fluorescence photons arriving at the two detection channels (donor detection channel D or acceptor detection channel A) were assigned to either donor- or acceptor-based excitation, based on their photon arrival time as described previously^33^,^35^,^36^. Three relevant photon streams were extracted from the data corresponding to donor-based donor emission *F*(DD), donor-based acceptor emission *F*(DA) and acceptor-based acceptor emission *F*(AA). During diffusion, stoichiometry *S* and apparent FRET efficiencies E* were calculated for each fluorescent burst above a certain threshold yielding a two-dimensional (2D) histogram. Uncorrected FRET efficiency E* monitors the proximity between the two fluorophores and is calculated according to E*=*F*(DA)/(*F*(DD)+*F*(DA)). Stoichiometry *S* is defined as the ratio between the overall fluorescence intensity during the green excitation period over the total fluorescence intensity during both green and red periods, and describes the ratio of donor-to-acceptor fluorophores in the sample: *S*=(*F*(DA)+*F*(DD)/(*F*(DD)+*F*(DA)+*F*(AA)). Using published procedures to identify bursts corresponding to single molecules, we obtained bursts characterized by three parameters (*M*, *T* and *L*). A fluorescent signal is considered a burst provided it meets the following criteria: a total of *L* photons are collected, having *M* neighbouring photons within a time interval of *T* microseconds. We applied different burst search algorithms on the data sets: the sum of all three detection channels (all photons) using parameters *M*=15, *T*=500 μs and *L*=50 or a dual-colour burst search with *M*=12, *T*=500 μs and *L*=30; additional per-bin thresholding removed spurious changes in fluorescence intensity and selected for intense single-molecule bursts; precise treatment of each data set is given in each figure caption. Binning of the detected bursts into a 2D E*/*S* histogram yielded subpopulations that can be separated according to their *S*-values. Photon-counting histograms were obtained using similar thresholds as for the 2D E*/*S* histograms.

ALEX experiments were carried out at room temperature using 25–50 pM of double-labelled protein in imaging buffer: 50 mM KPi, pH 7.4, 150 mM KCl, pH 7.4, containing 10% (wt/vol) glucose and 10% (vol/vol) glycerine, 50 μg ml^−1^ glucose oxidase, 100–200 μg ml^−1^ catalase and 0.1 mM TCEP. For experiments with TX added to the imaging buffer, 2 mM TX, 50 mM KPi, pH 7.4, and 150 mM KCl were irradiated with ultraviolet light for 30 min before adding 10% (wt/vol) glucose and 10% (vol/vol) glycerine, 50 μg ml^−1^ glucose oxidase, 100–200 μg ml^−1^ catalase and 0.1 mM TCEP. Titration experiments were done via adding increasing concentrations of ligand to the imaging buffer (0, 1.0 and 200 μM of glutamine).

*Count rate versus excitation intensity*. The count rate per molecule dependence on the excitation intensity was recorded using 20–100 nM of labelled SBD2 protein in imaging buffer (10 mM KPi, 2.7 mM KCl, 137 mM NaCl, pH 7.4, 10% w/v glucose, 50 μg ml^−1^ glucose oxidase, 100–200 μg ml^−1^ catalase, and 0.1 mM TCEP, both in the presence and the absence of 2 mM aged TX. The count rate per molecule was acquired via excitation from the 543 and 633 nm laser on a LSM 710 confocal laser scanning microscope (Carl Zeiss, Jena, Germany) through a C-apochromat × 401.20 w Korr M27 water-immersion objective (NA=1.2). Excitation light from both lasers (at different intensities) was coupled into the objective by a dichroic beam splitter (MBS 458/543/633). Fluorescence was collected through the same objective with appropriate spectral filtering (NFT 635 VIS and LP 580).

*STED and confocal microscopy*. STED microscopy and the corresponding confocal microscopy was performed on an Abberior Instruments Resolft microscope (Abberior Instruments, Goettingen, Germany) to which a Ti:Sa STED laser was added (MaiTai, Newport-Spectra Physics; as outlined in detail in ref. 52). Excitation was performed at 640 nm (LDH-D-C-640P laser diode, Picoquant) and STED at 780 nm, at 80 MHz. Pulsing of the excitation laser was triggered by the Ti:Sa laser using a photodiode (APS-100-01), an amplifier (CON-TTL, both from Becker & Hickl, Berlin, Germany) along with a ps-delay unit (either Abberior Instruments or LAS-015617, MPD/Laser2000 UK). A donut-shaped intensity distribution of the STED laser focus was realized by the incorporation of a vortex phase plate (VPP-1a, RPC Photonics, Rochester, NY) into the collimated STED beam. Laser focusing and fluorescence collection was done by an oil-immersion microscope objective (UPlanSapo × 100/1.4 oil, Olympus, Japan). The collected fluorescence was detected by an avalanche photodiode (SPCM-AQRH-13, Excelitas Technologies) with a 650–690nm bandpass filter (670/40, AHF Analysentechnik).

*Immunolabelling*. Gm5756T human fibroblast were fixed with 3% paraformaldehyde for 10 min, rinsed several times in PBS, permeabilized in 0.1% Triton X-100 for 10 min, rinsed again several times in PBS and blocked in 2% BSA/5% FCS in PBS for 1 h. Samples were incubated with primary mouse antibodies, to stain nuclear pore diluted 1:500 in blocking buffer for 1 h at room temperature. Coverslips were washed five times in 1% BSA in PBS and then incubated for 30 min with goat anti-mouse antibodies (Abberior GmbH, Gottingen, Germany) diluted 1:250 in blocking buffer. Samples were washed five times in 1% BSA in PBS and mounted in the buffer system using an oxygen-scavenging system as used before in the single-molecule experiments.

## Additional information

**How to cite this article**: van der Velde, J. H. M. *et al*. A simple and versatile design concept for fluorophore derivatives with intramolecular photostabilization. *Nat. Commun*. 7:10144 doi: 10.1038/ncomms10144 (2016).

## Supplementary Material

Supplementary Information Supplementary Figures 1-36, Supplementary Methods and Supplementary References

Supplementary Movie 1 Fluorescence microscopy of single ATTO647N and NPA-ATTO647N show increased photostability

Supplementary Movie 2 Repeated STED imaging of nuclear pore complexes labelled with KK114 and NPA-KK114 show an increased number of obtainable images using intramolecular photostabilization
