# Corrigendum: A simple and versatile design concept for fluorophore derivatives with intramolecular photostabilization

**DOI:** 10.1038/ncomms16144

**Published:** 2017-08-04

**Authors:** Jasper H. M. van der Velde, Jens Oelerich, Jingyi Huang, Jochem H. Smit, Atieh Aminian Jazi, Silvia Galiani, Kirill Kolmakov, Giorgos Gouridis, Christian Eggeling, Andreas Herrmann, Gerard Roelfes, Thorben Cordes

Nature Communications 7 Article number: 10144 ; DOI: 10.1038/ncomms10144 (2016) Published: 01 11 2016; Updated: 08 04 2017

The original version of this Article contained an error in the spelling of the author Giorgos Gouridis which was incorrectly given as Giorgos Guoridis. This has now been corrected in both the PDF and HTML versions of the Article.

