# Corrigendum: A simple and versatile design concept for fluorophore derivatives with intramolecular photostabilization

**DOI:** 10.1038/ncomms16173

**Published:** 2017-11-16

**Authors:** Jasper H. M. van der Velde, Jens Oelerich, Jingyi Huang, Jochem H. Smit, Atieh Aminian Jazi, Silvia Galiani, Kirill Kolmakov, Giorgos Gouridis, Christian Eggeling, Andreas Herrmann, Gerard Roelfes, Thorben Cordes

Nature Communications 7: Article number: 10144; DOI: 10.1038/ncomms10144 (2016); Published: 01 11 2016; Updated: 11 16 2017

This Article contains an error in Fig. 4. Figure 4b shows the structure of the rhodamine dye Alexa488, not the Alexa555 used in this work. The structure of Alexa555 is not known.

