# Author Correction: A simple and versatile design concept for fluorophore derivatives with intramolecular photostabilization

**DOI:** 10.1038/ncomms16232

**Published:** 2018-07-24

**Authors:** Jasper H. M. van der Velde, Jens Oelerich, Jingyi Huang, Jochem H. Smit, Atieh Aminian Jazi, Silvia Galiani, Kirill Kolmakov, Giorgos Gouridis, Christian Eggeling, Andreas Herrmann, Gerard Roelfes, Thorben Cordes

Nature Communications 7: Article number: 10144; DOI: 10.1038/ncomms10144 (2016); Published 01 11 2016, Updated 07 24 2018

The original version of this Article omitted the following from the Acknowledgements: ‘This work was also financed by an ERC starting grant ‘SM-IMPORT’ (No. 638536 to T.C.)’. This has been corrected in both the PDF and HTML versions of the Article.

