# Supplementary material for: A simple and versatile design concept for fluorophore derivatives with intramolecular photostabilization
Source: Nat Commun. 2016 Jan 11;7:10144. doi: 10.1038/ncomms10144 (PMC4729898; doi:10.1038/ncomms10144)
Supplement: Supplementary Information — Supplementary Figures 1-36, Supplementary Methods and Supplementary References [file ncomms10144-s1.pdf]

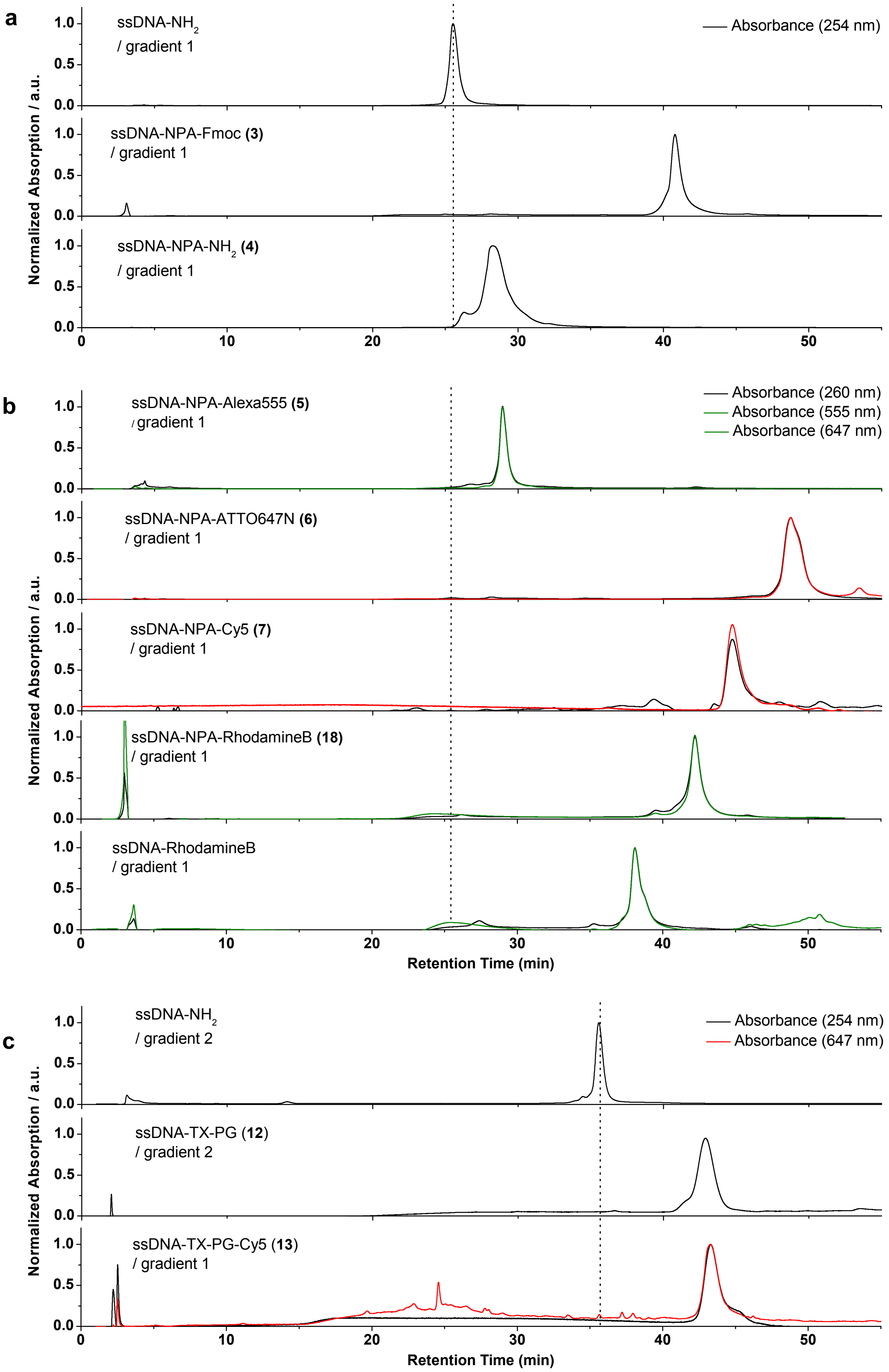

Supplementary Figure 1. HPLC-characterization and purification of functionalized oligonucleotides: ssDNA-fluorophore and ssDNA-NPA-fluorophore (**5, 6, 7, 13** and**18**).

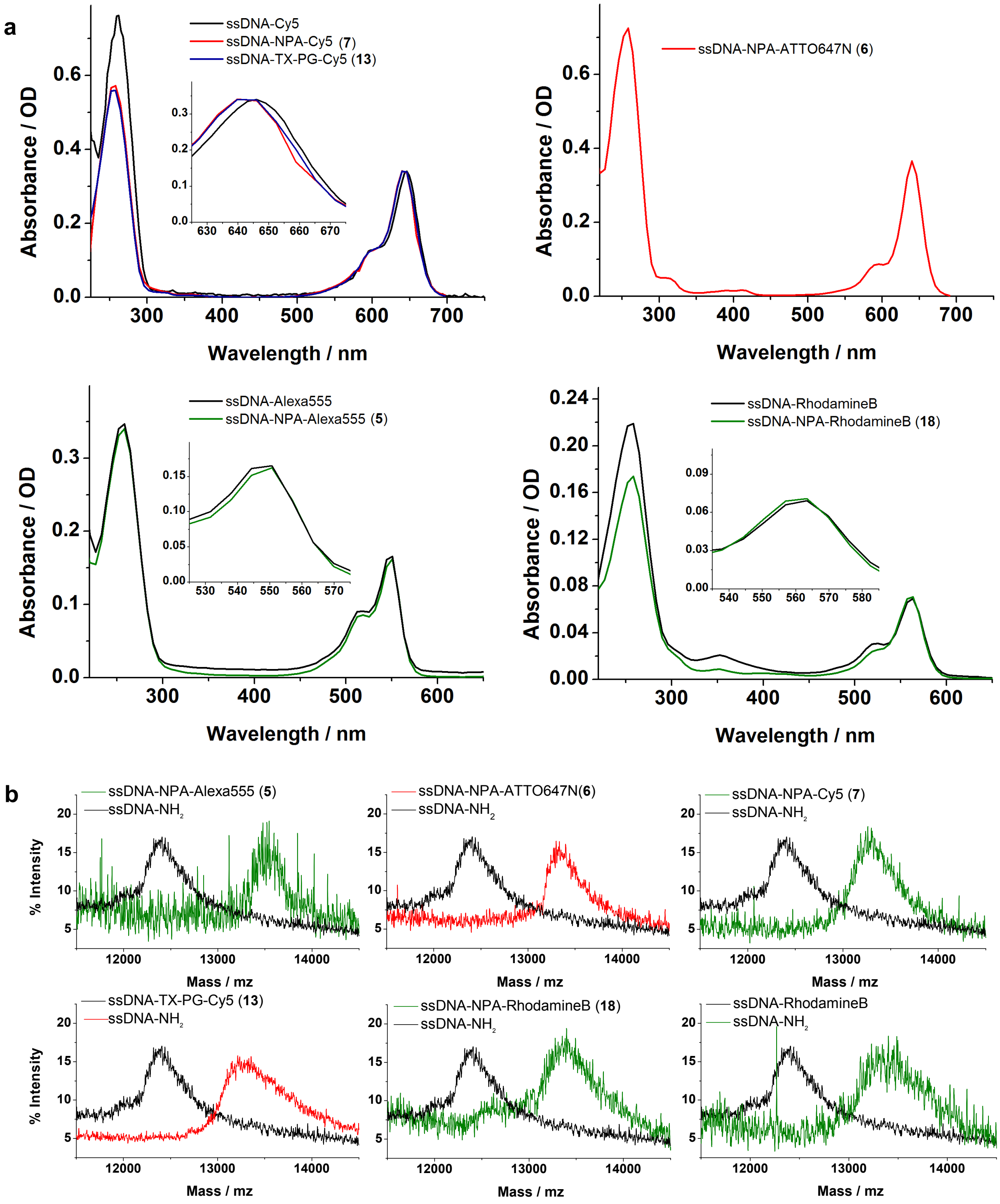

Supplementary Figure 2. a) UV-VIS absorption spectra of functionalized oligonucleotides: ssDNA-fluorophore and ssDNA-NPA-fluorophore (**5, 6, 7, 13** and **18**) showing the characteristic peak of DNA in the UV-range and the strong visible absorption of the dye. **b**) Maldi-TOF mass spectra DNA-photostabilizer-dye conjugates. The comparison of the spectra of the non-modified DNA with that of the conjugates clearly indicates an increase of the molecular mass originating from coupling of dye (and stabilizer).

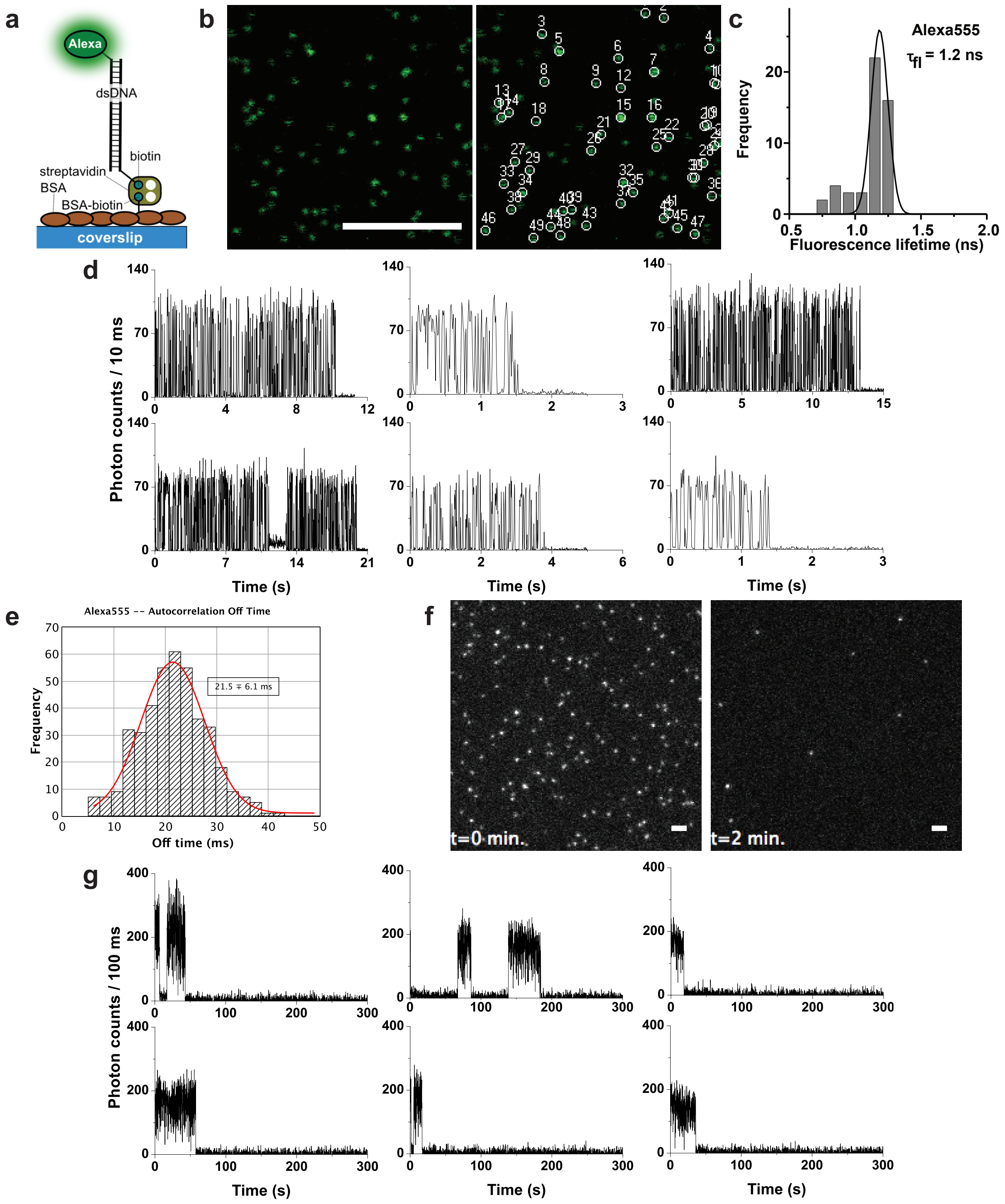

Supplementary Figure 3. Further details of photophysical characterization of Alexa555. (**a**) Schematic view of the photostabilizer-dye conjugate on DNA and the experimental strategy for surface immobilization. (**b**) Confocal scanning image of immobilized Alexa555 molecules, including the identification of positions of single emitters for further analysis (right panel) (scale bar 5 µm). (**c**) Histogram of fluorescence lifetimes determined for each of these single emitters, including Gaussian fit (black line) and average lifetime value τ_{fl}. (**d**) Representative fluorescence time traces of selected single emitters. (**e**) Histogram of off-state lifetimes, with Gaussian fit (red line) and mean plus standard deviation value derived from autocorrelation analysis of multiple molecules. (**f**) Representative TIRF images of immobilized Alexa555 molecules at two different time points (0 and 2 min, as given) after starting illumination and recording (scale bar 2 µm). (**g**) Representative fluorescence time traces of single emitters from TIRF recordings, indicating long-term blinking and photobleaching characteristics. Excitation intensities are 0.3 kW cm^{-2} for confocal and 50 W cm^{-2} for TIRF-experiments (excitation at 532 nm, detection with HC582/75). Brightness and contrast settings 1950 (low) to 10000 (high) and 3 (low) to 60 (high) for TIRF and confocal images, respectively.

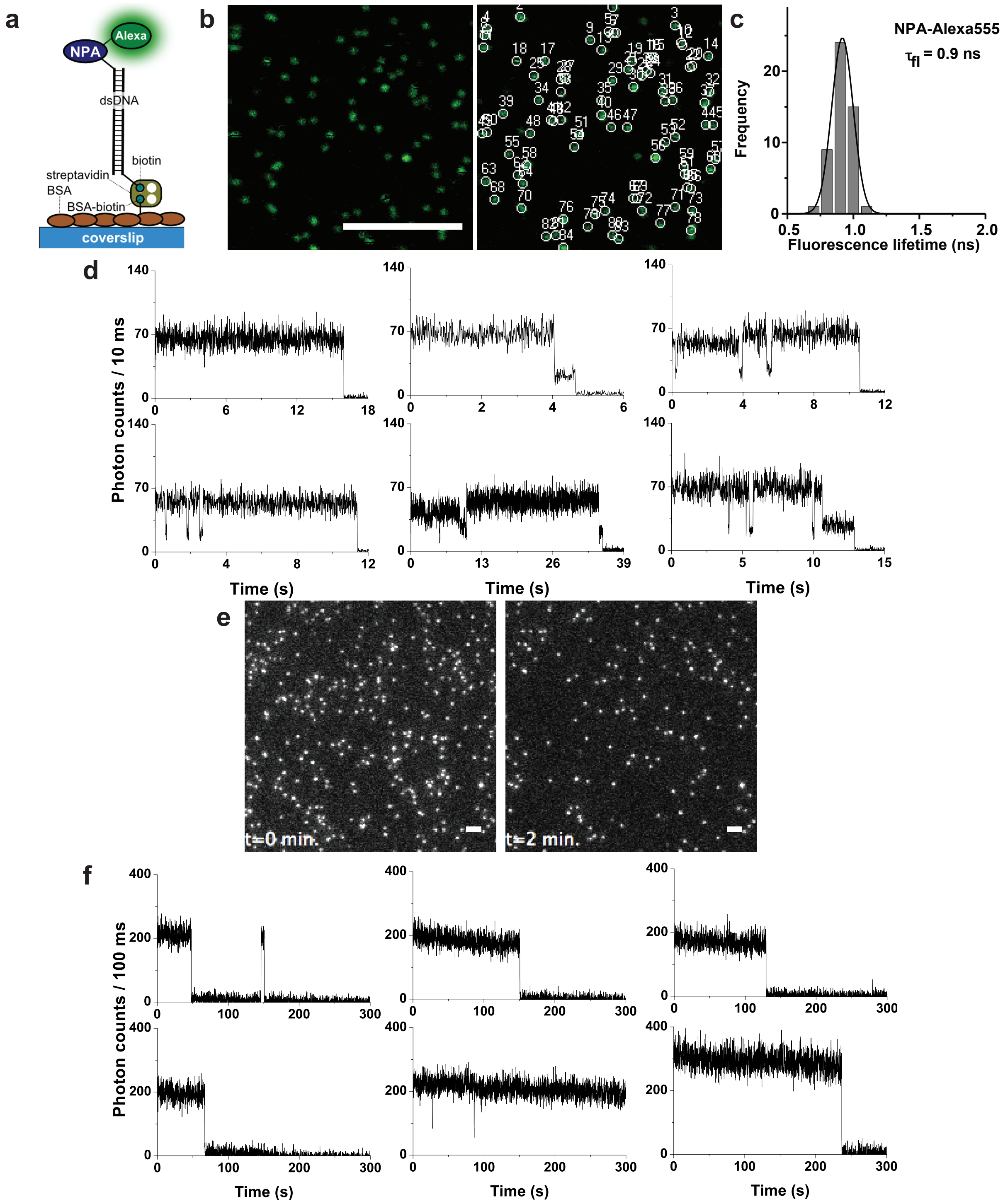

Supplementary Figure 4. Further details of photophysical characterization of NPA-Alexa555. (**a**) Schematic view of the photostabilizer-dye conjugate on DNA and the experimental strategy for surface immobilization. (**b**) Confocal scanning image of immobilized NPA-Alexa555 molecules, including the identification of positions of single emitters for further analysis (right panel) (scale bar 5 µm). (**c**) Histogram of fluorescence lifetimes determined for each of these single emitters, including Gaussian fit (black line) and average lifetime value τ_{fl}. (**d**) Representative fluorescence time traces of the selected single emitters. (**e**) Representative TIRF images of immobilized NPA-Alexa555 molecules at two different time points (0 and 2 min, as given) after starting illumination and recording (scale bar 2 µm). (**f**) Representative fluorescence time traces of single emitters from TIRF recordings, indicating long-term blinking and photobleaching characteristics. Excitation intensities are 0.3 kW cm^{-2} for confocal and 50 W cm^{-2} for TIRF-experiments (excitation at 532 nm, detection with HC582/75). Brightness and contrast settings 1950 (low) to 10000 (high) and 3 (low) to 60 (high) for TIRF and confocal images, respectively.

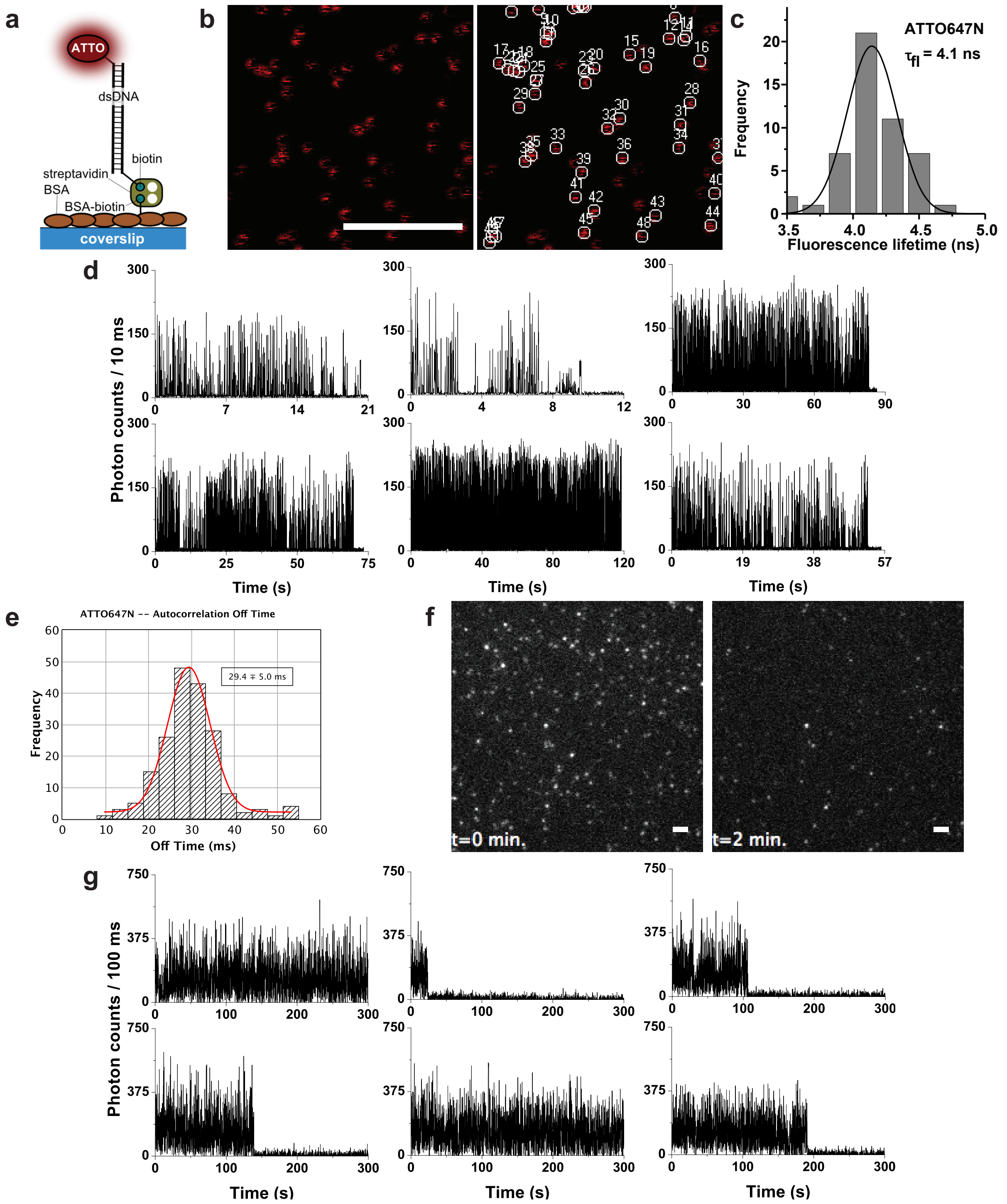

Supplementary Figure 5. Further details of photophysical characterization of ATTO647N. (**a**) Schematic view of the photostabilizer-dye conjugate on DNA and the experimental strategy for surface immobilization. (**b**) Confocal scanning image of immobilized ATTO647N molecules, including the identification of positions of single emitters for further analysis (right panel) (scale bar 5 µm). (**c**) Histogram of fluorescence lifetimes determined for each of these single emitters, including Gaussian fit (black line) and average lifetime value τ_{fl}. (**d**) Representative fluorescence time traces of the selected single emitters. (**e**) Histogram of off-state lifetimes, with Gaussian fit (red line) and mean plus standard deviation value derived from autocorrelation analysis of multiple molecules. (**f**) Representative TIRF images of immobilized ATTO647N molecules at two different time points (0 and 2 min, as given) after starting illumination and recording (scale bar 2 µm). (**g**) Representative fluorescence time traces of single emitters from TIRF recordings, indicating long-term blinking and photobleaching characteristics. Excitation intensities are 0.66 kW cm^{-2} for confocal measurement and 50 W cm^{-2} for TIRF-experiments (excitation at 640 nm, detection with ET700/75). Brightness and contrast settings 3100 (low) to 16000 (high) and 5 (low) to 100 (high) for TIRF and confocal images, respectively.

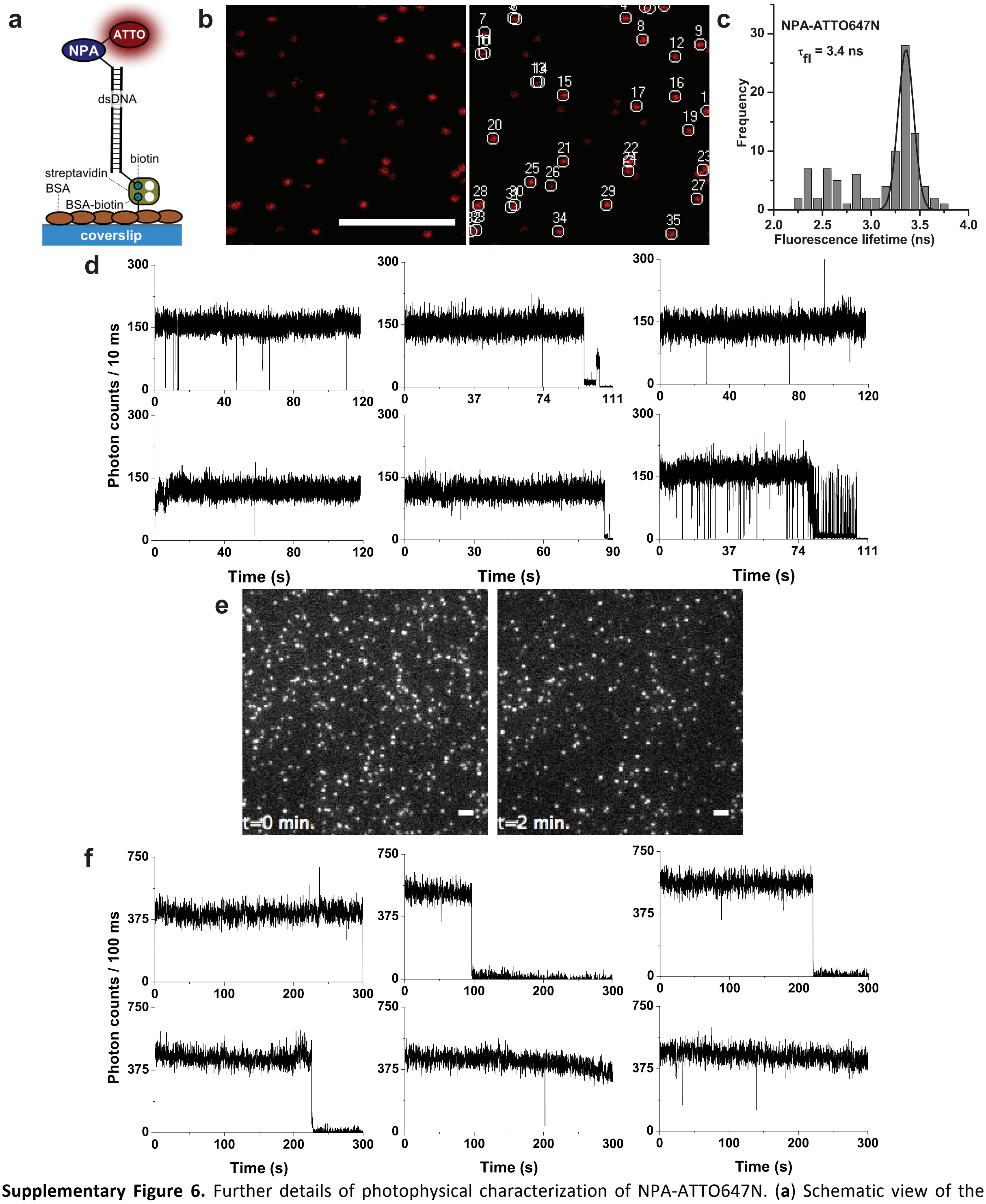

Supplementary Figure 6. Further details of photophysical characterization of NPA-ATTO647N. (**a**) Schematic view of the photostabilizer-dye conjugate on DNA and the experimental strategy for surface immobilization. (**b**) Confocal scanning image of immobilized NPA-ATTO647N molecules, including the identification of positions of single emitters for further analysis (right panel) (scale bar 5 µm). (**c**) Histogram of fluorescence lifetimes determined for each of these single emitters, including Gaussian fit (black line) and average lifetime value τ_{fl}. (**d**) Representative fluorescence time traces of the selected single emitters. (**e**) Representative TIRF images of immobilized NPA-ATTO647N molecules at two different time points (0 and 2 min, as given) after starting illumination and recording (scale bar 2 µm). (**f**) Representative fluorescence time traces of single emitters from TIRF recordings, indicating long-term blinking and photobleaching characteristics. Excitation intensities are 0.66 kW cm^{-2} for confocal measurement and 50 W cm^{-2} for TIRF-experiments (excitation at 640 nm, detection with ET700/75). Brightness and contrast settings 3100 (low) to 16000 (high) and 5 (low) to 100 (high) for TIRF and confocal images, respectively.

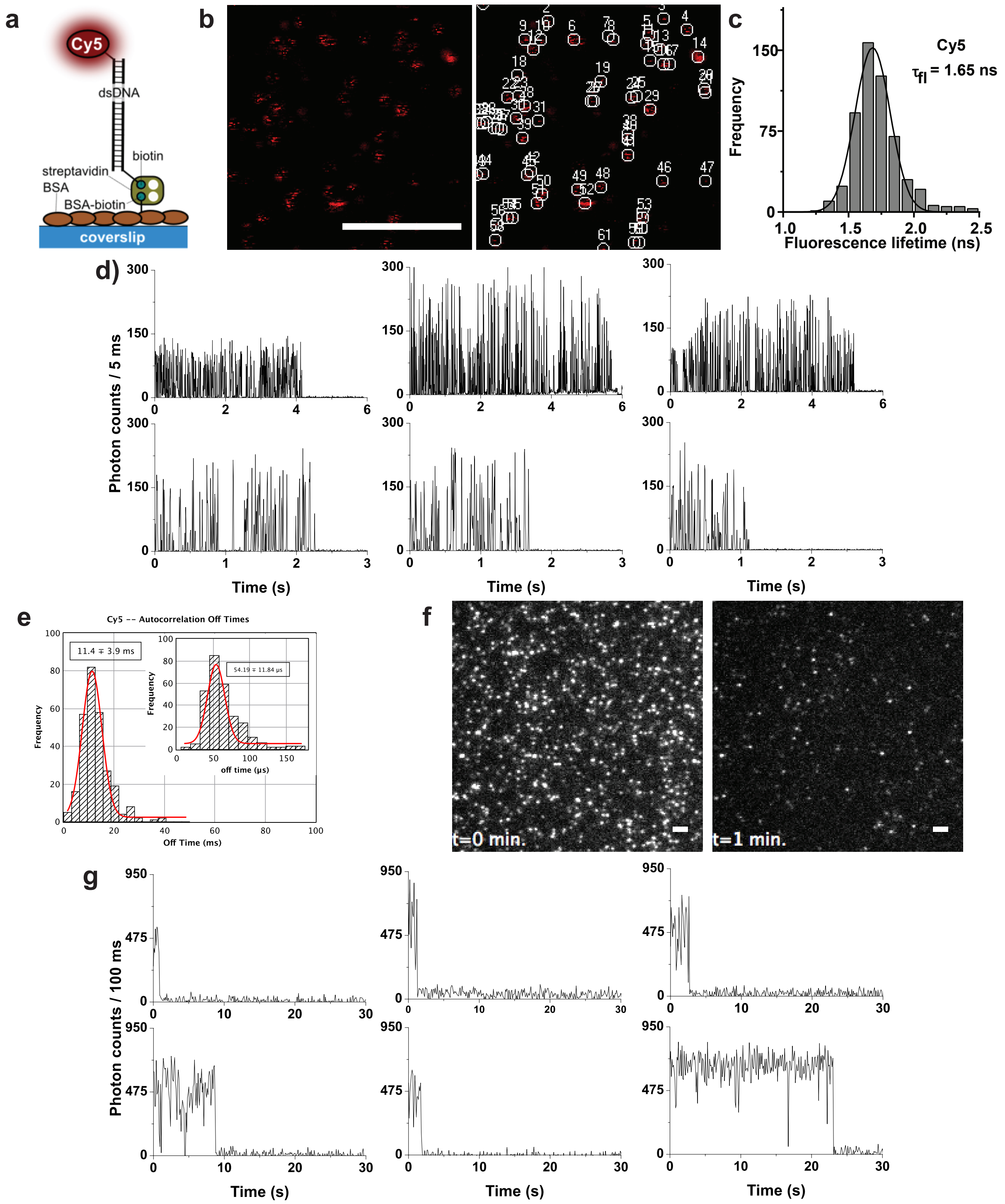

Supplementary Figure 7. Details of photophysical characterization of Cy5. (**a**) Schematic view of the photostabilizer-dye conjugate on DNA and the experimental strategy for surface immobilization. (**b**) Confocal scanning image of immobilized Cy5 molecules, including the identification of positions of single emitters for further analysis (right panel) (scale bar 5 µm). (**c**) Histogram of fluorescence lifetimes determined for each of these single emitters, including Gaussian fit (black line) and average lifetime value τ_{fl}. (**d**) Representative fluorescence time traces of the selected single emitters. (**e**) Histogram of off-state lifetimes (longer (main panel: triplet) and shorter (inset: cis-trans isomerization) lifetimes), with Gaussian fit (red line) and mean plus standard deviation value derived from autocorrelation analysis of multiple molecules. (**f**) Representative TIRF images of immobilized Cy5 molecules at two different time points (0 and 2 min, as given) after starting illumination and recording (scale bar 2 µm). (**g**) Representative fluorescence time traces of single emitters from TIRF recordings, indicating long-term blinking and photobleaching characteristics. Excitation intensities are 0.66 kW cm^{-2} for confocal measurement and 50 W cm^{-2} for TIRF-experiments (excitation at 640 nm, detection with ET700/75). Brightness and contrast settings 4500 (low) to 22000 (high) and 5 (low) to 100 (high) for TIRF and confocal images, respectively.

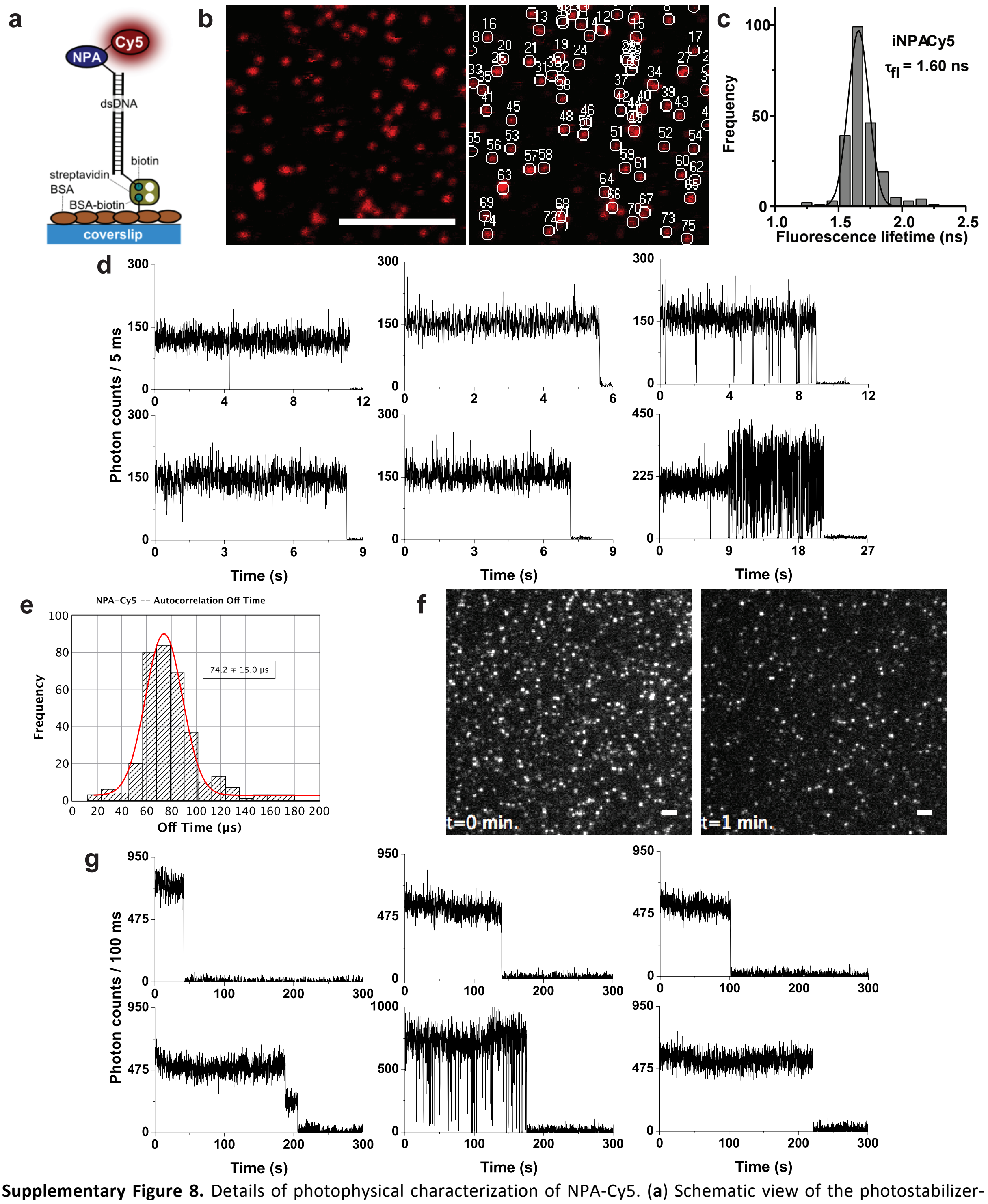

Supplementary Figure 8. Details of photophysical characterization of NPA-Cy5. (**a**) Schematic view of the photostabilizer-dye conjugate on DNA and the experimental strategy for surface immobilization. (**b**) Confocal scanning image of immobilized NPA-Cy5 molecules, including the identification of positions of single emitters for further analysis (right panel) (scale bar 5 µm). (**c**) Histogram of fluorescence lifetimes determined for each of these single emitters, including Gaussian fit (black line) and average lifetime value τ_{fl}. (**d**) Representative fluorescence time traces of the selected single emitters. (**e**) Histogram of off-state (cis-trans isomerization) lifetimes, with Gaussian fit (red line) and mean plus standard deviation value derived from autocorrelation analysis of multiple molecules. (**f**) Representative TIRF images of immobilized NPA-Cy5 molecules at two different time points (0 and 2 min, as given) after starting illumination and recording (scale bar 2 µm). (**g**) Representative fluorescence time traces of single emitters from TIRF recordings, indicating long-term blinking and photobleaching characteristics. Excitation intensities are 0.66 kW cm^{-2} for confocal measurement and 50 W cm^{-2} for TIRF-experiments (excitation at 640 nm, detection with ET700/75). Brightness and contrast settings 4500 (low) to 22000 (high) and 5 (low) to 100 (high) for TIRF and confocal images, respectively.

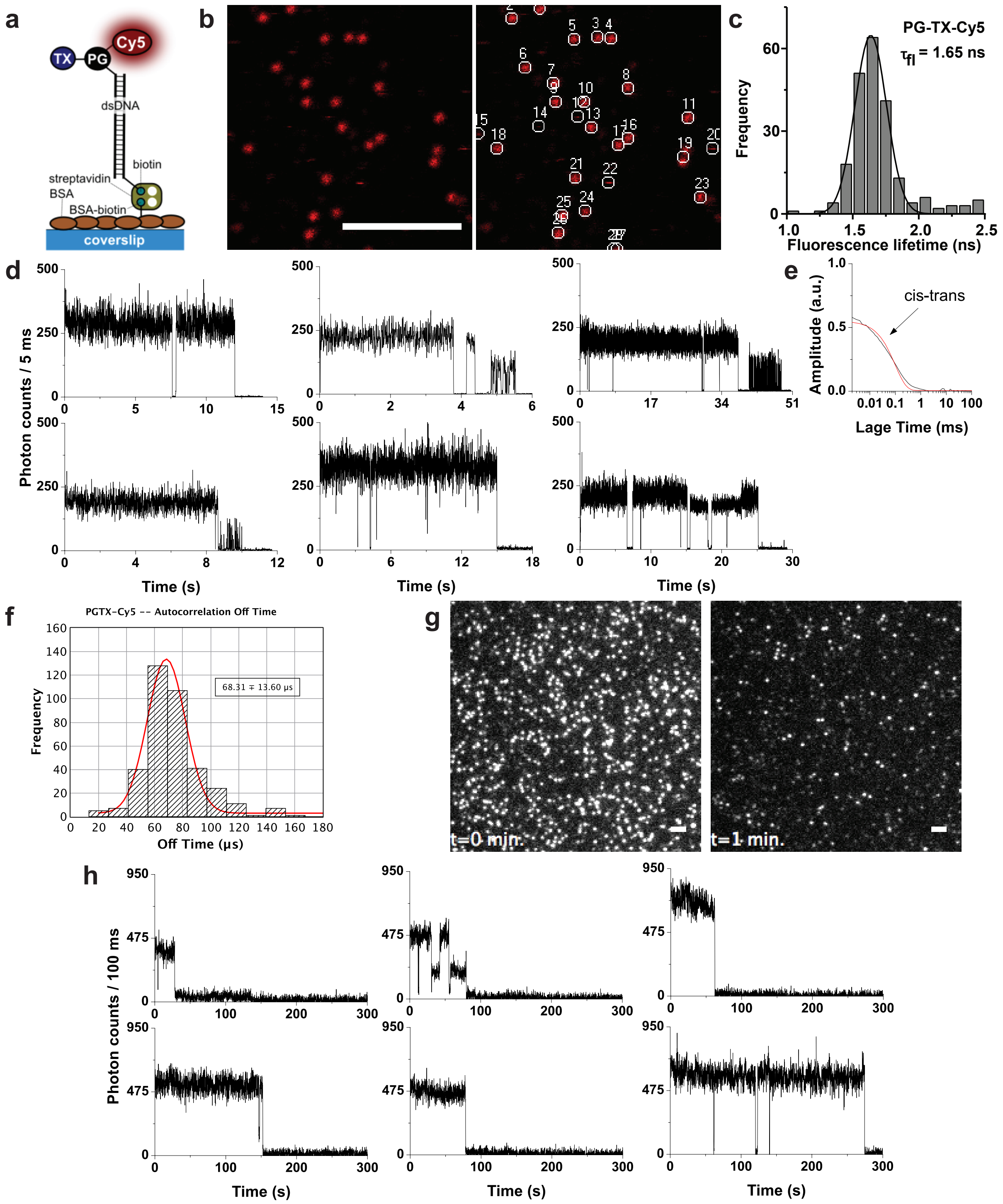

Supplementary Figure 9. Details of photophysical characterization of TX-PG-Cy5. (**a**) Schematic view of the photostabilizer-dye conjugate on DNA and the experimental strategy for surface immobilization. (**b**) Confocal scanning image of immobilized TX-PG-Cy5 molecules, including the identification of positions of single emitters for further analysis (right panel) (scale bar 5 µm). (**c**) Histogram of fluorescence lifetimes determined for each of these single emitters, including Gaussian fit (black line) and average lifetime value τ_{fl}. (**d**) Representative fluorescence time traces of the selected single emitters. (**e**) Autocorrelation decay of a single emitter (black, with the fit in red). (**f**) Histogram of off-state lifetimes, with Gaussian fit (red line) and mean plus standard deviation value derived from autocorrelation analysis of multiple molecules. (**g**) Representative TIRF images of immobilized TX-PG-Cy5 molecules at two different time points (0 and 2 min, as given) after starting illumination and recording (scale bar 2 µm). (**h**) Representative fluorescence time traces of single emitters from TIRF recordings, indicating long-term blinking and photobleaching characteristics. Excitation intensities are 0.66 kW cm^{-2} for confocal measurement and 50 W cm^{-2} for TIRF-experiments (excitation at 640 nm, detection with ET700/75). Brightness and contrast settings 4500(low) to 22000 (high) and 5 (low) to 100 (high) for TIRF and confocal images, respectively.

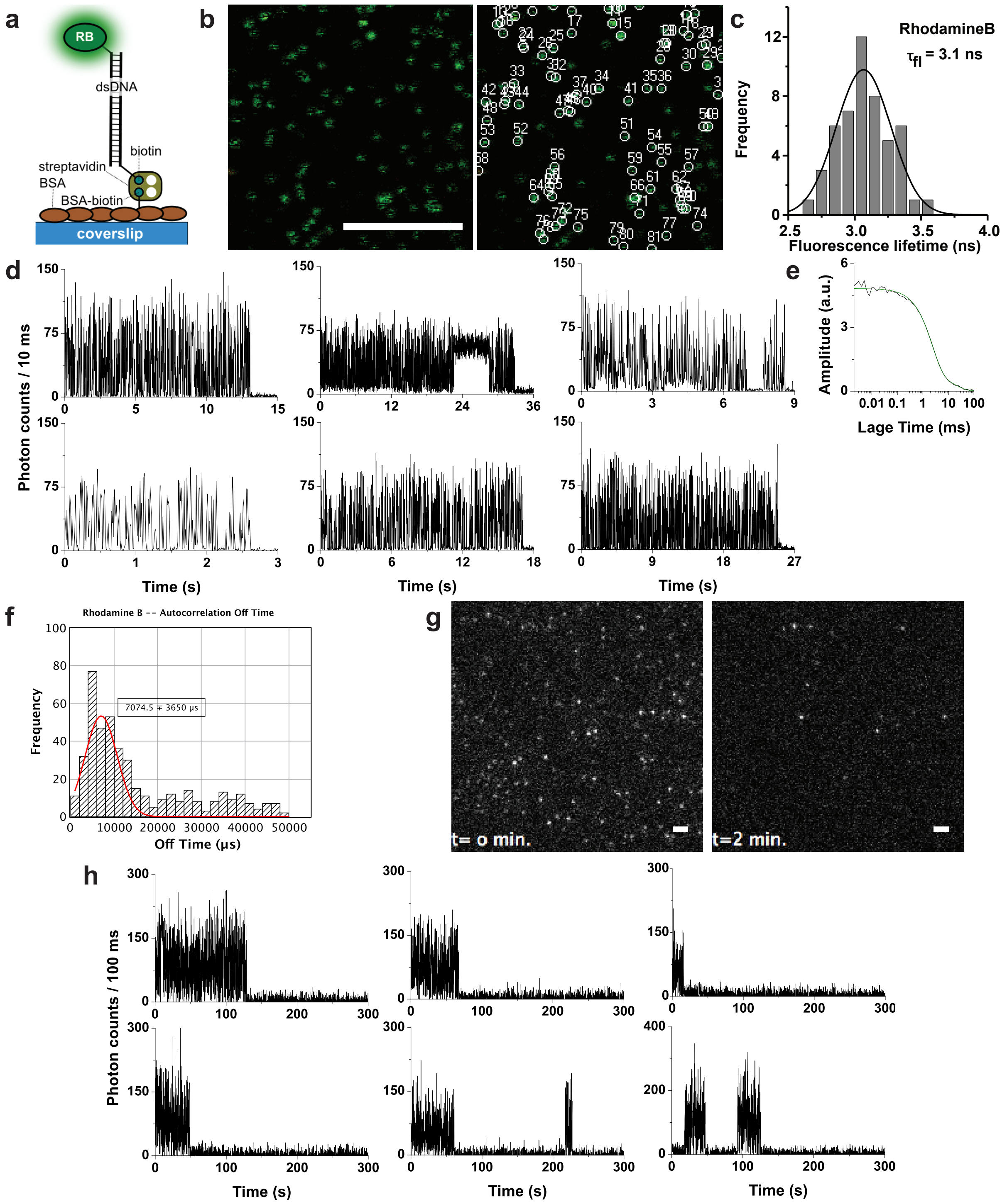

Supplementary Figure 10. Details of photophysical characterization of RhodamineB. (**a**) Schematic view of the photostabilizer-dye conjugate on DNA and the experimental strategy for surface immobilization. (**b**) Confocal scanning image of immobilized RhodamineB molecules, including the identification of positions of single emitters for further analysis (right panel) (scale bar 5 µm). (**c**) Histogram of fluorescence lifetimes determined for each of these single emitters, including Gaussian fit (black line) and average lifetime value τ_{fl}. (**d**) Representative fluorescence time traces of the selected single emitters. (**e**) Autocorrelation decay of a single emitter (black, with the fit in red) (**f**) Histogram of off-state lifetimes, with Gaussian fit (red line) and mean plus standard deviation value derived from autocorrelation analysis of multiple molecules. (**g**) Representative TIRF images of immobilized RhodamineB molecules at two different time points (0 and 2 min, as given) after starting illumination and recording (scale bar 2 µm). (**h**) Representative fluorescence time traces of single emitters from TIRF recordings, indicating long-term blinking and photobleaching characteristics. Excitation intensities are 4 kW cm^{-2} for confocal measurement and 50 W cm^{-2} for TIRF-experiments (excitation at 532 nm, detection with HC582/75). Brightness and contrast settings 2300 (low) to 6800 (high) and 3 (low) to 50 (high) for TIRF and confocal images, respectively.

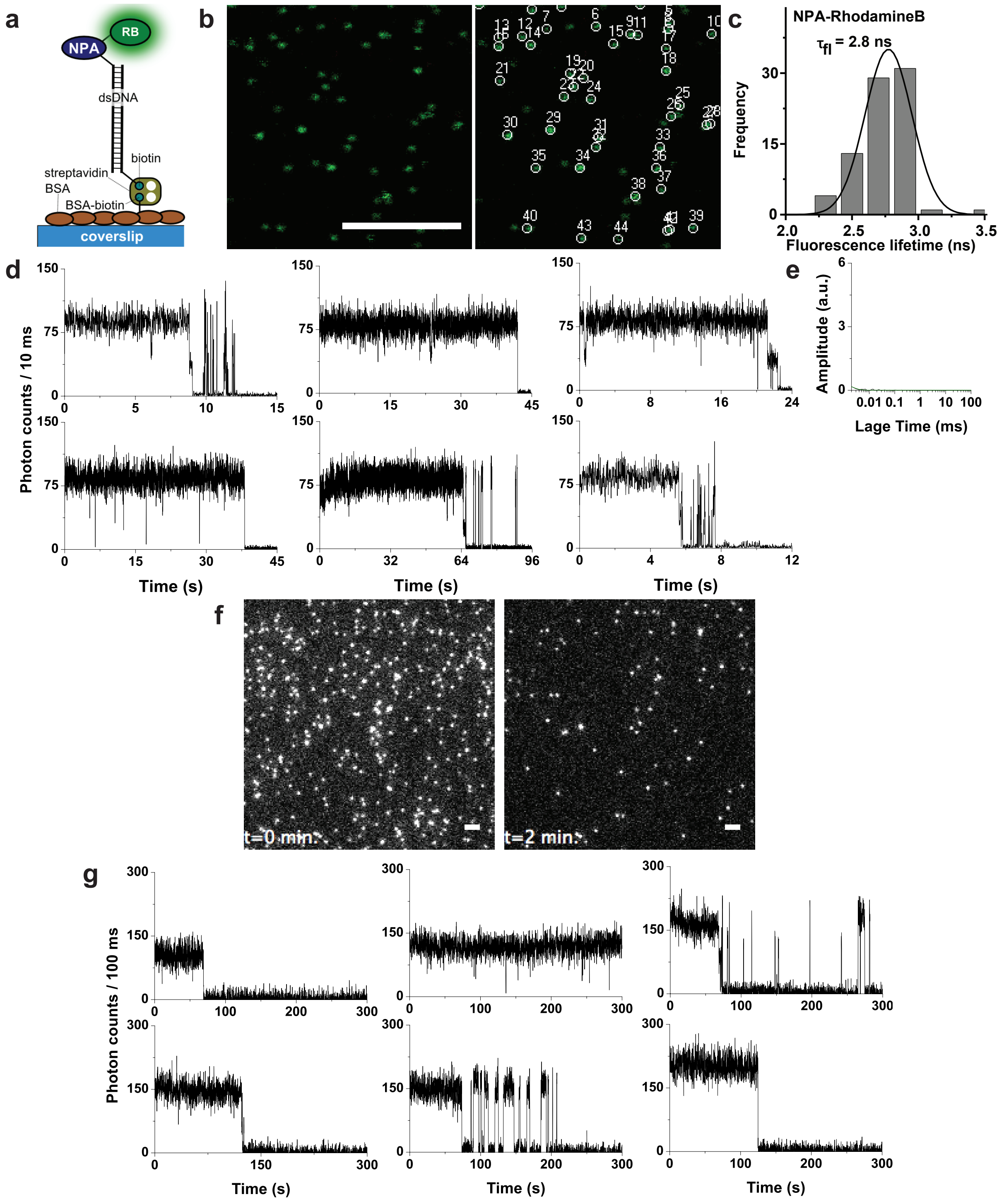

Supplementary Figure 11. Detailed photophysical characterization of NPA-RhodamineB. (**a**) Schematic view of the photostabilizer-dye conjugate on DNA and the experimental strategy for surface immobilization. (**b**) Confocal scanning image of immobilized NPA-RhodamineB molecules, including the identification of positions of single emitters for further analysis (right panel) (scale bar 5 µm). (**c**) Histogram of fluorescence lifetimes determined for each of these single emitters, including Gaussian fit (black line) and average lifetime value τ_{fl}. (**d**) Representative fluorescence time traces of the selected single emitters. (**e**) Autocorrelation decay of a single emitter (black, with the fit in red). (**f**) Representative TIRF images of immobilized NPA-RhodamineB molecules at two different time points (0 and 2 min, as given) after starting illumination and recording (scale bar 2 µm). (**g**) Representative fluorescence time traces of single emitters from TIRF recordings, indicating long-term blinking and photobleaching characteristics. Excitation intensities are 4 kW cm^{-2} for confocal measurement and 50 W cm^{-2} for TIRF-experiments (excitation at 532 nm, detection with HC582/75). Brightness and contrast settings 2300 (low) to 6800 (high) and 3 (low) to 50 (high) for TIRF and confocal images, respectively.

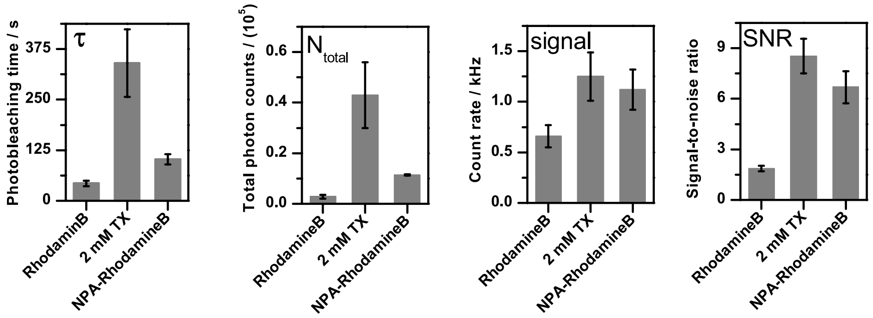

Supplementary Figure 12. Average and standard deviation of the mean (error bars) of photophysical parameters of RhodamineB without and with Trolox (2mM TX) and of NPA-RhodamineB, as determined from the TIRF data of Supplementary Figures 10 and 11: photobleaching time τ (left panel), total number of detected photons N_{total} (middle left), brightness (middle right), and signal-to-noise ratio (SNR, right panel). Recorded in aqueous PBS buffer at pH 7.4 in the absence of oxygen (continuous 532 nm excitation at ≈50 W cm^{-2}). Values in bar graphs were derived from N > 1000 molecules.

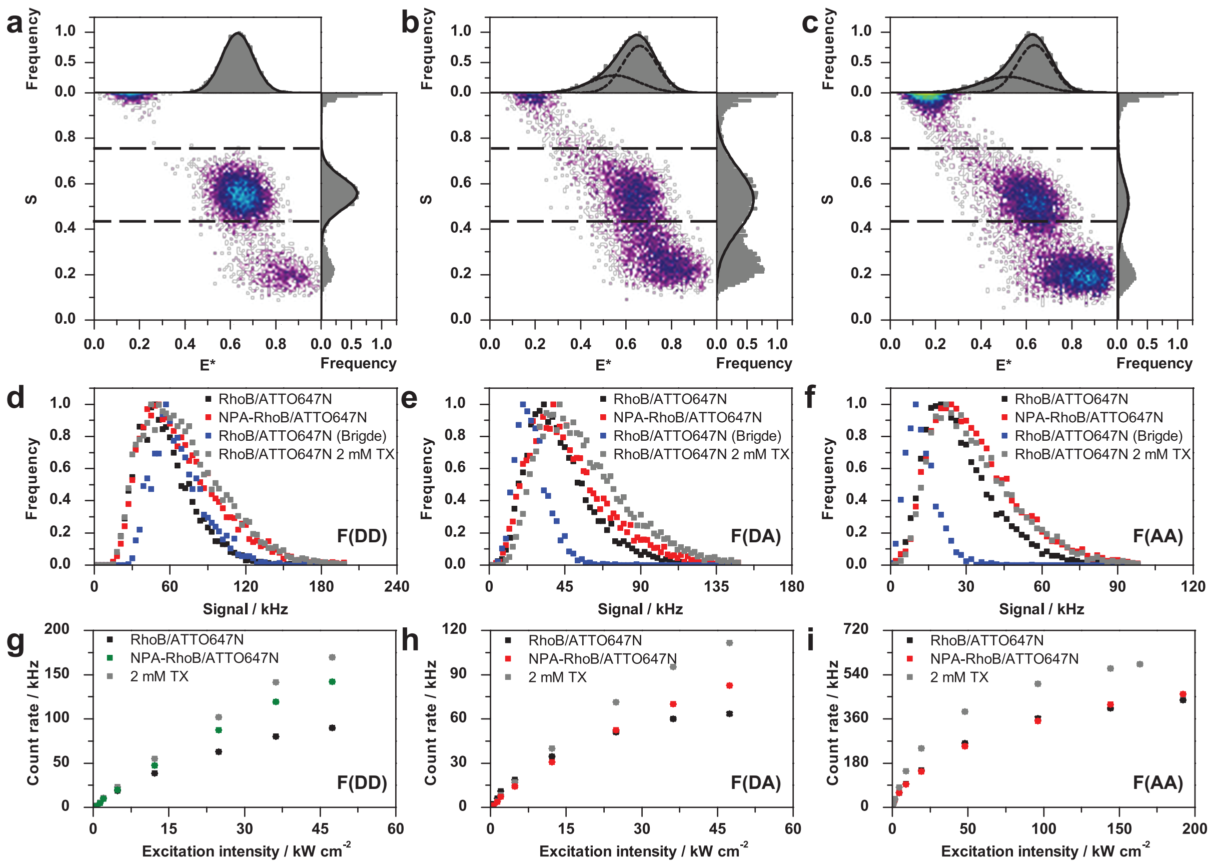

Supplementary Figure 13. a/b/c) Two-dimensional histograms (2D) of joint pair values of S (labeling stoichiometry, dashed lines 0.42 < S < 0.75 donor-acceptor population DA) and E* (FRET-efficiency, i.e., interprobe distance) of Cy3B/ATTO647N in the presence (**a**) and absence (**b**) of 2 mM TX, and Cy3B/NPA-ATTO647N (**c**) without ligand glutamine. Excitation intensities of 30 kW cm^{-2} at 532 nm and 10 kW cm^{-2} at 640 nm; data evaluated with all photon burst search and displayed with additional per-bin thresholds of F(DD)+F(DA)+F(AA)>150 and minimal number of counts per bin in ALEX histogram of 3. **d/e/f)** Histogram of fluorophore brightness values as determined from PCH on single-molecule transits of labeled SBD2 diffusing through the observation volume of our confocal microscope: comparison of donor-brightness F(DD) (**d**) acceptor-brightness when excited via FRET, F(DA) (**e**), and acceptor-brightness via direct red excitation, F(AA) (**e**). Excitation intensities of 30 kW/cm^2 at 532 nm and 20 kW/cm^2 at 640 nm. Note the varying S-ranges used for data evaluation using only bursts for the PCH corresponding to 0.75<S<0.42 for Cy3B/ATTO647N (+/- 2 mM TX) and Cy3B/NPA-ATTO647N, but 0.42<S<0.32 for the bridge in Cy3B/ATTO647N. **g/h/i)** Dependence of the mean count rate of F(DD), F(DA) and F(AA) of the different samples for increasing excitation intensity.

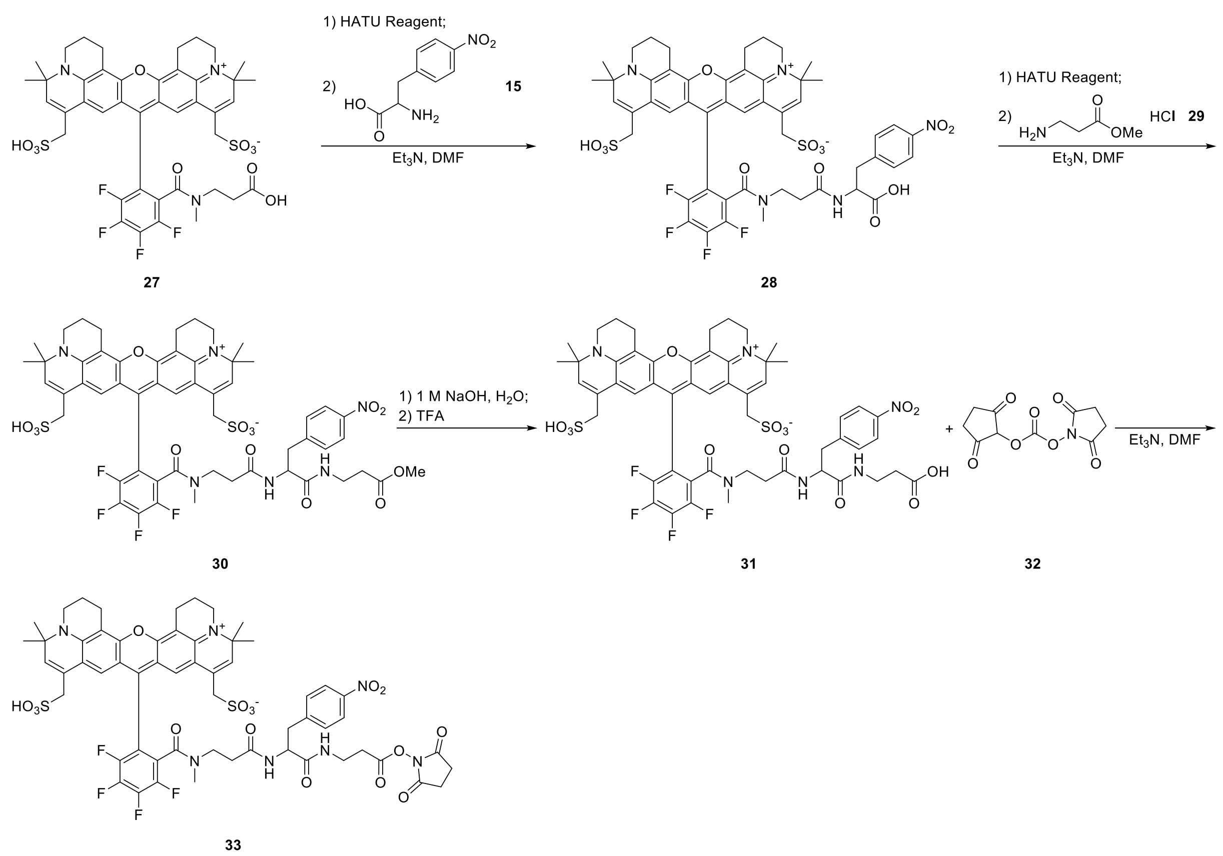

Supplementary Figure 14. Synthesis of reactive photostabilizer-dye conjugates of KK114 used for direct labeling of antibodies.

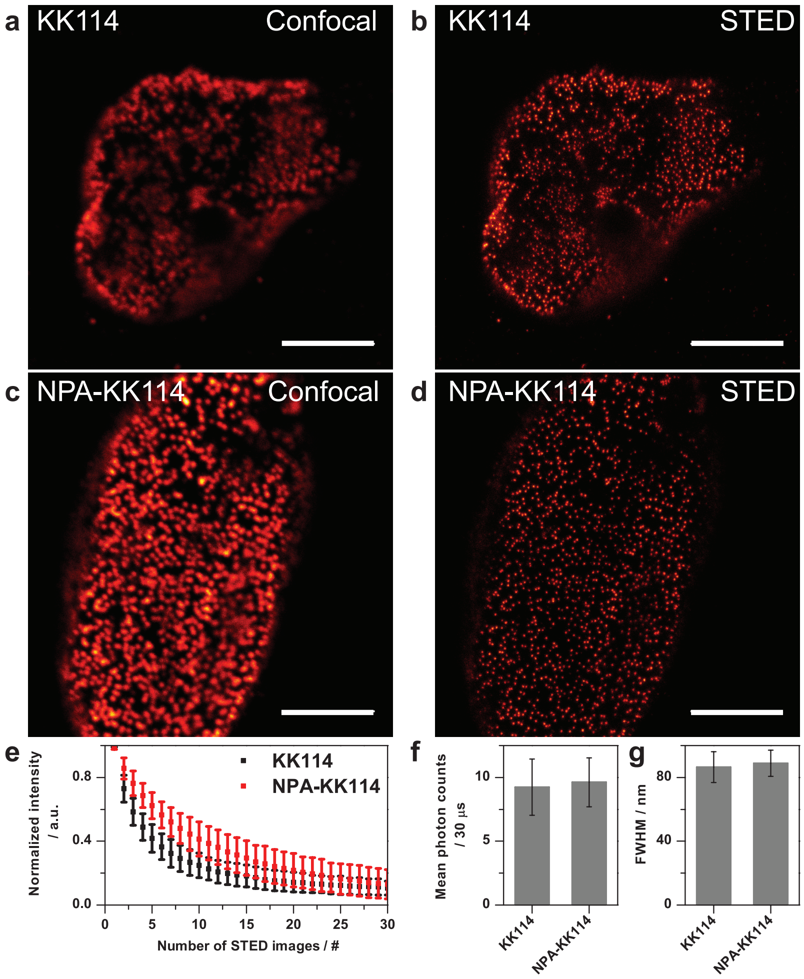

Supplementary Figure 15. Comparison of performance of KK114 and NPA-KK114 fluorophores for super-resolution STED microscopy of immunoloabeled nuclear pore complexes in fixed fibroblast cells. Confocal (**a**,**c**) and STED (**b**,**d**) images of a representative cell stained with KK114 (**a**,**b**) and NPA-KK114 (**c**,**d**). (scale bars 5 µm). (**e**) Total image intensity with number of repeated scan of the same area of the cells in the STED mode for KK114 (black) and NPA-KK114 (red), indicating reduced photobleaching in the case of NPA-KK114. Data from ≥ 3 measurement series, with error bars depicting the standard deviation. (**f**) Average brightness and standard deviation (error bars) for KK114 and NPA-KK114 labeling. (**g**) Size (full-width-at-half-maximum FWHM) of nuclear pore complexes in the initial STED images (n > 300 nuclear pore complexes) for KK114 and NPA-KK114 labeling.

Supplementary Methods

2 ("NPA-NHS") *(S)-2,5-dioxopyrrolidin-1-yl-2-((((9H-fluoren-9-yl)methoxy)carbonyl)amino)-3-(4-nitrophenyl)propanoate:* To **1** (173 mg, 0.4 mmol) and N-hydroxysuccinimide (NHS) (46 mg, 0.4 mmol), in 1.5 mL anhydrous 1,4-dioxane, N,N'-dicyclohexyl carbodiimide (DCC) (83 mg, 0.4 mmol) was added at 0°C. After stirring at ambient temperature overnight, the mixture was cooled to 10°C and the precipitate was filtered. The filtrate was evaporated in vacuo. Residual 1,4-dioxane was removed by subsequent addition and evaporation of anhydrous ethanol. NPA-NHS was used for coupling without further purification. For analysis NPA-NHS (**2**) was purified by column chromatography (SiO_2, chloroform) and isolated as colorless oil. Yield: 85 mg (0.160 mmol, 40%). ^{1}H NMR (400 MHz, $CDCl_3$) δ 8.14 (d, *J*=8.2, 2H), 7.78 (d, *J*=7.4, 2H), 7.55 (d, *J*=7.3, 2H), 7.37 (m, 6H), 5.19 – 5.03 (m, 2H), 4.58 (dd, *J*=10.6, 6.5, 1H), 4.45 (dd, *J*=11.0, 6.2, 1H), 4.20 (t, *J*=6.4, 1H), 3.40 (dd, *J*=14.3, 5.4, 1H), 3.31 (dd, *J*=14.1, 5.1, 1H), 2.87 (s, 4H). ^{13}C NMR (101 MHz, $CDCl_3$) δ = 168.4, 166.8, 155.1, 147.4, 143.6, 143.3, 141.9, 141.4, 141.3, 130.7, 127.9, 127.1, 124.9, 124.8, 123.8, 120.1, 120.0, 67.0, 52.7, 47.1, 38.0, 25.6. HRMS (positive): ($C_{28}H_{23}N_3NaO_8^+$) 552.1355 (found M+Na), 552.1377 (calc.).

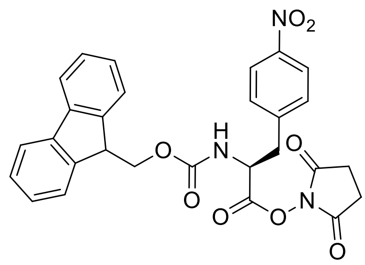

10 ("TX-PG") *2-(6-hydroxy-2,5,7,8-tetramethylchroman-2-carboxamido)pent-4-ynoic acid:* A solution of DL-propangylglycine **8** (136 mg, 1.20 mmol) and K_2CO_3 (498 mg, 3.60 mmol) in 4 ml of H_2O was added to a solution of Trolox-NHS **9** (417 mg, 1.20 mmol) in 2 ml of 1,4-dioxane and stirred at room temperature until full conversion of the activated ester (~3 hours). 5 ml of H_2O was added and the mixture was acidified to pH 1. The aqueous solution was extracted by EtOAc (3 x 10 ml). The combined organic phases were dried over Na_2SO_4 and evaporated under reduced pressure to give the crude product. After flash column chromatography (SiO_2, DCM/EtOH: 100/0 to 80/20) the product was isolated as yellow sticky oil. Yield: 360 mg (1.043 mmol, 87%). Mixture of diastereomers: ^{1}H NMR (400 MHz, $CDCl_3$) δ 7.44 (d, *J*=2.8, 1H), 7.42 (d, *J*=2.5, 1H), 4.69 – 4.61 (m, 2H), 2.85 – 2.79 (m, 2H), 2.74 – 2.64 (m, 3H), 2.64 – 2.50 (m, 3H), 2.35 – 2.23 (m, 2H), 2.20 (s, 3H), 2.19 (s, 3H), 2.16 (s, 3H), 2.15 (s, 3H), 2.08 (s, 4H), 2.07 (s, 4H), 2.03 (t, *J*=2.5, 1H), 2.00 – 1.88 (m, 2H), 1.78 (t, *J*=2.5, 1H), 1.53 (s, 3H), 1.53 (s, 3H). ^{13}C NMR (101 MHz, $CDCl_3$) δ = 175.3, 175.1, 173.7, 173.6, 172.3, 145.6, 145.5, 144.1, 144.0, 122.1, 122.1, 121.7, 121.6, 78.0, 77.9, 77.7, 77.2, 72.0, 71.7, 50.2, 50.0, 29.4, 29.2, 25.3, 23.8, 23.8, 21.7, 21.7, 20.3, 20.2, 12.2, 11.9, 11.8, 11.3. HRMS (negative): ($C_{19}H_{22}NO_5^-$) 344.1493 (found, M-H), 344.1504 (calc.).

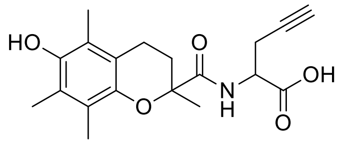

11 ("TX-PG-NHS") *2,5-dioxopyrrolidin-1-yl 2-(6-hydroxy-2,5,7,8-tetramethylchroman-2-carboxamido) pent-4-ynoate*: To **10** (266 mg, 0.771 mmol) and N-hydroxysuccinimide (NHS) (89 mg, 0.771 mmol), in

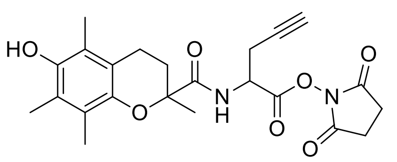

1.0 mL anhydrous 1,4-dioxane, N,N'-dicyclohexyl carbodiimide (DCC) (159 mg, 0.771 mmol) was added at 0°C. After stirring at ambient temperature overnight, the mixture was cooled to 10°C and the precipitate was filtered. The filtrate was evaporated in vacuo. The crude was purified by flash column chromatography (SiO_2, Pentane/EtOAc: 100/0 to 0/100) and the product was isolated as white solid. Yield: 172 mg (0.385 mmol, 50%). Mp: 60 - 61°C. Mixture of diastereomers: ^{1}H NMR (400 MHz, $CDCl_3$) δ 7.30 (s, 1H), 7.28 (s, 1H), 5.08 – 4.98 (m, 2H), 4.45 (br, 1H), 4.43 (br, 1H), 2.97 – 2.72 (m, 4H), 2.84 (s, 4H), 2.78 (s, 4H), 2.69 – 2.53 (m, 4H), 2.40 – 2.29 (m, 2H), 2.19 (s, 6H), 2.16 (s, 3H), 2.15 – 2.10 (m, 4H), 2.08 (s, 3H), 2.05 (s, 3H), 1.98 – 1.86 (m, 3H), 1.55 (s, 3H), 1.54 (s, 3H). ^{13}C NMR (101 MHz, $CDCl_3$) δ = 174.5, 174.3, 168.3, 168.0, 166.1, 165.8, 145.7, 145.5, 144.1, 144.1, 122.1, 122.1, 121.4, 121.4, 119.0, 118.9, 78.0, 76.7, 76.3, 72.7, 72.3, 48.6, 29.3, 29.3, 25.5, 25.5, 24.1, 24.0, 22.5, 22.4, 20.3, 20.3, 12.2, 12.2, 11.9, 11.8, 11.3, 11.2. HRMS (negative): ($C_{23}H_{25}N_2O_7^-$) 441.1662 (found, M-H), 441.1667 (calc.).

16 ("NPA-RhodamineB") *(S)-Rhodamine B 4-(4-((1-Carboxy -2-(4-nitrophenyl)ethyl)amino)-4-oxo-butyryl) piperazine amide*: Rhodamine B 4-(3-Carboxypropionyl) piperazine amide was prepared as described previously in literature.[1] To rhodamine B 4-(3-Carboxy-propionyl) piperazine amide (200 mg, 0.3 mmol) and N-hydroxysuccinimide (45 mg, 0.4 mmol), in 20 mL anhydrous DMF, N,N'-dicyclohexylcarbodiimide (79 mg, 0.4 mmol) was added. After stirring for 12 h under inert conditions

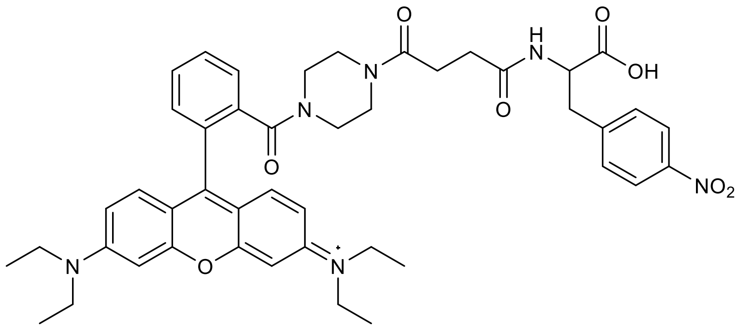

at room temperature, the precipitated dicyclohexylurea (DCU) was removed by filtration. RhodamineB-NHS (**14**) was used for coupling without further purification. To RhodamineB-NHS (**14**), in 30 mL DMF, (S)-Nitrophenylalanin(NPA) (344 mg, 1.6 mmol) was added, followed by 50 µL triethylamine. After stirring for 12 h at room temperature under inert conditions the solvent was evaporated in vacuo. The product (NPA-RhodamineB (**16**)) was purified by column chromatography (SiO_2, DCM/Methanol: 10/1) and isolated as pink solid. Yield: 210.2 mg (0.262 mmol, 80%). ^{1}H NMR (400 MHz, CD_3OD) δ 1.28-1.32 (t, 12H, J=6.8), 2.41 (br s, 2H), 2.47 (br s, 2H), 3.01 (dd, 1H, J=14.1, 5.1),3.34 (m, 10H), 3.66-3.71 (q, 8H, J=7.2), 4.48(dd, J=11.0, 6.2, 1H), 6.96-6.97 (br s, 2H), 7.06 (d, 2H, J=2.0), 7.24-7.27 (d, 2H, J=9.6), 7.41 (d, 2H, J=7.3), 7.49-7.50 (m, 1H), 7.68-7.69 (m, 1H), 7.74-7.75 (m, 2H), 8.02 (d, J=8.2, 2H),. ^{13}C NMR(101 MHz, CD3OD) δ = 175.9, 172.4, 171.2, 168.1, 157.8, 155.8, 155.5, 146.8, 146.5, 135.1, 131.7, 131.0, 129.9, 127.5, 122.7, 114.0, 113.4, 96.0, 55.4, 48.3, 48.0, 47.8, 47.6, 47.4, 47.2, 47.0, 45.5, 38.0, 30.4, 27.9, 11.5. HRMS: ($C_{45}H_{51}N_6O_8^+$) 803.3987 (found) 803,3763 (calc.).

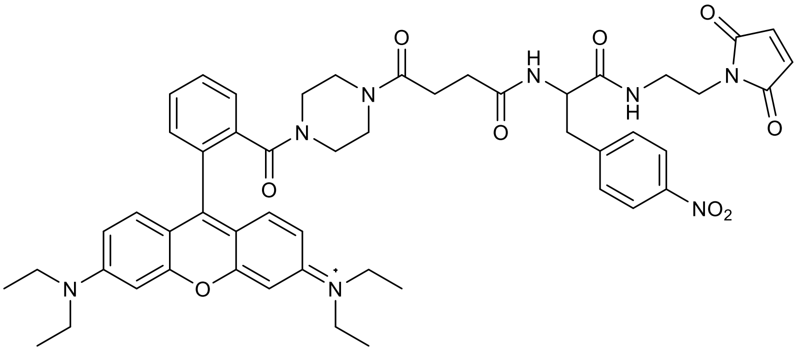

17 and **20** (**"NPA-RhodamineB-NHS"** and **"NPA-RhodamineB-Maleimide")** *(S)-Rhodamine B 4-(4-((1-oxo-1-(2-(maleimide)ethylamino)-3-(4-nitro-phenyl))isopropylamino) - 4-oxobutyryl) piperazine amide:* To (S)-rhodamine B 4-4-((1-Carboxy -2-(4-nitrophenyl) ethyl) amino) -4-oxobutyryl) piperazine amide (**16**) (80.4 mg, 0.1 mmol) and N-hydroxysuccinimide (13.6 mg, 0.12mmol), in 20mL anhydrous DMF, N,N'-dicyclohexylcarbodi-imide (23.7 mg, 0.12 mmol) was added. After stirring for 12 h under inert conditions at room temperature, the precipitated dicyclohexylurea (DCU) was removed by filtration. The resulting RhoB-NPA-NHS was used further without purification. To the crude RhoB-NPA-NHS mixture and N-(2-Aminoethyl)maleimide trifluoro-acetate salt (25.4 mg, 0.1 mmol), in 5 mL DMF, 17 µL triethylamine was added. After stirring for 12 h at room temperature under inert conditions the solvent was removed by evaporation in vacuo. The product (RhoB- NPA-maleimide) was purified by column chromatography (SiO_2, DCM/Methanol: 15/1) and isolated as pink solid. Yield: 55.5 mg (0.06 mmol, 60%). HRMS: ($C_{51}H_{57}N_8O_9^+$) 925.53 (found), 925.42 (calc.).

("RhodamineB-Maleimide") *(S)-Rhodamine B 4-(4-(2-(maleimide)ethylamino)-4-oxobutyryl) piper-azine amide :* Rhodamine B 4-(3-Carboxypropionyl) piperazine amide was prepared as described previously in literature.[1] To rhodamine B 4-(3-Carboxypropionyl)piperazine amide (200 mg, 0.3 mmol) and N-hydroxysuccinimide (45.2 mg, 0.4mmol), in 20mL anhydrous DMF, N,N'-dicyclohexylcarbodiimide (79.1 mg, 0.4 mmol) was added. After stirring for 12 h at room temperature under inert conditions, the precipitated dicyclohexylurea (DCU) was removed by filtration. RhodamineB-NHS (**14**) was used without further purification. To the crude RhodamineB-NHS (**14**) and N-(2-Aminoethyl)maleimide trifluoroacetate salt (76.2 mg, 0.3 mmol), in 10mL DMF, 50µL triethylamine was added. After stirring for 12 h at room temperature under inert conditions the solvent was removed by evaporation in vacuo. The product (RhodamineB-maleimide) was purified by column chromatography (SiO_2, DCM/Methanol: 20/1) and isolated as pink solid. Yield: 154.9 mg (0.21 mmol, 70%). ^{1}H NMR (400 MHz, $CDCl_3$) δ 7.74 – 7.60 (m, 2H), 7.53 (d, *J* = 6.9 Hz, 1H), 7.34 – 7.27 (m, 1H), 7.22 (t, *J* = 10.4 Hz, 2H), 7.03 (d, *J* = 7.5 Hz, 1H), 6.93 – 6.81 (m, 1H), 6.75 (d, *J* = 6.3 Hz, 1H), 6.70 (s, 1H), 6.66 (s, 1H), 6.54 (s, 1H), 3.60 (tq, *J* = 14.8, 7.6 Hz, 10H), 3.43 (d, *J* = 24.8 Hz, 10H), 2.66 – 2.60 (m, 2H), 2.42 (t, *J* = 6.8 Hz, 2H), 1.31 (t, *J* = 6.9 Hz, 12H). ^{13}C NMR (101 MHz, $CDCl_3$) δ 172.90 ,171.02 , 157.69, 155.67, 135.13, 134.13, 132.14, 130.15, 130.01, 127.57, 113.83, 95.95, 77.21, 46.06, 45.39, 41.71, 41.12, 38.47, 37.50, 28.69, 12.55. HRMS: ($C_{42}H_{49}N_6O_6^+$) 733.41 (found), 733.37 (calc.).

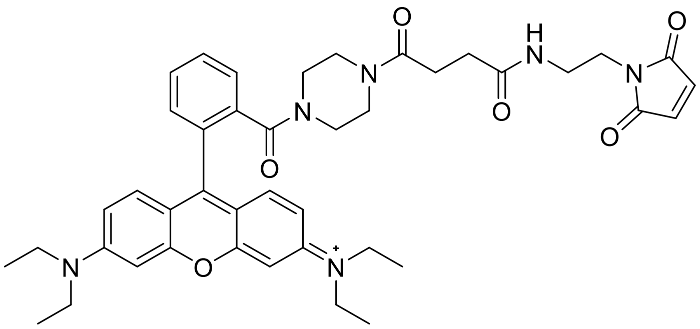

23 ("BOC-NPA-Maleimide") *tert-butyl (1-((2-(2,5-dioxo-2,5-dihydro-1H-pyrrol-1-yl)ethyl)amino)-3-(4-nitrophenyl)-1-oxopropan-2-yl)carbamate:* To **22** (25 mg, 80 µmol) and **19** (40 mg, 157 µmol) in 1 mL anhydrous DMF was added HBTU reagent (91 mg, 240 µmol) and Et_3N (55 uL, 395 µmol). The mixture was stirred overnight under Argon and protected from light. The reaction mixture was diluted with water and extracted three times with diethyl ether. The combined organic extracts were washed with 1:1 water:brine and with brine, dried on $MgSO_4$, filtrated and the solvent was evaporated in vacuo. The product was purified by column chromatography (SiO_2, DCM/Methanol: 20/1) and isolated as white solid. Yield: 30 mg (69 µmol, 87%). ^{1}H NMR (400 MHz, $CDCl_3$) δ 8.15 (d, *J* = 8.5 Hz, 2H), 7.38 (d, *J* = 8.5 Hz, 2H), 6.69 (s, 2H), 4.36 (d, *J* = 7.0 Hz, 1H), 3.72 – 3.60 (m, 2H), 3.54 – 3.42 (m, 1H), 3.42 – 3.31 (m, 2H), 3.29 – 3.20 (m, 1H), 3.09 – 2.98 (m, 2H), 1.39 (s, 9H). ^{13}C NMR (101 MHz, $CDCl_3$) δ 170.86, 170.75, 144.76, 134.18, 130.24, 123.68, 53.40, 39.18, 38.12, 37.27, 28.21. LCMS (positive): 333.0990 (found M-BOC+H), calc. 333.1194, 377.0884 (found M-tBoc+2Na-H), calc. 377.0833.

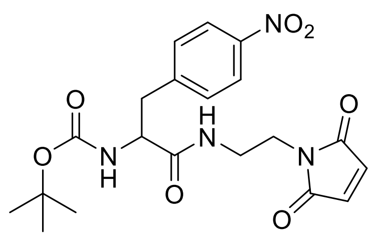

24 ("NPA-Maleimide") *2-amino-N-(2-(2,5-dioxo-2,5-dihydro-1H-pyrrol-1-yl)ethyl)-3-(4-nitrophenyl) propanamide:* **23** (1.5 mg, 3.45 µmol) was dissolved in 1 mL DCM at 0 °C. Trifluoroacetic acid (TFA, 1 mL) was added dropwise and the mixture was stirred overnight at room temperature under argon. DCM was evaporated in vacuo and the remaining TFA solution was precipitated in cold diethyl ether. The resulting suspension was spun down and the supernatant was removed. The product was obtained as the TFA salt and used immediately in the next step. ^{1}H NMR (400 MHz, D_2O) δ 8.10 (d, 2H), 7.37 (d, *J* = 8.6 Hz, 2H), 6.71 (s, 2H), 4.09 (t, *J* = 7.5 Hz, 1H), 3.48 – 3.39 (m, 2H), 3.31 – 3.21 (m, 2H), 3.18 – 3.10 (m, 2H). LCMS (positive): 333.0990 (found M+H), calc. 333.1194, 377.0884 (found M+2Na-H), calc. 377.0832.

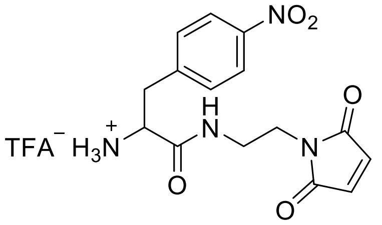

25 ("ATTO647N-NPA-Maleimide"): To ATTO647N NHS ester (1 mg, 1.18 µmol) and freshly deprotected **24** (1.15 mg, 3.45 µmol) dissolved in 500 µL anhydrous DMF was added 1.5 µL Et_3N (10.8 µmol). The reaction mixture was protected with Argon and placed in a shaker at 20 °C overnight. The solvent was removed under high vacuum and the crude mixture was dissolved in 1 mL methanol and filtered (syringe filter, 0.22 µm). The solution was purified by preparative HPLC. Yield: 414 µg (432 nmol, 37%). ^{1}H NMR (500 MHz, CD_3OD) δ 8.14 – 8.07 (m, 2H), 7.69 – 7.65 (m, 2H), 7.49 – 7.41 (m, 4H), 6.86 (dd, *J* = 16.6, 3.6 Hz, 1H), 6.79 (d, *J* = 2.7 Hz, 2H), 6.73 (s (br), 1H), 3.79 – 3.72 (m, 2H), 3.67 – 3.48 (m, 10H), 3.25 – 3.16 (m, 8H), 2.81 (s, 2H), 2.77 (s, 1H), 2.75 (s, 1H), 2.56 (s, 2H), 1.84 (dd, *J* = 16.6, 9.8 Hz, 7H), 1.75 (s, 2H), 1.60 (d, *J* = 1.4

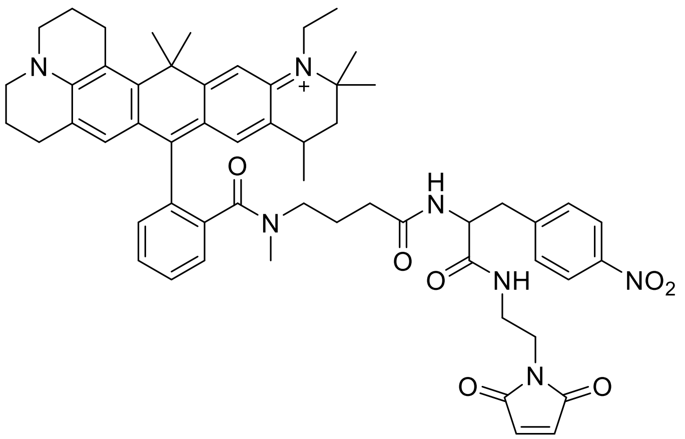

Hz, 3H), 1.47 – 1.41 (m, 8H), 1.00 (dd, J = 14.7, 6.3 Hz, 4H), 0.93 (t, J = 7.5 Hz, 1H), 0.91 – 0.86 (m, 3H). HPLC was performed with Shimadzu LC-10AD VP over Agilent SB C18 column (4.6 mm x 75 mm, particle size 3.5 µm) with gradient A/B: MeCN/H_2O + 0.1 % v/v TFA 0:100 → 100:0 in 60 min, column oven 30 °C. t_R = 36.8 min, 37.6 min for two ATTO647N isomers. Peaks were collected separately and after LCMS confirmed equal mass the fractions were combined for NMR.

28 ("NPA-KK114"): The dye KK114 (**27**) (10 mg, 0.011 mmol),[2] N-hydroxysuccinimide (13 mg, 0.11 mmol), HATU reagent (12 mg, 0.032 mmol), and Et_3N (25 µL, 0.17 mmol) were combined in anhydrous DMF (0.5 mL). The solution was stirred at room temperature overnight under an argon atmosphere. L-4-Nitrophenylalanine (**15**) (20 mg, 0.09 mmol) in DMF (1 mL) and more Et_3N (25 µL, 0.17 mmol) was introduced, and stirred at room temperature overnight under an argon atmosphere. The solvent was evaporated in vacuo. The crude product was dissolved in water (few mL) and filtered

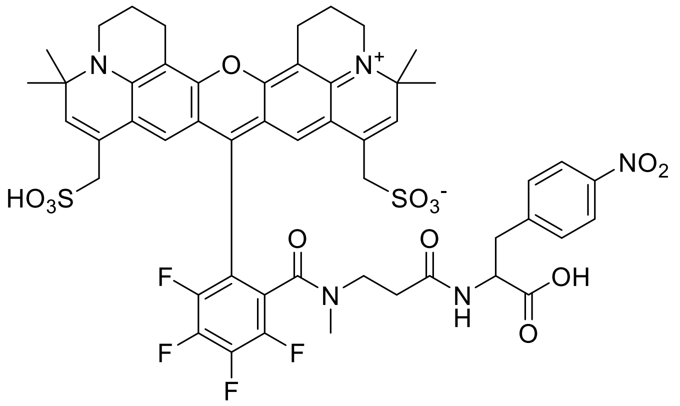

(Rotilabo syringe filters, 0.22 µm). The solution was concentrated to 1 mL and purified by preparative HPLC. Yield: 4.5 mg (4.2 µmol, 37%). ^{1}H-NMR (400 MHz, Acetone-d6; mixture of diasterioisomers: only broad unresolved signals were recorded) δ = 1.31/1.35/1.37 (s×3, 12 H, CH_3), 1.54 (m, 2 H, CH_2), 2.04 (m, 4 H, CH_2×2), 2.80 (m, 2 H, CH_2CO), 3.00 (s, 3 H, NCH_3), 3.18 (m, 2 H, CH_2), 3.30 (m, 2 H, CH_2), 3.52 – 3.64 (m, 2 H, CH_2Ph, m, 4 H, CH_2SO_3), 3.70 (m, 4 H, CH_2N), 4.50 (m, 1H, $CHCO_2H$), 5.85 (m, 2 H), 7.30 – 7.50 (m, 4 H), 8.00 – 8.20 (m, 2 H) ppm. MS (ESI) *m/z* (positive): found 1080 (100%) [M+H]. HRMS (negative): ($C_{51}H_{49}F_4N_5O_{13}S_2$): 1078.2612 (found M–H), 1078.2626 (calc.). HPLC was performed with Shimadzu LC-10AD VP over Agilent SB C18 column (4.6 x 75mm, particle size 3.5 µm) with gradient A/B: MeCN/H2O+50 mM TEAB; 0:100→60:40 in 20 min, to 100:0 at 25 min.); t_R = 13.8 min.

31 ("β-Ala-NPA-KK114-OH", *the modified dye conjugate with the linking site elongated by one β-alanine* moiety): The elongation of the linking site was performed as described for a sulfocysteine-decorated carbopyronine dye.[3] Such an elongation provided a very high hydrolytic stability of the corresponding NHS ester (obtained from the resulting dye conjugate). That proved to be true for the NHS ester of NPA-KK114 as well. The preliminary experiments showed that the unmodified NHS ester has an insufficient stability and is therefore unsuitable for antibody conjugation.

The modification was performed as follows: compound NPA-KK114 (**28**) (2.2 mg, 2 µmol) was reacted

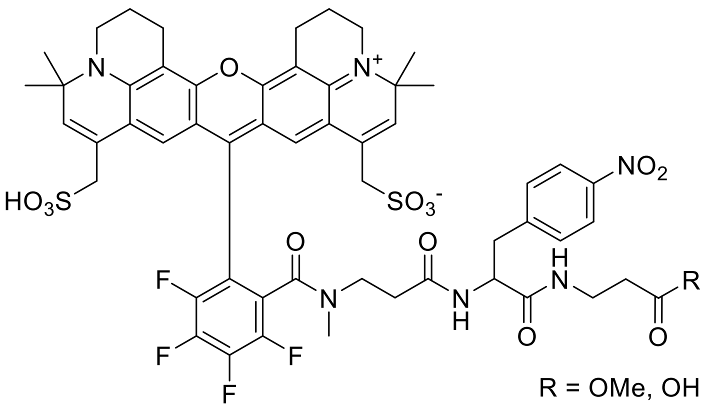

with β-alanine methyl ester hydrochloride (**29**) (2 mg, 13 µmol) in the presence of HATU reagent (4 mg, 11 µmol) and Et_3N (4 µL, 28 µmol) in DMF (0.5 mL) at room temperature under an argon atmosphere. As the reaction was complete (in ca. 2 h, as established by HPLC), the solution was quenched with water (1 mL) and loaded onto a column with reverse-phase silica gel (Macherey-Nagel, Polygoprep 60-50 C18, 2 g) and water containing 0.1 % v/v TFA. In the course of the elution, MeCN was gradually added to the mobile phase up to the ratio of 1:1. The pure fractions were pooled, filtered (Rotilabo® syringe filters, 0.22 µm), concentrated, and freeze-dried to afford ca. 2 mg of a crude methyl ester β-Ala-NPA-KK114-OMe (**30**). Without further treatment, the intermediate was saponificated by stirring overnight at room temperature in a dilute alkaline solution made of water (3 mL) and 1 M aq. NaOH (0.15 mL). The solution was acidified with an excess of TFA (10 µL) and the product isolated by column chromatography (over 2 g of RP-SIO_2) exactly as described above for the starting methyl ester. Filtration and evaporation of the pure fractions of the acid β-Ala-NPA-KK114-OH (**31**) afforded a crystalline blue solid, well soluble in basified water or DMF, sparingly soluble in acetone. Yield: 1.4 mg (1.2 µmol, 60% over two steps). MS (ESI) *m/z* (negative): found 1149 (100%) [M–H]. HRMS: ($C_{54}H_{54}F_4N_6O_{14}S_2$): 1149.2989 (found M–H), 1149.2996 (calc.). The HPLC analyses were performed by means of Knauer Smartline semi-preparative high pressure gradient system with two pumps, mixing chamber, column thermostat 4000 (25 °C), and an UV detector 2550 (gradient A/B: MeCN/H_2O+0.1 % v/v TFA; 10:90→100:0 in 20 min): t_R = 9.4 min.

33 (β-Ala-NPA-KK114-NHS, *The active ester of the modified dye conjugate):* The NHS ester was obtained as follows: N,N'-disuccinimidyl carbonate (**32**) (DSC reagent, 1.5 mg, 6 µmol) was added to a solution of acid β-Ala-NPA-KK114-OH (**31**) (1.2 mg, 1 µmol) in DMF (0.30 mL) containing Et_3N (3 µL, 20 µmol) in a 2.5-mL vial with a magnetic stirring bar. The vial was flushed with argon, sealed, and the solution stirred until the reaction was complete (ca. 30 min, as established by HPLC). The solution

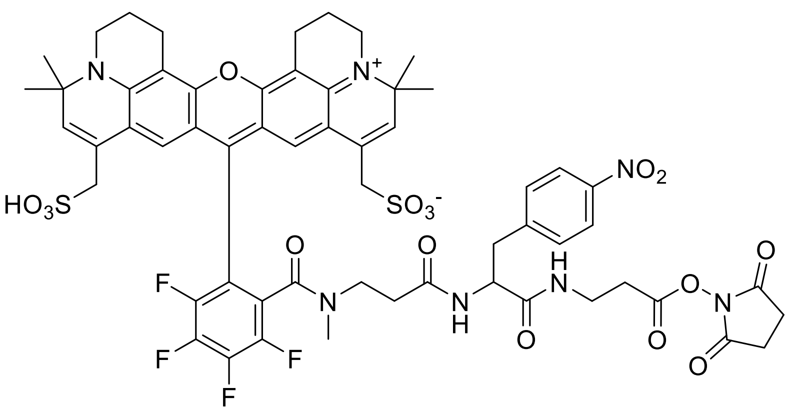

was thoroughly evaporated to dryness straight from the vial in a high vacuum (for evaporation a small hole in the cap was pierced and the vial was placed inside a flask). The residue was sonificated for 3 min with ethyl acetate (2 mL) and the suspension centrifuged. The mother liquor, which contained DSC and NHS, was separated. The precipitate was dried from the residual solvent and re-dissolved in DMF (0.3 mL). The solution was divided in to 6 aliquots (50 µL, each containing ca. 200 µg of the active ester) that were carefully

evaporated in vacuo, as described above, flushed with argon, and stored at –20°C. The dried aliquots proved sufficiently pure (98%, HPLC) and were used for conjugation to antibodies[4,5] as they were. MS (ESI) m/z (negative, %) = 1246 (100%) [M–H]; HRMS (negative): ($C_{58}H_{57}F_4N_7O_{16}S_2$): 1246.3175 (found M–H), 1246.3167 (calc.); HRMS (positive): ($C_{58}H_{57}F_4N_7O_{16}S_2$): 1286.2848 (found M+K), 1286.2871 (calc.). HPLC (A/B: MeCN/H_2O + 0.1 % v/v TFA; 10:90→100:0 in 20 min): t_R = 10.2 min.

References

1. Nguyen, T. & Francis, M. B. Practical synthetic route to functionalized rhodamine dyes. *Org. Lett.* **5,** 3245–8 (2003).

2. Kolmakov, K. *et al.* Red-emitting rhodamine dyes for fluorescence microscopy and nanoscopy. *Chemistry* **16,** 158–66 (2010).

3. Kolmakov, K. *et al.* A Versatile Route to Red-Emitting Carbopyronine Dyes for Optical Microscopy and Nanoscopy. *European J. Org. Chem.* **2010,** 3593–3610 (2010).

4. Wurm, C. A., Neumann, D., Schmidt, R., Egner, A. & Jakobs, S. Sample preparation for STED microscopy. *Methods Mol. Biol.* **591,** 185–99 (2010).

5. For the standard conjugation protocol see also also www.abberior.com / references / protocols / antibody labeling protocols

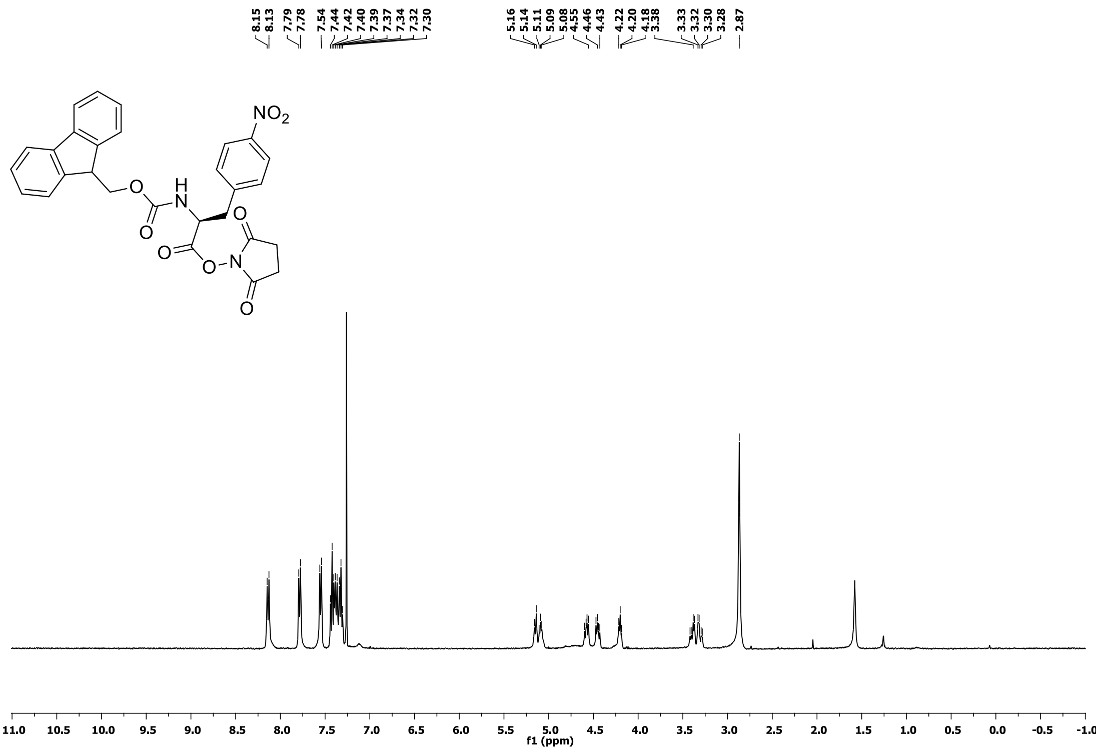

Supplementary Figure 16. ^{1}H-NMR of NPA-NHS (**2**).

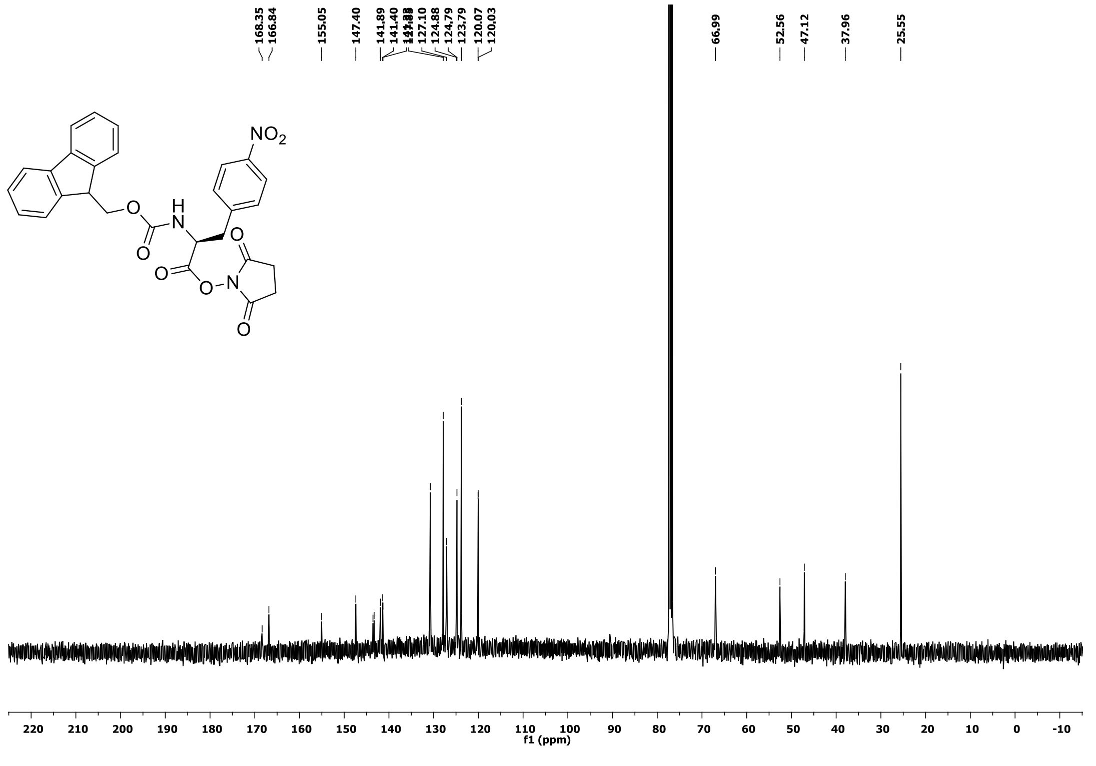

Supplementary Figure 17. ^{13}C-NMR of NPA-NHS **(2)**.

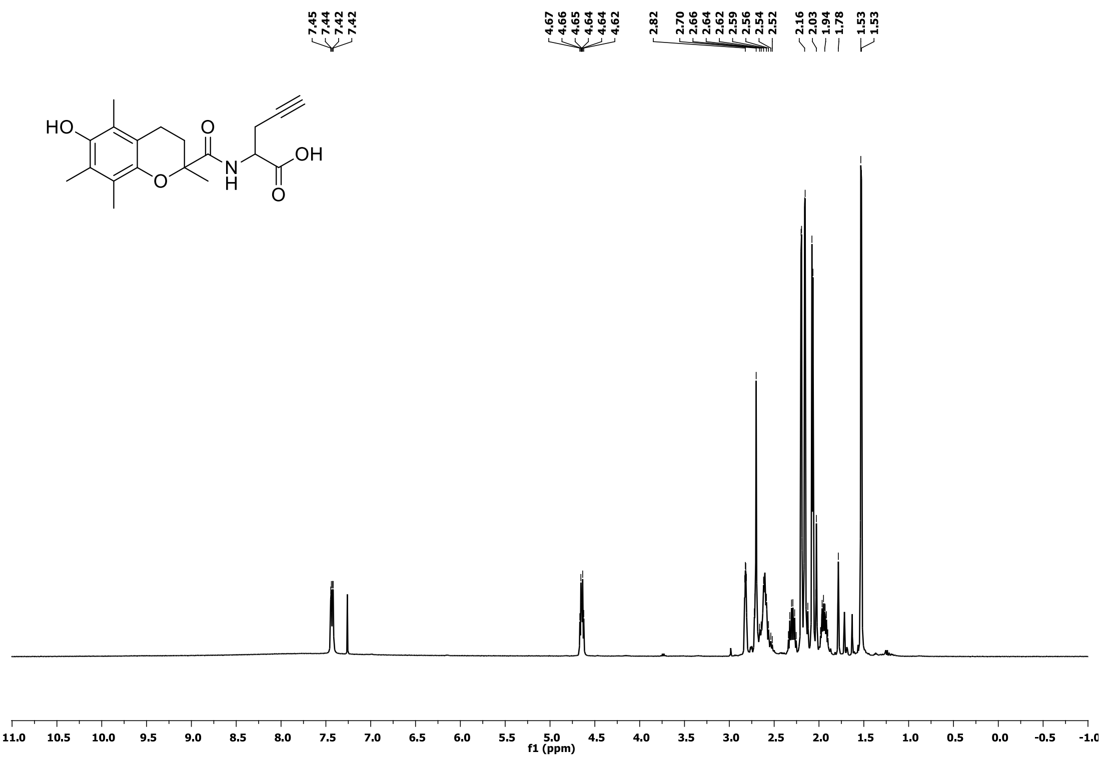

Supplementary Figure 17. ^{1}H-NMR of TX-PG (**10**).

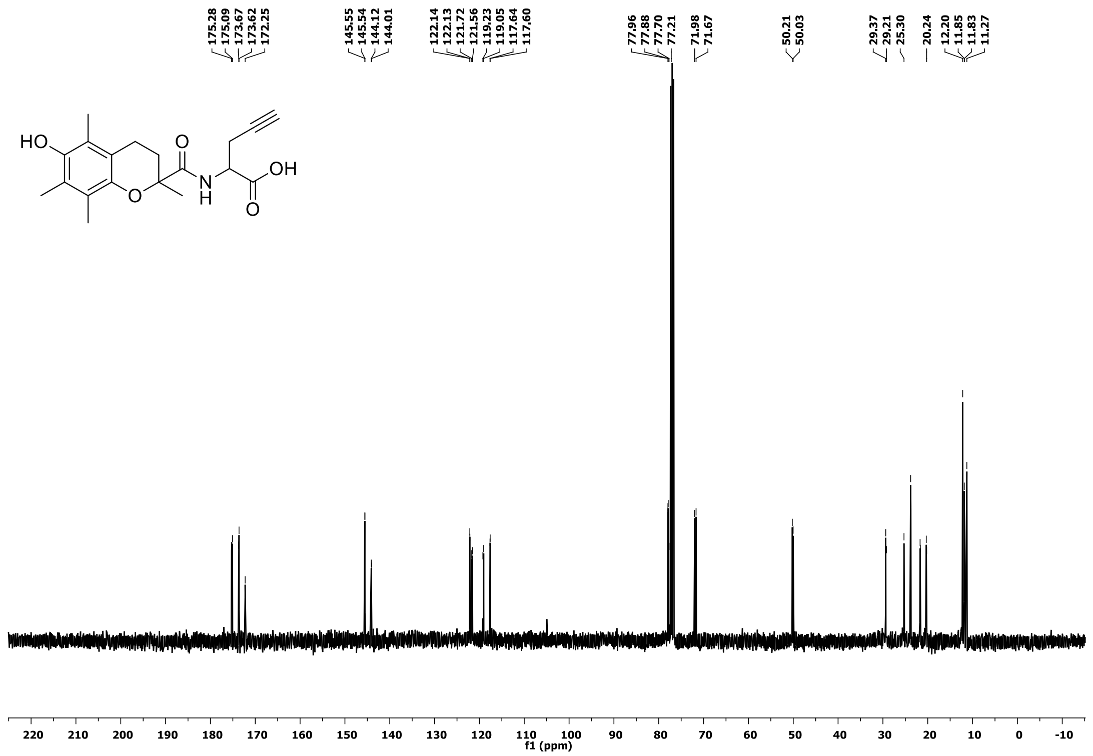

Supplementary Figure 18. ^{13}C-NMR of TX-PG (**10**).

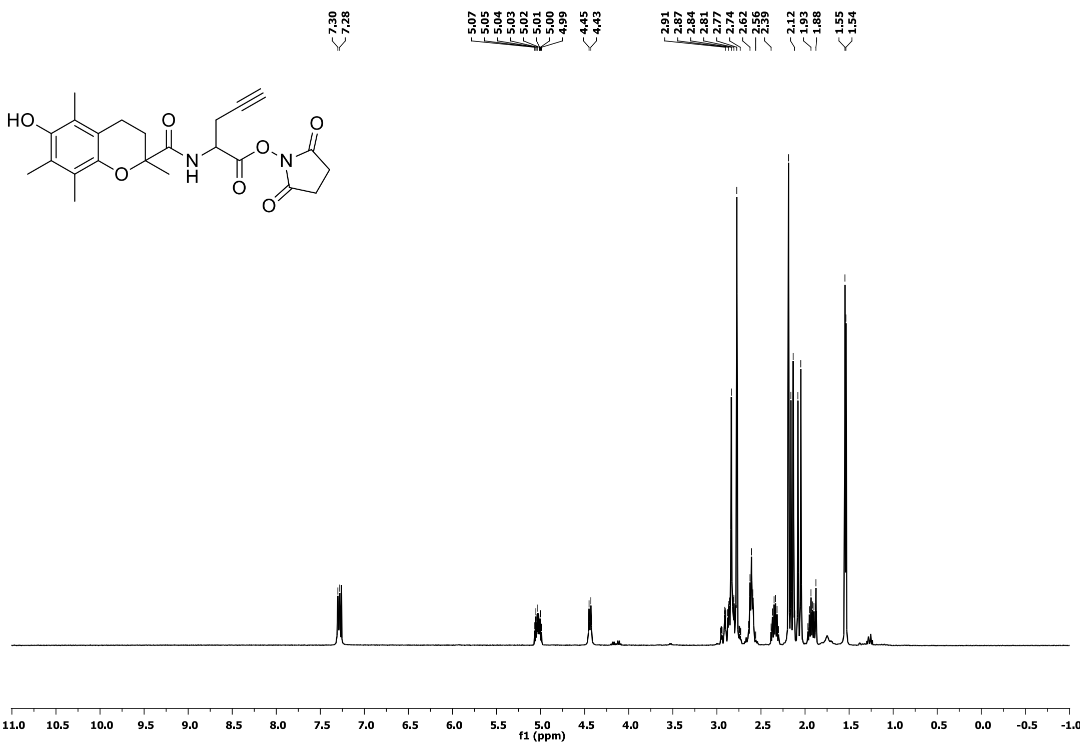

Supplementary Figure 19. ^{1}H-NMR of TX-PG-NHS (**11**).

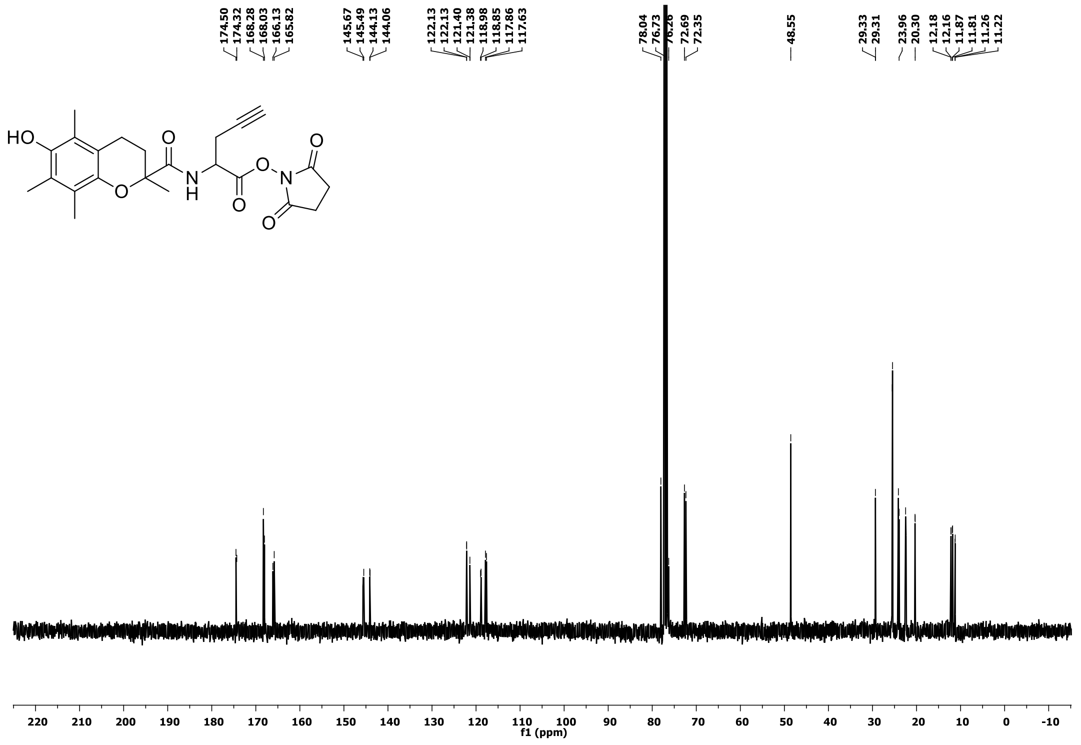

Supplementary Figure 21. ^{13}C-NMR of TX-PG-NHS (**11**).

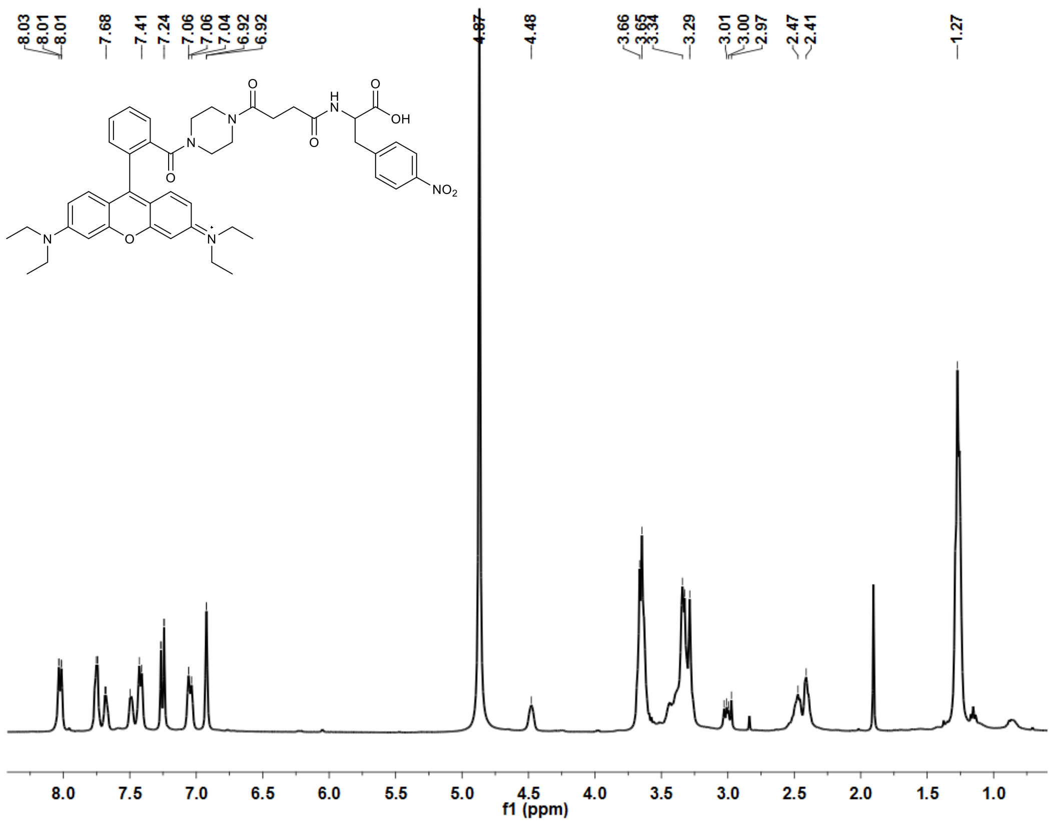

Supplementary Figure 22. ^{1}H-NMR of NPA-RhodamineB (**16**).

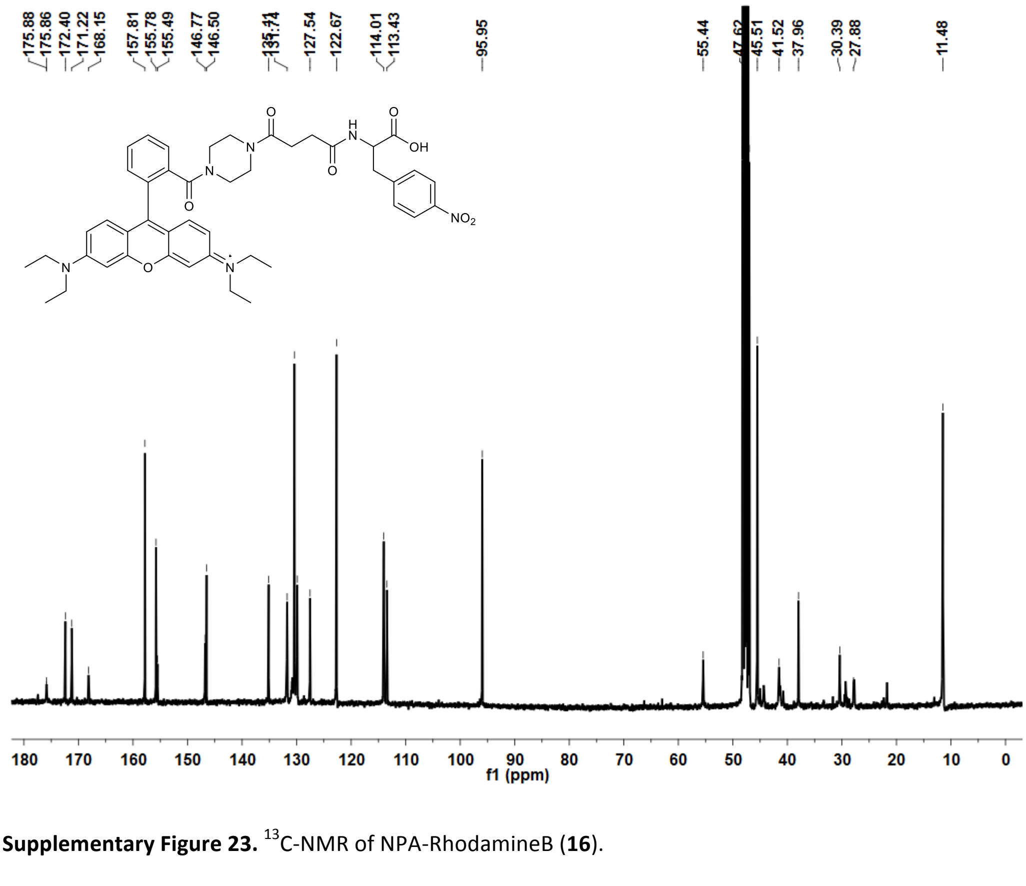

Supplementary Figure 23. ^{13}C-NMR of NPA-RhodamineB (**16**).

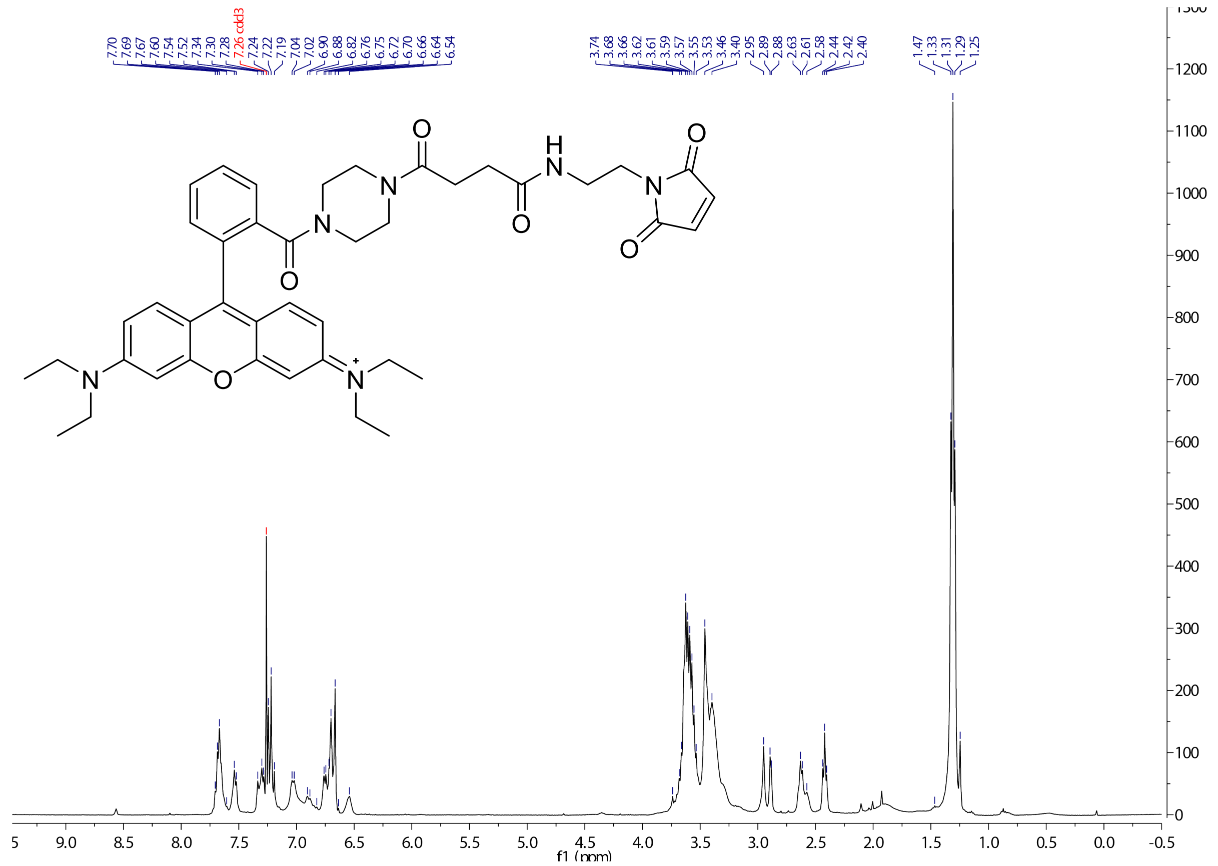

Supplementary Figure 24. ^{1}H-NMR of RhodamineB-Maleimide.

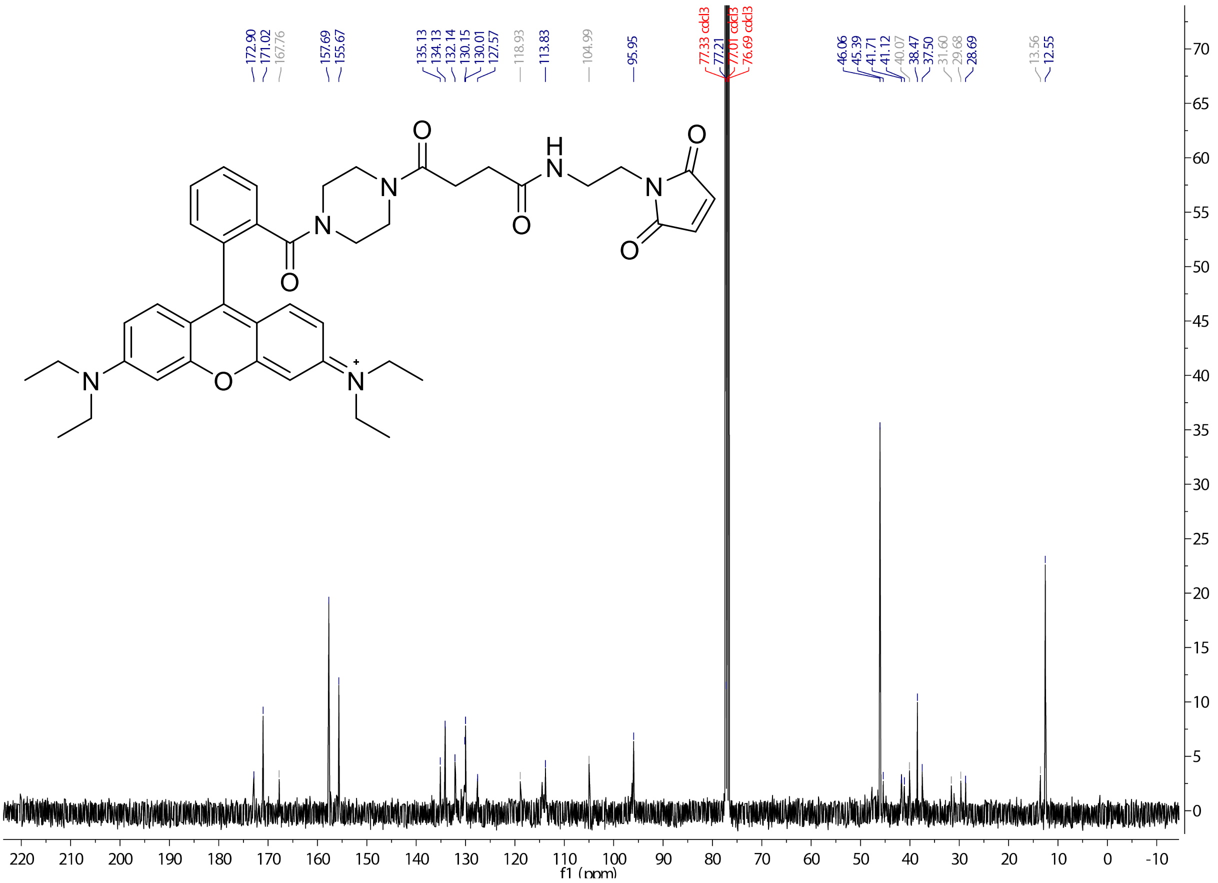

Supplementary Figure 25. ^{13}C-NMR of RhodamineB-Maleimide.

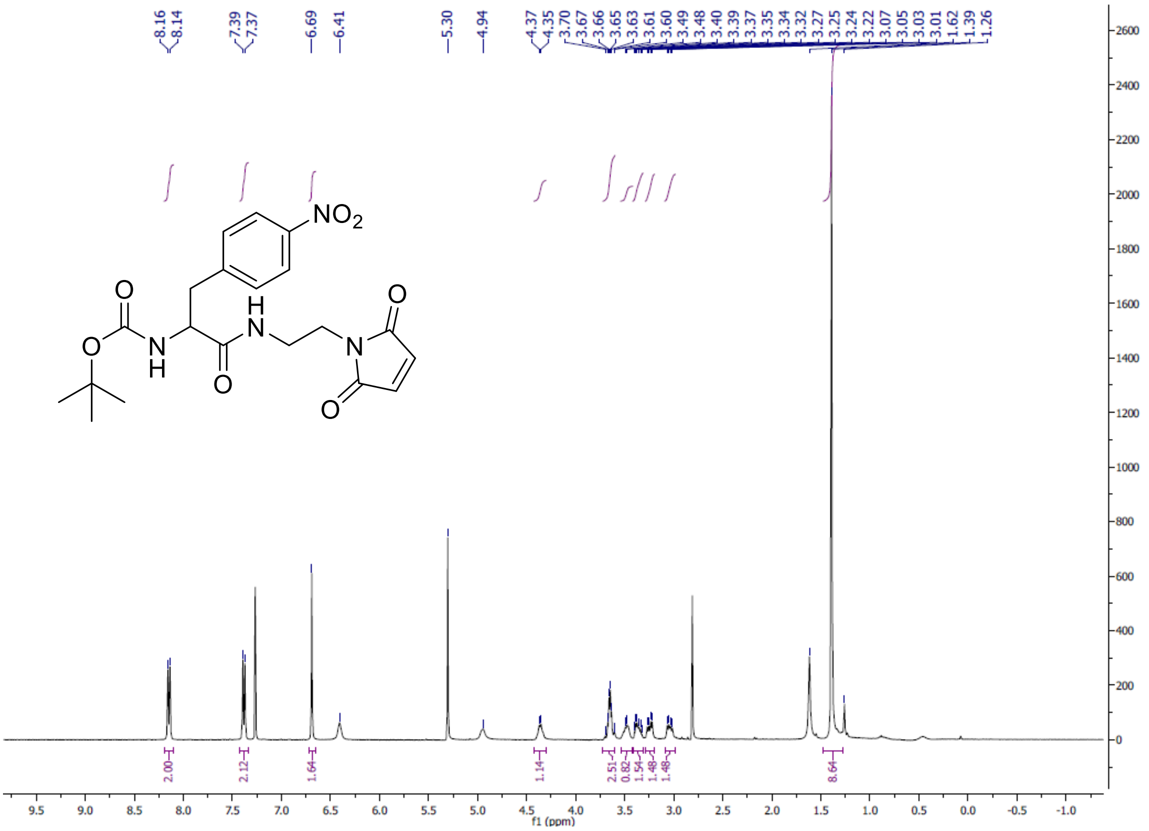

Supplementary Figure 26. ^{1}H-NMR of BOC-NPA-Maleimide (**23**).

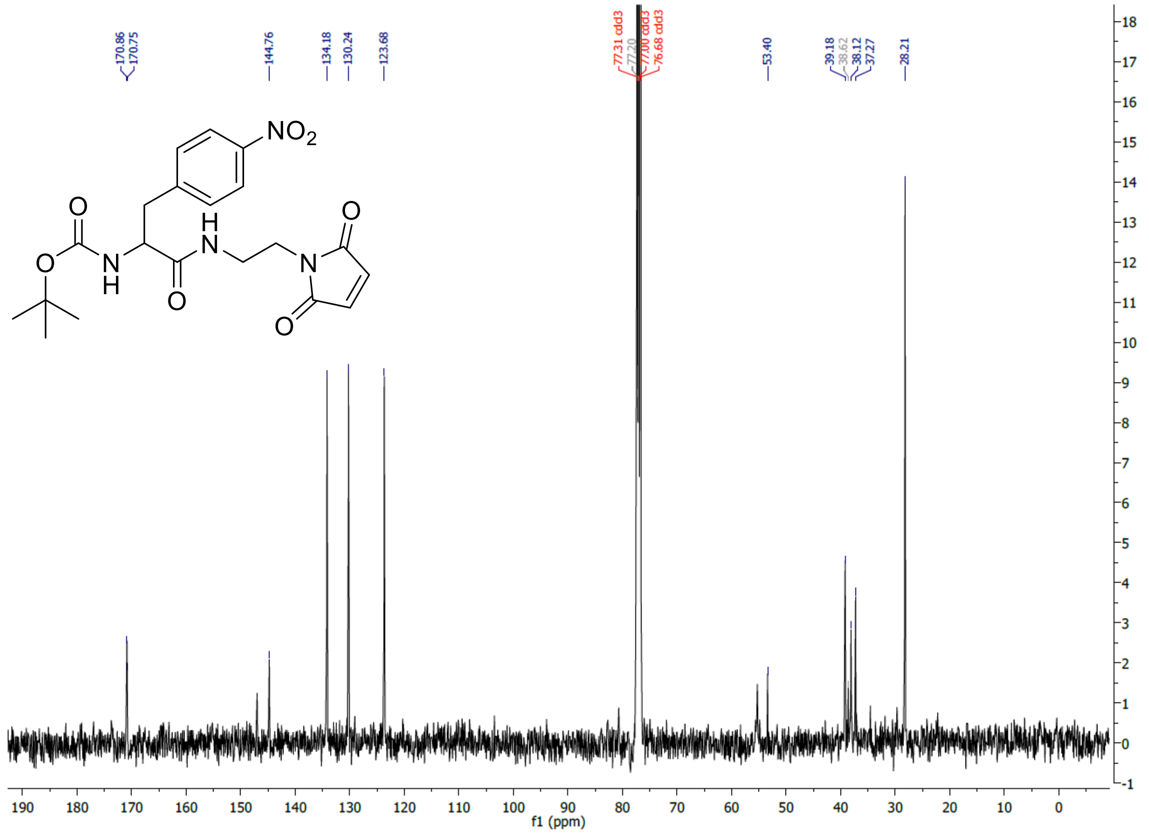

Supplementary Figure 27. ^{13}C-NMR of BOC-NPA-Maleimide (**23**).

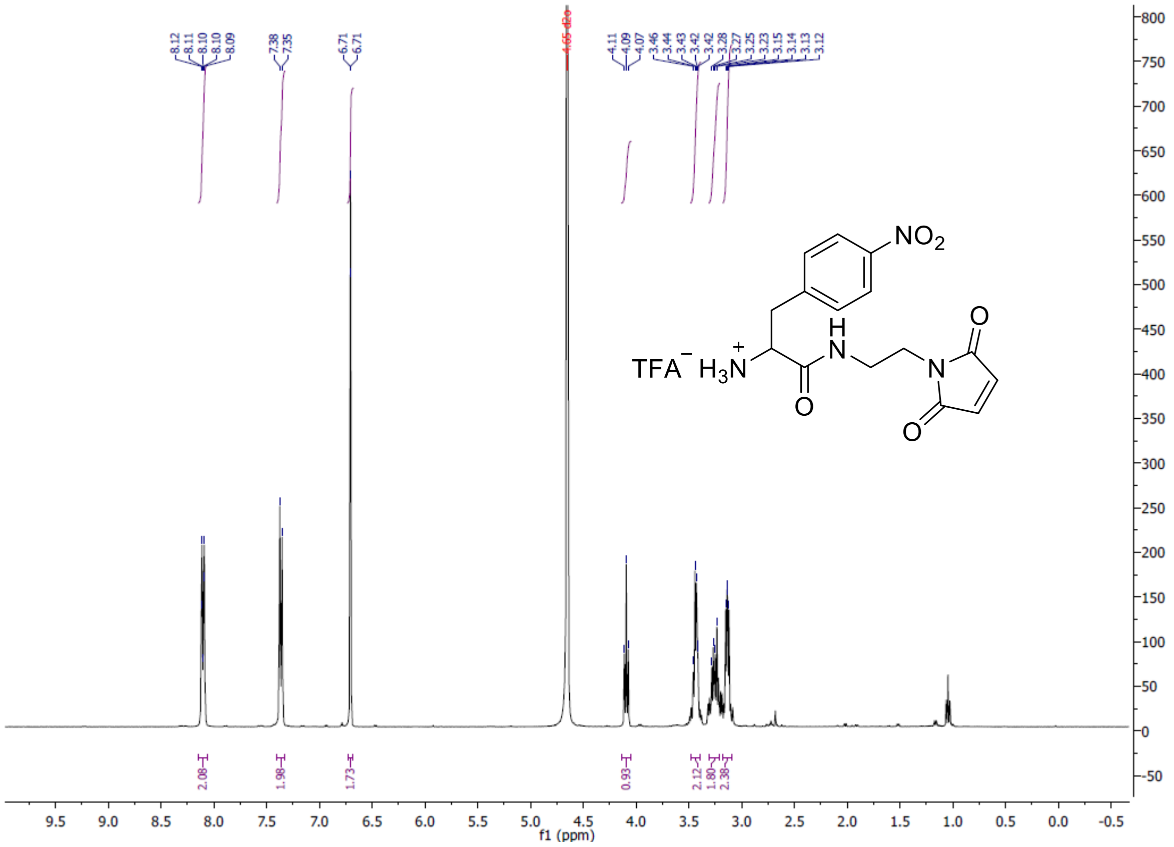

Supplementary Figure 28. ^{1}H-NMR of BOC-NPA-Maleimide (**24**).

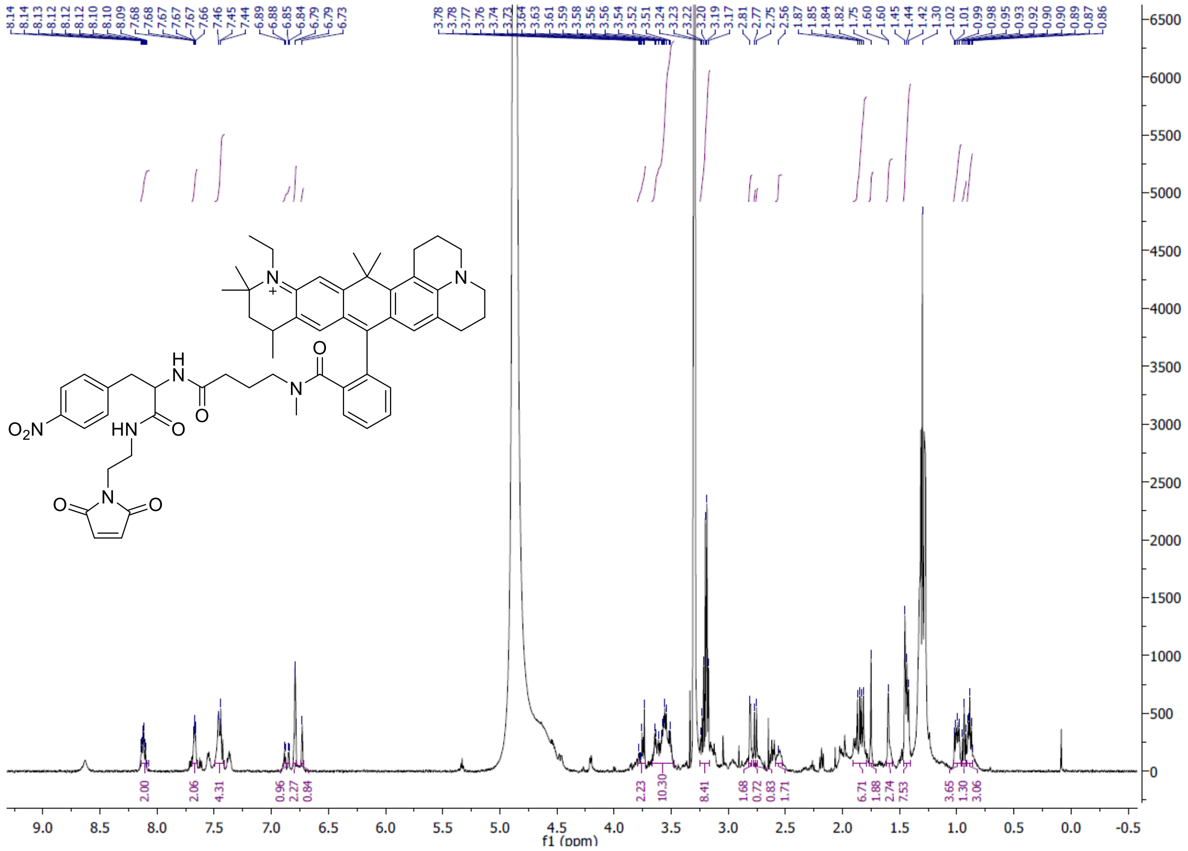

Supplementary Figure 29. ^{1}H-NMR of ATTO647N-NPA-Maleimide (**24**).

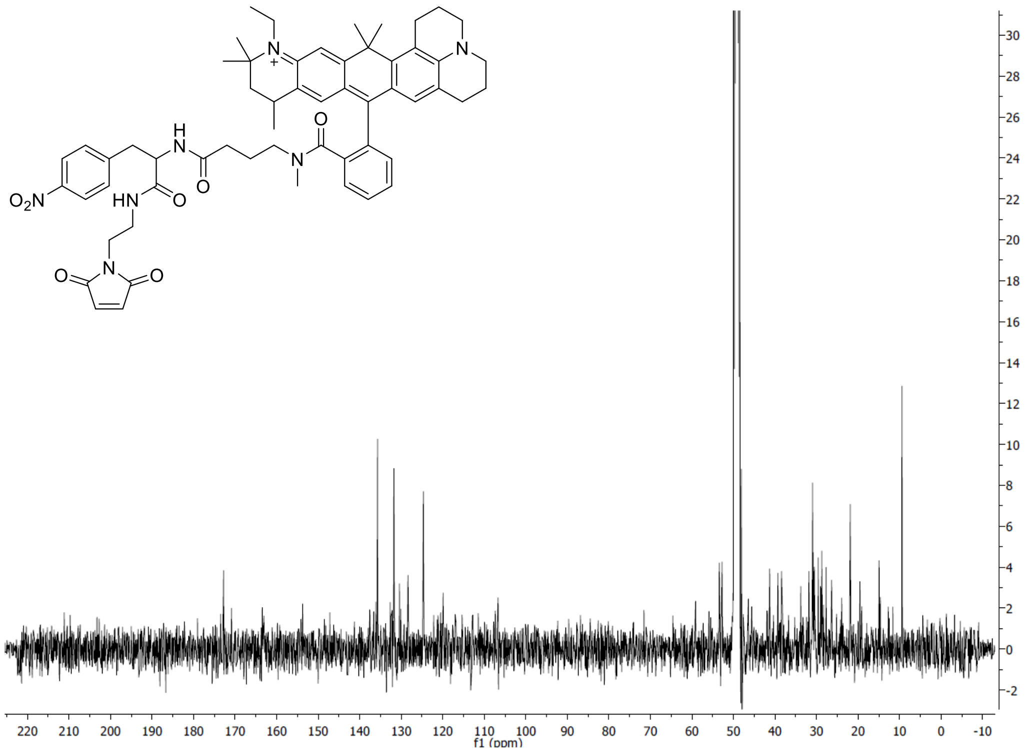

Supplementary Figure 30. ^{13}C-NMR of ATTO647N-NPA-Maleimide (**24**).

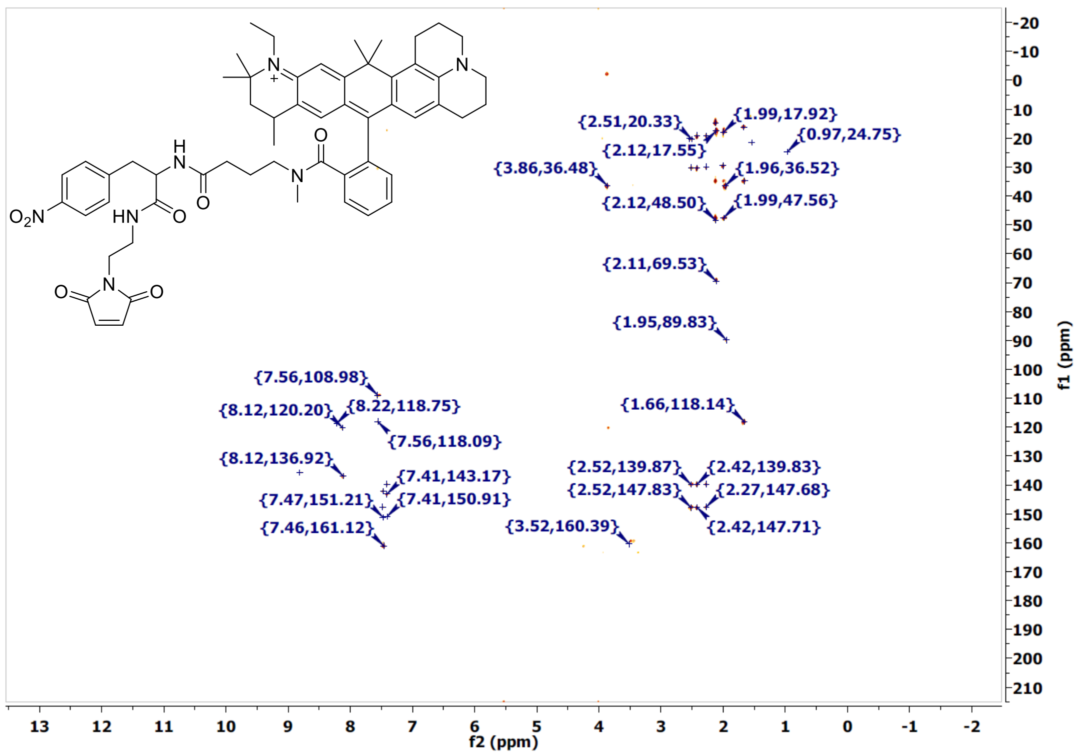

Supplementary Figure 31. GHMBC-NMR ATTO647N-NPA-Maleimide (**24**).

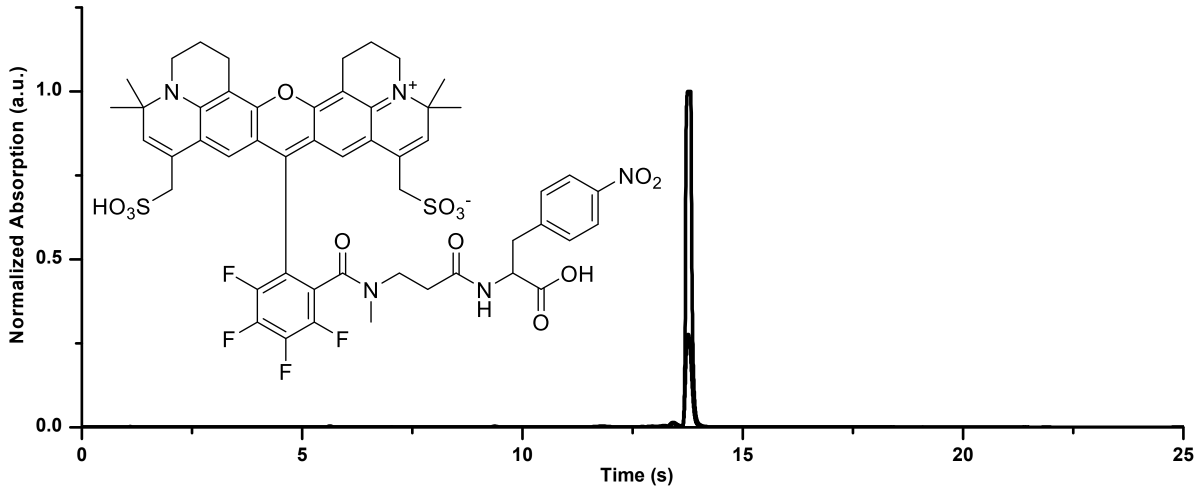

Supplementary Figure 32. HPLC-analysis of NPA-KK114 (**28**).

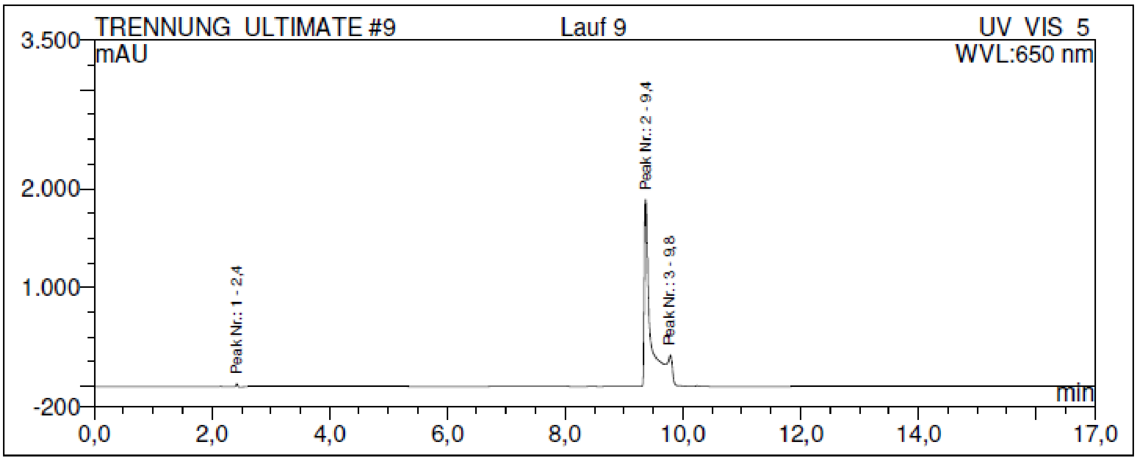

Supplementary Figure 33. HPLC-analysis of and β-Ala-NPA-KK114-OH (**31**)

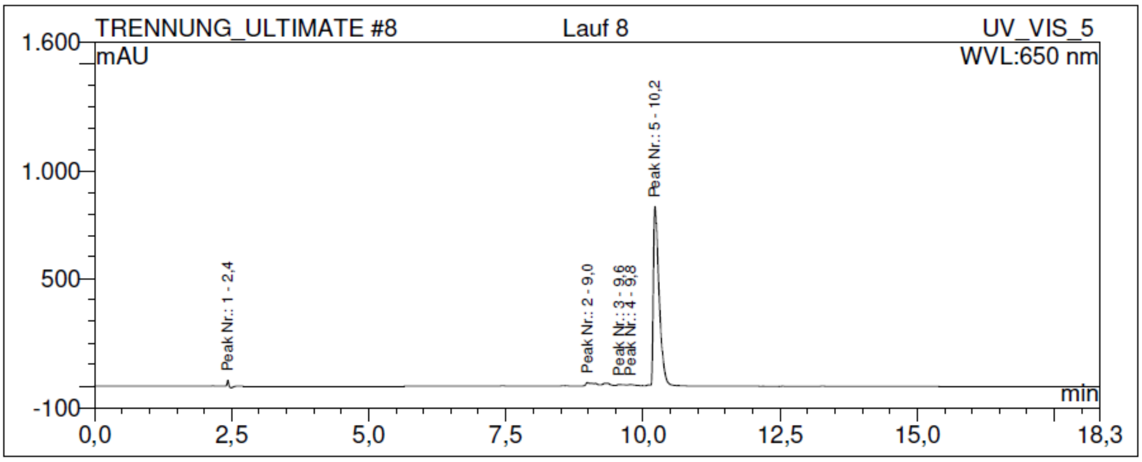

Supplementary Figure 34. HPLC-analysis of and β-Ala-NPA-KK114-NHS (**33**).

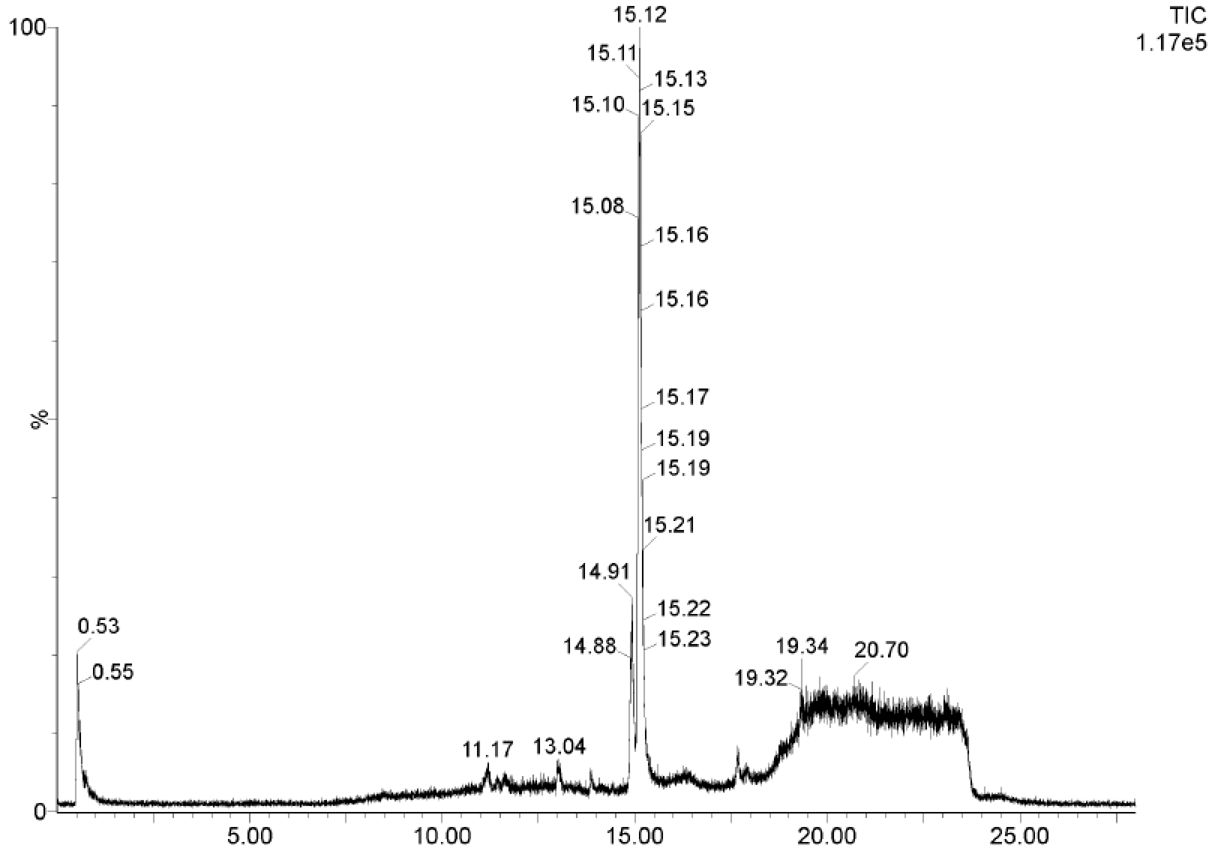

Supplementary Figure 35. LCMS of ATTO647N-NPA-Maleimide (**25**).

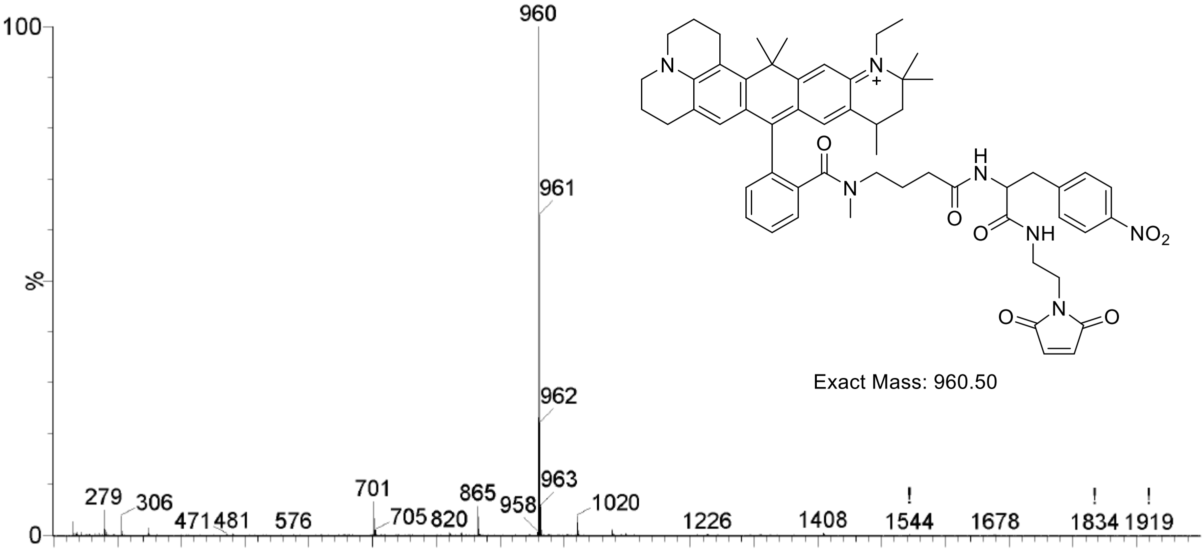

Supplementary Figure 36. LCMS-TOF MS ES+ of ATTO647N-NPA-Maleimide (**25**).
